# New Eremophilane-Type Sesquiterpenes from the Marine Sediment-Derived Fungus *Emericellopsis maritima* BC17 and Their Cytotoxic and Antimicrobial Activities

**DOI:** 10.3390/md21120634

**Published:** 2023-12-11

**Authors:** Jorge R. Virués-Segovia, Carlos Millán, Cristina Pinedo, Victoria E. González-Rodríguez, Sokratis Papaspyrou, David Zorrilla, Thomas A. Mackenzie, María C. Ramos, Mercedes de la Cruz, Josefina Aleu, Rosa Durán-Patrón

**Affiliations:** 1 Departamento de Química Orgánica, Facultad de Ciencias, Universidad de Cádiz, Puerto Real, 11510 Cádiz, Spain; jorge.roca@uca.es (J.R.V.-S.); carlos.millanlago@alum.uca.es (C.M.); cristina.pinedo@uca.es (C.P.); 2 Instituto de Investigación en Biomoléculas (INBIO), Universidad de Cádiz, Puerto Real, 11510 Cádiz, Spain; 3 Laboratorio de Microbiología, Departamento de Biomedicina, Biotecnología y Salud Pública, Facultad de Ciencias del Mar y Ambientales, Universidad de Cádiz, Puerto Real, 11510 Cádiz, Spain; victoriaeugenia.gonzalez@uca.es; 4 Departamento de Biología, Facultad de Ciencias del Mar y Ambientales, Universidad de Cádiz, Puerto Real, 11510 Cádiz, Spain; sokratis.papaspyrou@uca.es; 5 Departamento de Química Física, Facultad de Ciencias, Universidad de Cádiz, Campus Universitario Puerto Real s/n, Puerto Real, 11510 Cádiz, Spain; david.zorrilla@uca.es; 6 Centro de Excelencia en Investigación de Medicamentos Innovadores en Andalucía, Fundación MEDINA, 18016 Granada, Spain; thomas.mackenzie@medinaandalucia.es (T.A.M.); carmen.ramos@medinaandalucia.es (M.C.R.); mercedes.delacruz@medinaandalucia.es (M.d.l.C.)

**Keywords:** *Emericellopsis maritima*, marine-derived fungus, OSMAC strategy, eremophilanes, diketopiperazines, cytotoxicity, antibacterial, antifungal

## Abstract

The fungal strain BC17 was isolated from sediments collected in the intertidal zone of the inner Bay of Cadiz and characterized as *Emericellopsis maritima*. On the basis of the one strain–many compounds (OSMAC) approach, four new eremophilane-type sesquiterpenes (**1**–**4**), together with thirteen known derivatives (**5**–**17**) and two reported diketopiperazines (**18**, **19**), were isolated from this strain. The chemical structures and absolute configurations of the new compounds were determined through extensive NMR and HRESIMS spectroscopic studies and ECD calculation. Thirteen of the isolated eremophilanes were examined for cytotoxic and antimicrobial activities. PR toxin (**16**) exhibited cytotoxic activity against HepG2, MCF-7, A549, A2058, and Mia PaCa-2 human cancer cell lines with IC_50_ values ranging from 3.75 to 33.44 µM. (+)-Aristolochene (**10**) exhibited selective activity against the fungal strains *Aspergillus fumigatus* ATCC46645 and *Candida albicans* ATCC64124 at 471 µM.

## 1. Introduction

Marine fungi are recognized as rich producers of structurally interesting and biologically active natural products [1,2]. Recently, the sequence data of fungal genomes have demonstrated the presence of cryptic biosynthetic pathways, which are not always expressed under standard laboratory conditions [3]. As a result, modification of growth conditions may be of great value for the discovery of novel and useful bioactive metabolites.

The one strain–many compounds (OSMAC) approach, conceptualized by Bode et al. [4], has been successfully applied to increase the chemical diversity and yield of new natural products from a single microbial strain. According to this approach, each microbial strain is cultured in a variety of media and/or under different culture conditions to induce the production of cryptic metabolites [4,5]. For example, cultivation of the marine-derived fungus *Penicillium adametzioides* AS-53 on rice medium produced acorane sesquiterpenes, while growth on potato dextrose broth (PDB) gave rise to a new spiroquinazoline derivative, *N*-formyllapatin A, along with two new bisthiodiketopiperazine derivatives, adametizines A and B [6,7]. Recently, nine meroterpenoids, peniciacetal A-I, and five analogues were isolated from the mangrove endophytic fungus *Penicillium* sp. HLLG-122 by altering the composition of the culture medium according to the OSMAC approach [8].

As part of our ongoing research to search for new bioactive compounds from marine-derived fungi, this work includes the isolation of the fungal strain BC17 from sediment samples from the intertidal zone of the inner Bay of Cadiz (Cádiz, Spain) and its identification as *Emericellopsis maritima*. In recent studies, we evaluated the antitumor and antioxidant activity of fractions derived from the extract of the culture of this strain in rice medium. Three of these fractions displayed potent cytotoxic activity against two colorectal cancer (CRC) cell lines, T84, and SW480, with no activity in the non-tumour line. In addition, one of them demonstrated a strong antioxidant capacity, suggesting a potential role of marine fungi in combating oxidative stress, a factor contributing to CRC development and progression [9].

In order to find new potentially bioactive compounds, the chemical constituents of *E. maritima* BC17 were examined following an OSMAC approach. In this paper, we report the isolation and structural elucidation of four new eremophilane-type sesquiterpenes (**1**–**4**), thirteen known eremophilane derivatives (**5**–**17**), and two known diketopiperazines (**18**, **19**). Their chemical structures were established based on the interpretation of their spectroscopic data, including HRESIMS and 1D and 2D NMR. In addition, the cytotoxic and antimicrobial activities of these compounds were evaluated.

## 2. Results and Discussion

Fungal strain BC17 was isolated from surface sediments collected in the intertidal zone of the inner Bay of Cadiz (Cádiz, Spain). This strain was identified as *Emericellopsis maritima*, using morphological and molecular methods, by the identification service of the Spanish Type Culture Collection (CECT, https://www.uv.es/cect, accessed on 5 December 2023). The isolate exhibits the macroscopic and microscopic characteristics typical of the specified species [10]. Growth at 37 °C is negative. The presence of large conidia [6–(6.75)–8 × 3–(3.45)–4 μm], within the range described for the species *E. maritima* [(6.5–8 (–9) × 2.5–3.3(–4) μm], is noteworthy.

**Figure 1:**
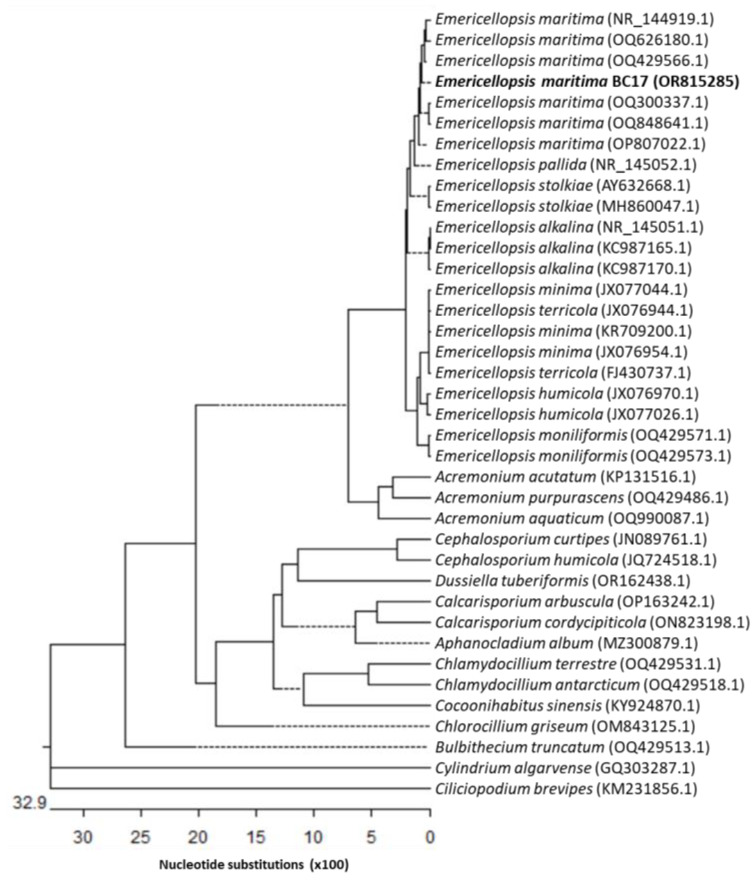
Neighbour-joining tree derived from ITS1 and ITS2, including the 5.8S rRNA gene sequences.

**Figure 2:**
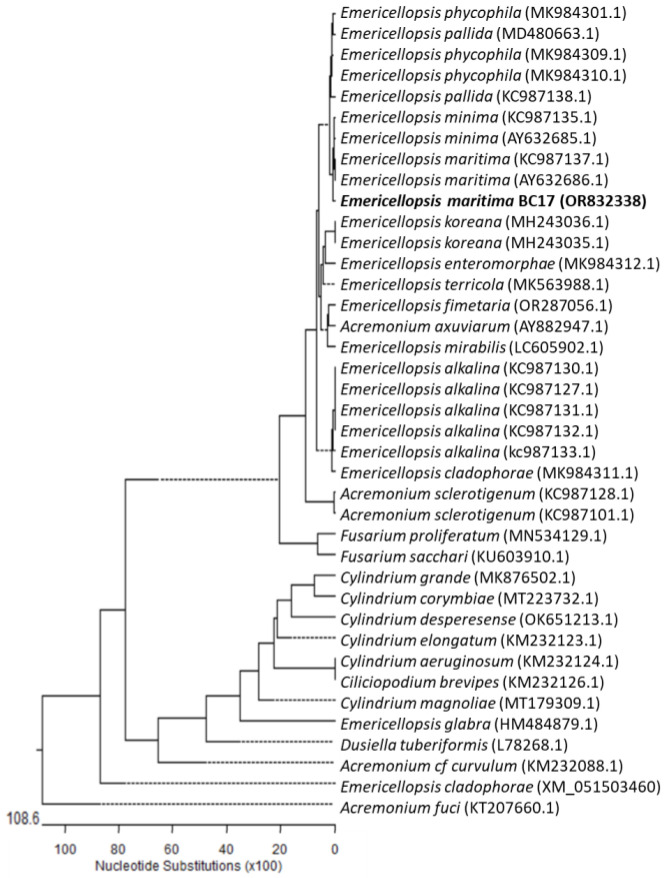
Neighbour-joining tree derived from *β*-tubulin gene sequences.

Neighbour-joining phylogenetic analysis was conducted using the Kimura two-parameter model and a bootstrap test with 5000 runs (MegAlign, DNASTAR^®^ Lasergene package). Sequences of related fungal species/genera were downloaded from the GenBank database, from the class Hypocreales, to which *Emericellopsis* belongs. The phylogenetic tree shown in Figure 1 was constructed using thirty-eight sequences, including twelve genera and twenty-four species, and the ribosomal DNA region comprising the intergenic spaces ITS1 and ITS2, including the 5.8S rRNA. Strain BC17 is clearly grouped with the species *E. maritima*. The phylogenetic tree shown in Figure 2 was constructed using thirty-nine sequences, including seven genera and twenty-seven species, of the *β*-tubulin (tub2) partial gene. Amplification and subsequent partial sequencing of the 28S rRNA (LSU) gene was also carried out, but this region does not allow resolution at the species level within the *Emericellopsis* clade. Based on all these studies, it was determined that isolate BC17 is clearly grouped with the species *E. maritima* (Figure 1 and Figure 2).

Based on the OSMAC approach, a systematic manipulation of nutritional factors, altering cultivation parameters and media composition was carried out to induce the expression of silent biosynthetic genes and the production of cryptic metabolites by the marine-derived fungus *E. maritima* BC17. For this purpose, the strain BC17 was cultivated on different solid culture media [malt agar (MA), potato dextrose agar (PDA), and rice medium] and incubated for 28 days.

**Figure 3:**
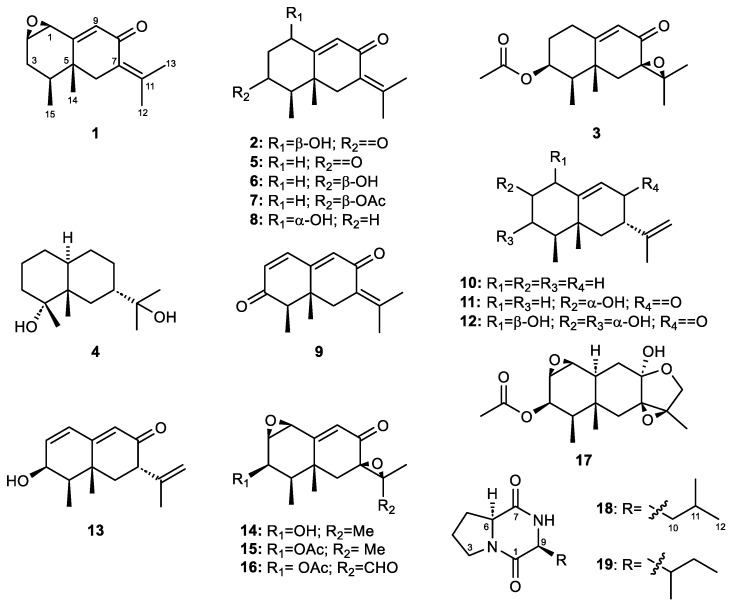
Eremophilane-type sesquiterpenes (**1**–**17**) and diketopiperazines (**18**, **19**) isolated from *Emericellopsis maritima* BC17.

Broths were extracted with ethyl acetate and the extracts were fractionated through column chromatography. Final HPLC purification of the fractions of interest from the three extracts led to four new eremophilane-type sesquiterpenes (**1**–**4**), thirteen known eremophilane derivatives (**5**–**17**), and two known diketopiperazines (**18**, **19**)—all isolated for the first time from the marine-derived fungus *E. maritima*. Specifically, the MA fermentation broth yielded compounds **1**–**11** and **13**–**19**; the PDA fermentation broth, compounds **8**, **10**, and **12**; and the rice medium fermentation broth, compounds **5**–**6** and **8** (Figure 3). Therefore, the alteration of the fermentation medium affected the chemical profile of this fungus, with the MA fermentation broth showing the highest chemodiversity.

The known compounds were identified through analysis of their 1D and 2D spectra and comparison of their spectroscopic data (Tables S1–S5) and optical activities (Table S6) with those reported in the literature for isopetasone (**5**) [11,12], (+)-3-epiisopetasol (**6**) [11,13], (3*S*)-3-acetoxyeremophil-7(11),9(10)-dien-8-one (**7**) [14], 1*α*-hydroxydehydrofukinone (**8**) [15], warburgiadione (**9**) [12,16], (+)-aristolochene (**10**) [17,18,19], 7-hydroxy-4a,5-dimethyl-3-prop-1-en-2-yl-3,4,5,6,7,8-hexahydronaphthalen-2-one (**11**) [20], guignarderemophilane F (**12**) [21], 3*β*-hydroxy-7*β*H-eremophil-1(2),9(10),11(12)-trien-8-one (**13**) [22], eremofortin A alcohol (**14**) [23], eremofortin A (**15**) [24], PR toxin (**16**) [24,25], eremofortin D (**17**) [24], cyclo-(l-Pro-l-Leu) (**18**) [26,27,28], and cyclo-(l-Pro-l-Ile) (**19**) [26,28].

Compound **1** was isolated as a white solid with a molecular formula C_15_H_20_O_2_, according to the ion peak at *m*/*z* 233.1557 [M+H]^+^ (calcd. for C_15_H_21_O_2_, 233.1542) observed in its HRESIMS. Its ^1^H NMR and ^13^C NMR data (Table 1 and Table 2) showed a characteristic pattern of signals of an eremophilane-type sesquiterpene, resembling the structures of compounds **5**–**17**. The ^13^C NMR spectrum of **1** (Figure S2) exhibited the presence of four methines, two of them bounded to oxygen, two methylenes, four methyl groups, and five quaternary carbons, one of which corresponded to a carbonyl group (*δ*_C_ 190.6 ppm) (Table 2). The ^1^H NMR spectrum (Figure S1) showed, in addition to the signals for four methyl groups at *δ*_H_ 0.90 (3H, d, *J* = 6.7 Hz), 1.01 (3H, d, *J* = 0.9 Hz), 1.84 (3H, d, *J* = 1.9 Hz), and 2.13 (3H, d, *J* = 1.9 Hz), the signal characteristic of one proton on a trisubstituted double bond at *δ*_H_ 6.17 (1H, s, H-9) (Table 1). The spectrum of **1** was very similar to that of compound **8** [15] except for the shielding of the signal for H-1 from *δ*_H_ 4.33 to 3.38 ppm and the presence of a signal at *δ*_H_ 3.52 ppm. These signals, which are coupled to each other, were assigned to the protons of an oxirane ring due to the absence, in the IR spectrum, of the band characteristic of free hydroxyl groups in the 3200–3600 cm^−1^ region. The HMBC correlations from H-1 to C-2, C-5, C-9, and C-10 and from H-2 to C-3 and C-4 located the oxirane ring between C-1 and C-2 (Figure S5). Consequently, the structure for compound **1** is proposed as 1-epoxyeremophil-7(11),9-dien-8-one.

**Figure 4:**
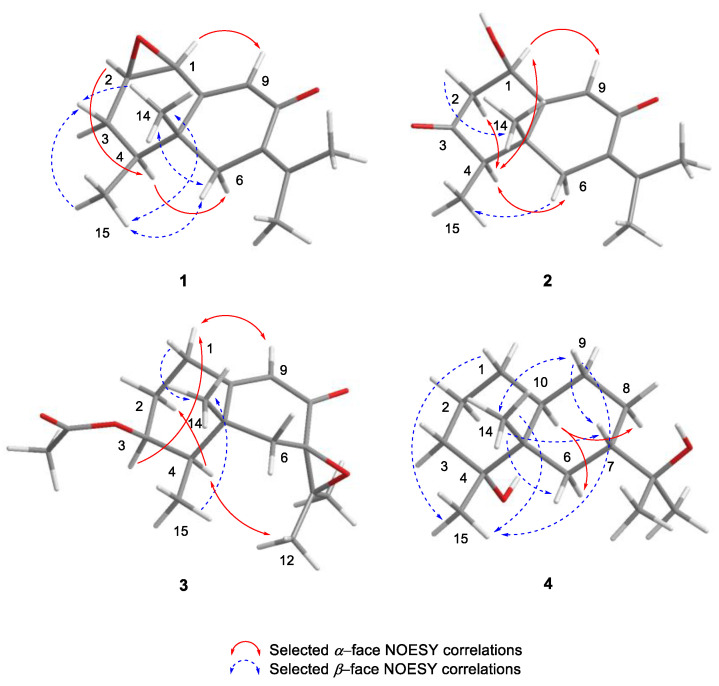
Selected NOESY correlations exhibited by **1**–**4**.

The relative configuration of **1** was elucidated on the basis of correlations observed in the 1D NOESY experiments (Figure S6a–g) and comparisons with those described for previously reported natural eremophilanes [11,12,13,14,15,16]. Compounds **5**–**9** showed *β*-orientations for H_3_-14 and H_3_-15. Since compounds **1** and **5**–**9** were cometabolites, **1** should have identical orientations of methyl groups to those of **5**–**9**. NOESY correlations observed for H-1*α*/H-9, H-2*α*/H-4*α*, and H-4*α*/H-6*α* indicated that these protons were on the same side of the ring, whereas the correlations between H_3_-15*β*/H-3*β*, H_3_-15*β*/H-6*β*, H_3_-15*β*/H_3_-14*β*, and H_3_-14*β*/H-6*β* were used to place these protons on the opposite face of the skeleton (Figure 4). All of these observations implied that the methyl groups H_3_-14 and H_3_-15 and the 1,2-epoxy ring are *β*-oriented.

**Figure 5:**
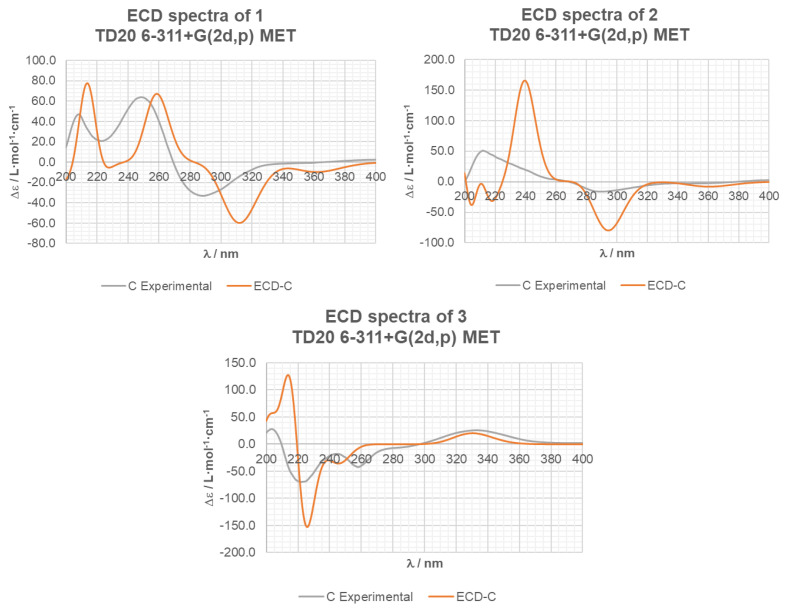
Experimental and calculated ECD spectra for compounds **1**–**3**. Calculations were performed with the conformers shown in Figure 4.

Absolute stereochemistry was established by comparing the experimental electronic circular dichroism (ECD) spectrum of compound **1** with the ECD spectrum predicted from quantum mechanical time-dependent density functional theory (TD-DFT) calculations [29]. In the 200–400 nm region, the theoretically calculated ECD spectrum of **1** was in good agreement with the experimental ECD spectrum (Figure 5). Consequently, the structure for compound **1** was assigned as (1*S*,2*R*,4*S*,5*R*)-1-epoxyeremophil-7(11),9-dien-8-one.

Compound **2** was isolated as a yellow oil whose molecular formula was established as C_15_H_20_O_3_ through HRESIMS (*m*/*z* 247.1342 [M-H]^−^, calcd. for C_15_H_19_O_3_, 247.1334). The pattern of signals in its ^1^H NMR and ^13^C NMR spectra (Figures S8 and S9) revealed the presence of two carbonyl groups (*δ*_C_ 208.0 and 190.9 ppm), two double bonds (*δ*_C/H_ 129.1/6.09, 161.9, 146.9, and 126.6), and four methyl groups (*δ*_C/H_ 23.0/2.16, 22.7/1.89, 20.2/1.11, and 7.4/1.12). Its spectroscopic data (Table 1 and Table 2) were in accord with a structure similar to that proposed for isopetasone (**5**). The main difference between the ^1^H NMR spectrum of **2** and that of the previously described eremophilene **5** was the presence of a geminal proton to a hydroxyl group at *δ*_H_ 4.83 (q), which correlated to a methine group at *δ*_C_ 73.2 in the HSQC experiment. The hydroxyl group was located at C-1 on the basis of the gHMBC correlations (Figure S12) of the protons H-9, H-2*α*, and H-2β to C-1. Analysis of gCOSY, gHSQC, and gHMBC spectra led to the assignment of all the proton and carbon signals, confirming the structure assigned to compound **2**.

The relative configuration of the molecule was determined from the 1D and 2D NOESY experiments (Figures S13 and S14a–f) and comparisons with those described for the previously reported eremophilanes [11,12]. Thus, the correlations of H-1*α* with H-9 and H-4*α*, H-2*α* with H-4*α*, and H-4*α* with H-6*α* indicated that these protons were on the same face of the molecule. On the other face were H-2*β*, H-6*β*, and methyl groups H_3_-14 and H_3_-15, as supported by the NOESY correlations between H-2*β* and H_3_-14*β*, and H-6*β* and H_3_-15*β* (Figure 4).

Its absolute configuration was studied using Mosher’s method and through a comparison of the calculated ECD spectrum with the experimental one. Compound **2** was treated with (*R*)-*α*-methoxy phenyl acetic acid (*R*(−)-MPA) and 1-ethyl-3-(3-dimethylaminopropyl)carbodiimide (EDC). Unfortunately, the MPA ester could not be achieved from the hydroxyl group at C-1. Instead, the elimination product **9** was obtained, confirming the *β*-orientation of the methyl groups H_3_-14 and H_3_-15. The ECD curve for the (1*R*,4*R*,5*R*) stereoisomer, calculated with the TD-DFT theoretical method, matched well with the experimental ECD spectrum (Figure 5). As a result, the absolute structure of compound **2** was established as (1*R*,4*R*,5*R*)-1-hydroxyeremophil-7(11),9-dien-3,8-dione.

Compound **3** was isolated as an amorphous solid. The molecular formula was established as C_17_H_24_O_4_ on the basis of an HRESIMS peak at *m*/*z* 315.1546 [M+Na]^+^ (calcd. for C_17_H_24_O_4_Na, 315.1572), consistent with six degrees of unsaturation. Its ^1^H NMR and ^13^C NMR spectra (Figures S16 and S17) also displayed characteristic resonances of an eremophilane-type sesquiterpene. In particular, the ^13^C NMR spectrum of compound **3** showed signals in close agreement with the known eremophilane derivative **7** [14], except for the lack of the characteristic signals of the Δ^7(11)^ double bond [*δ*_C_ 143.6 (C-11) and 127.6 (C-7) in **7**]. Instead, two quaternary carbons at *δ*_C_ 64.8 and 65.4 ppm (Table 2) attached to an oxygen function were observed, pointing to the presence of a 7,11-epoxy. The position of the oxirane ring was confirmed by the correlations observed in the gHMBC experiment (Figure S20) from H-6*α*/H-6*β* to C-4, C-7, C-8, C-10, and C-14; H-9 to C-1, C-5 and C-7; H_3_-12 to C-7 and C-13; and from H_3_-13 to C-7, C-11, and C-12. As a result, the structure for compound **3** was proposed as 3-acetoxy-7(11)-epoxyeremophil-9-en-8-one.

The relative configuration of **3** was elucidated on the basis of correlations observed in the 1D and 2D NOESY experiments (Figures S21 and S22a–f) and comparisons with those described for previously reported natural eremophilanes [14]. As depicted in Figure 4, the NOESY correlations observed for H-9/H-1*α*, H-3*α*/H-1*α*, and H-4*α*/H-2*α* suggested that these protons are *α*-oriented, whereas the correlations of H-1*β* with H_3_-14*β* and H_3_-15*β* with H_3_-14*β* placed these protons on the *β* face of the skeleton. The 7,11-epoxy ring was assigned *β*-oriented on the basis of the NOESY cross-peak between H-4*α* and H_3_-12.

This compound possesses an *α*,*β*-unsaturated ketone chromophore. As a result, its absolute configuration could be determined from the Cotton effects observed in its ECD. The positive Cotton effect at 332 nm and the negative Cotton effects at 223 and 247 nm observed in the experimental ECD spectrum of **3** were consistent with those observed in the spectrum predicted from TD-DFT calculations (Figure 5). Accordingly, the structure of compound **3** was assigned as (3*S*,4*R*,5*R*,7*R*)-3-acetoxy-7(11)-epoxyeremophil-9-en-8-one.

Compound **4** was assigned a molecular formula of C_15_H_28_O_2_ on the basis of the observed sodium adduct ion in its HRESIMS (*m*/*z* 263.1994 [M+Na]^+^, calcd. for C_15_H_28_O_2_Na, 263.1987), which was consistent with two degrees of unsaturation. The IR spectrum exhibited an absorption band at 3394 cm^−1^, characteristic of hydroxyl groups. The ^13^C NMR spectrum (Figure S25) revealed the presence of 15 carbon resonances encompassing four methyl groups, six methylene groups, two methine groups, and three non-protonated carbons, two of which bore an oxygen function (Table 2). The ^1^H NMR spectrum showed four singlet methyl groups at *δ*_H_ 1.17 (H_3_-12), 1.16 (H_3_-13), 1.08 (H_3_-15), and 0.88 (H_3_-14) (Table 1), characteristic of a C-4 and C-11 tetrasubstituted eremophilane derivative. The decaline unit was deduced through a comprehensive analysis of its 2D NMR spectra. Selective 1D TOCSY spectra (Figure S30a–d) performed as a function of mixing time for signals at *δ*_H_ 1.73 (H-1β), 1.43 (H-6β), and 1.22 (H-10*α*) identified two sequences of coupled spin systems, H_2_-1/H_2_-2/H_2_-3 and H_2_-6/H-7/H_2_-8/H_2_-9/H-10. These data, together with the HMBC correlations (Figure S28) from H-1*β* to C-2 and C-3; H-2*α*/H-2*β* to C-3 and C-4; H-3*β* to C-4 and C-5; H_3_-15*β* to C-4 and C-5; and from H_3_-14*β* to C-5 and C-10, established the structure of decaline ring A with two tertiary methyl groups at C-4 and C-5, and located one of the hydroxyl groups at the position C-4. On the basis of HMBC correlations from H-6*β* to C-4, C-5, C-14, C-10, C-7, and C-8; from H-8*β* to C-9 and C-11; from H-9*α* to C-10 and C-5; from H-9*β* to C-8 and C-7; and from H-10*α* to C-4, C-5, and C-14, the structure of ring B was also established. In addition, HMBC correlations from the methyl groups H_3_-12 and H_3_-13 to the methine carbon C-7 and the oxygenated quaternary carbon C-11 indicated a 2-hydroxyisopropyl group attached at C-7.

The relative configuration of **4** was deduced from the 1D NOESY spectra (Figure S29a–e) and that described for the previously reported eremophilanes [25]. As depicted in Figure 4, the NOESY correlations observed between H_3_-14/H_3_-15, H_3_-14/H-6*β*, H_3_-14/H-7*β*, H_3_-14/H-9*β*, H-1*β*/H_3_-15, and H-9*β*/H_3_-15 indicated that the methyl groups at C-4 and C-5 were cofacial and *β*-oriented. The configuration at C-7 was assigned as *S* based on NOESY correlations between H_3_-14*β* and H-7*β*, and between H-9*β* and H-7*β*. On the other hand, H-10 showed NOESY correlations with H-6*α* and H-8*α*. Based on these correlations and the *α*-orientation of H-10 in its cometabolite **17 [27]**, the *trans* junction of the decaline ring was established. These assignments were consistent with the absolute configurations of the reported eremophilanes and allowed compound **4** to be proposed as (4*R*,5*S*,7*S*,10*S*)-eremophilane-4,11-diol.

The biological activity of fungal eremophilanes was reviewed by Yuyama et al. in 2018 [30]. This family of compounds mainly shows phytotoxic, antifungal, antibiotic, and anticancer activities, although some derivatives have also shown antiviral, anti-inflammatory, anti-obesity, and immunomodulatory activities. Thirteen of the isolated compounds (**1**, **3**, **5**–**6**, **8**–**11**, **13**–**17**) were therefore evaluated for antimicrobial and cytotoxic activities. They were tested in triplicates against a panel of eight human pathogens, including both Gram-negative and Gram-positive bacteria (*Acinetobacter baumannii* ATCC19606, *Pseudomonas aeruginosa* PAO-1, *Klebsiella pneumoniae* ATCC700603, *Escherichia coli* ATCC25922, methicillin-resistant *Staphylococcus aureus* MB5393, and sensitive *S. aureus* ATCC29213), yeast (*Candida albicans* ATCC64124), and fungi strains (*Aspergillus fumigatus* ATCC46645). No antibacterial or antifungal properties were detected for these compounds against this panel of human pathogens, except for aristolochene (**10**), which exhibited selective activity against the two fungal strains at the highest concentration tested of 471 µM (Table 3).

The antiproliferative activities of these compounds (**1**, **3**, **5**–**6**, **8**–**11**, **13**–**17**) were also tested against liver (HepG2, ATCC HB-8065), breast (MCF-7, ATCC HTB-22), lung (A549, ATCC CCL-185), skin (A2058, ATCC CRL-3601), and pancreas (Mia PaCa-2, ATCC CRL-1420) human cancer cells using MTT test. Methyl methanesulfonate (MMS) 4mM was used as a positive control of cell death and doxorubicin was included as a known chemotherapeutic agent. Among the eremophilanes tested, compound **16** was active against all of the tested cell lines with IC_50_ values ranging from 3.75 to 33.44 µM (Table 4). Previous studies reported that the PR toxin (**16**) inhibited human intestinal epithelial (Caco-2) cells with IC_50_ values in the range of 1–13 µg/mL [31]. In addition, Darsih et al. [32] demonstrated that **16** affected the viability of the cholangiocarcinoma (HuCCA-1), cervical carcinoma (HeLa), hormone-dependent (T47D), and hormone-independent breast cancer (MDA-MB231), acute T-lymphoblastic leukemia (MOLT-3), and promyelotic leukemia (HL-60) human cell lines with IC_50_ values ranging from 0.06 to 2.19 mM. According to Moulé et al., the aldehyde group present in the PR toxin (**16**) structure on C-12 is mainly responsible for its biological activity [33]. This statement is confirmed by the results obtained in this work, where the PR toxin is the only cytotoxic eremophilane and with an aldehyde group of all those tested.

## 3. Materials and Methods

### 3.1. General Experimental Procedures

Melting points were measured with a Reichert–Jung Kofler block. Optical rotations were determined with a JASCO P-2000 polarimeter. Infrared spectra were recorded on a PerkinElmer Spectrum BX FT-IR spectrophotometer and reported as wave number (cm^−1^). ECD spectra were recorded on a JASCO J-1500 CD spectrometer. ^1^H and ^13^C NMR measurements were recorded on Agilent 400 and 500 MHz and Bruker 700 MHz NMR spectrometers with SiMe_4_ as the internal reference. Chemical shifts are expressed in ppm (*δ*), referenced to CDCl_3_ (Eurisotop, Saint-Aubiu, France, *δ*_H_ 7.25, *δ*_C_ 77.0) and CD_3_OD (Eurisotop, Saint-Aubiu, France, *δ*_H_ 3.30, *δ*_C_ 49.0). COSY, HSQC, HMBC, NOESY, and TOCSY experiments were performed using standard Agilent or Bruker pulse sequence. Spectra were assigned using a combination of 1D and 2D techniques. HRMS was performed in a Q-TOF mass spectrometer in the positive- or negative-ion ESI mode. TLC was performed on Merck Kiesegel 60 Å F_254_, 0.25 mm layer thickness. Silica gel 60 (60−200 µm, VWR) was used for column chromatography. Purification using HPLC was performed with a Merck-Hitachi Primade apparatus equipped with a UV−vis detector (Primaide 1410) and a refractive index detector (RI-5450), and a Merck-Hitachi LaChrom apparatus equipped with a UV−vis detector (L 4250) and a differential refractometer detector (RI-7490). LiChroCART LiChrospher Si 60 (5 μm, 250 mm × 4 mm), LiChroCART LiChrospher Si 60 (10 μm, 250 mm × 10 mm), and ACE 5 SIL (5 μm, 250 mm × 4.6 mm id) columns were used for isolation experiments.

### 3.2. Fungal Material and Identification

The marine-derived fungus *E. maritima* BC17 was isolated from intertidal sediments collected in the inner Bay of Cadiz (Cádiz, Spain) within a *Spartina* spp. bed with the permission of the national competent authority (ABSCH-CNA-ES-240784-3, reference number ESNC84). Surface sediment samples were collected aseptically in the field, stored in sterile packaging, kept on ice, brought to the laboratory, and immediately processed. Sediment was diluted with sterile seawater water (SSW) and aliquots were grown on PDA plates and marine agar plates (Condalab S.L.) and incubated at 25 °C for 5–10 days. Fungal colonies were selected and streaked on PDA plates under axenic conditions. The isolates were maintained on PDA at 25 °C for routine experiments and spores were stored in 60% (*v*/*v*) glycerol at −20 °C for later studies.

The BC17 fungal strain isolated was identified as *E. maritima* using the service of the Spanish Type Culture Collection (CECT, https://www.uv.es/cect, accessed on 5 December 2023) based on both phenotypic and molecular techniques. Three regions of the fungal genome were amplified through conventional PCR: (a) amplification and sequencing (with readings in both directions) of the ribosomal DNA region comprising the intergenic spaces ITS1 and ITS2, including the 5.8S rRNA (ITS5 5′-GGAAGTAAAAGTCGTAACAAGG-3′; ITS4 (5′-TCCTCCGCTTATTGATATGC-3′); (b) partial amplification and sequencing (with readings in both directions) of the 28S rRNA gene (LR0R 5′-GTACCCGCTGAACTTAAGC-3′; LR7 5′-TACTACCACCAAGATCT-3′); and (c) partial amplification and sequencing of the *β*-tubulin gene (with readings in both directions) (Bt2a 5′-GGTAACCAAATCGGTGCTGCTTTC-3′; Bt2b 5′-ACCCTCAGTGTAGTGACCCTTGGC-3′). The sequencing of these regions was compared with those in NCBI databases. Sequences were submitted to the NCBI database with the accession number OR815285 for the ITS region; OR835190 for the 28S rRNA gene; and OR832338 for the *β*-tubulin gene. To study the phylogenetic relationship of our isolate, other sequences of related genera and species from the class Hypocreales were downloaded from the GenBank database and included in the phylogenetic trees.

Culture of *E. maritima* BC17 has been deposited at the University of Cádiz, Mycological Herbarium Collection (UCA). Spore suspensions of this strain are maintained viable in 80% glycerol at −40 °C.

### 3.3. OSMAC-Based Cultivation Procedures

Using the OSMAC approach, *E maritima* BC17 was grown on three different solid culture media: MA (20 g glucose, 20 g malt extract, 20 g agar, and 1 g peptone, per litre of water, pH 6.5−7), PDA (Condalab, Madrid, Spain), and rice medium (80 g white rice per 100 mL of water). All the media were autoclaved before use. Rice medium ingredients were soaked overnight before autoclaving.

For MA and PDA media, strain BC17 was grown on Petri dishes, each containing 100 mL of the corresponding medium. Each plate was inoculated with one mycelium plug of 0.9 cm diameter from a seven-day culture on MA or PDA, and then incubated for 28 days at 24–26 °C under continuous white light (daylight lamp) for metabolite production.

Rice medium was also used to culture *E. maritima* BC17. Sixteen Erlenmeyers flasks (500 mL), containing 100 mL of rice medium, were inoculated with 750 µL of a conidial suspension obtained by adding 15 mL of sterile water to three Petri dishes (9 cm diameter) cultured with *E. maritima* BC17. The flasks were then incubated for 28 days under the conditions described above for metabolite production.

### 3.4. Extraction, Isolation, and Characterization of Eremophilane-Type Sesquiterpenes

The MA (3.6 L), PDA (3 L), and rice (1 L) fermentation broths were separately sonicated with ethyl acetate (×2) for 15 min at a frequency of 40 kHz using an AU-65 ultrasonic system (Argo Lab, Bogotá, Colombia) and then filtered. Before solid–liquid extraction, the MA and PDA media were cut into small pieces and the rice media was crushed. The organic extracts were dried over dry Na_2_SO_4_ and the solvent evaporated at reduced pressure to yield crude extracts of 1.597 g for MA, 0.599 g for PDA, and 4.196 g for rice medium.

The crude extracts were fractionated through silica gel column chromatography using an increasing gradient of ethyl acetate in *n*-hexane and methanol as eluents. Final purification of the different fractions was carried out using semipreparative HPLC.

The MA fermentation broth yielded, in addition to the known compounds **5** (1.6 mg), **6** (1.0 mg), **7** (4.0 mg), **8** (2.6 mg), **9** (1.0 mg), **10** (64.1 mg), **11** (1.9 mg), **13** (3.7 mg), **14** (0.6 mg), **15** (0.8 mg), **16** (2.4 mg), **17** (2.2 mg), **18** (3.4 mg), and **19** (2.8 mg), the new metabolites **1** (1.6 mg), **2** (1.6 mg), **3** (11.7 mg), and **4** (1.5 mg). The PDA fermentation broth afforded the known compounds **8** (2.2 mg), **10** (18.0 mg), and **12** (0.9 mg), while the rice fermentation broth gave the known compounds **5** (0.7 mg), **6** (0.8 mg), and **8** (0.7 mg).

(1*S*,2*R*,4*S*,5*R*)-1-Epoxyeremophil-7(11),9-dien-8-one (**1**): Purified through semipreparative HPLC (*n*-hexane:EtOAc 85:15, flow 3.0 mL/min, *t*_R_ = 32 min). White solid; mp 80.5 °C; ${\left[\alpha \right]}_{D\;}^{24}\;$= +63 (c 0.05, CHCl_3_); ECD (MeOH) *λ* (Δ*ε*) 208 (5.50), 249 (7.48), 289 (-3.90) nm; IR (film) ν_max_ 3413, 2926, 1662, 1229, 828 cm^−1^; ^1^H and ^13^C NMR data see Table 1 and Table 2; gHMBC (selected correlations) H-1*α* → C-2, C-5, C-9, C-10; H-2*α* → C-3, C-4; H3*α* → C-2, C-4; H-3*β* → C-2, C-15; H-4*α* → C-5, C-14, C-15; H-6*β* → C-1, C-4, C-7, C-8, C-10, C-11, C-14; H-9 → C-1, C-5, C-7; H_3_-12^d^ → C-7, C-11, C-13; H_3_-13^d^ → C-7, C-8, C-11, C-12; H_3_-14*β* → C-4, C-6, C-10, C-15; H_3_-15*β* → C-3, C-4; HRESIMS *m*/*z* 233.1557 [M+H]^+^ (calcd. for C_15_H_21_O_2_, 233.1542).

(1*R*,4*R*,5*R*)-1-Hydroxyeremophil-7(11),9-dien-3,8-dione (**2**): Purified through semipreparative HPLC (*n*-hexane:EtOAc 6:4, flow 1.0 mL/min, *t*_R_ = 25 min). Yellow oil; ${\left[\alpha \right]}_{D\;}^{23}\;$= +7 (c 0.06, CHCl_3_); ECD (MeOH) *λ* (Δ*ε*) 212 (2.63), 2.89 (-0.85) nm; IR (film) ν_max_ 3417, 2977, 1717, 1658, 1279 1224, 1064, 754 cm^−1^; ^1^H and ^13^C NMR data see Table 1 and Table 2; gHMBC (selected correlations) H-2*α* → C-1, C-3; H-2*β* → C-1, C-3, C-10; H-4*α* → C-3, C-5, C-6, C-14, C-15; H-6*β* → C-5, C-7, C-8, C-10, C-11, C-14; H-9 → C-1, C-5, C-7; H_3_-12^e^ → C-7, C-11, C-13; H_3_-13^e^ → C-7, C-11, C-12; H_3_-14*β* → C-6, C-10; H_3_-15*β* → C-3, C-4, C-5; HRESIMS *m/z* 247.1342 [M-H]^−^ (calcd. for C_15_H_19_O_3_, 247.1334).

(3*S*,4*R*,5*R*,7*R*)-3-Acetoxy-7(11)-epoxyeremophil-9-en-8-one (**3**): Purified through semipreparative HPLC (*n*-hexane:EtOAc 6:4, flow 3.0 mL/min, *t*_R_ = 42 min). Amorphous solid; ${\left[\alpha \right]}_{D\;}^{23}\;$= +72 (c 0.51, CHCl_3_); ECD (MeOH) *λ* (Δ*ε*) 223 (-0.50), 247 (-0.62), 332 (0.20) nm; IR (film) ν_max_ 2928, 1728, 1679, 1375, 1250, 895 cm^−1^; ^1^H and ^13^C NMR data see Table 1 and Table 2; gHMBC (selected correlations) H-1*β* → C-2, C-9, C-10; H-2*α* → C-1; H-3*α* → C-1, C-5; H-4*α* → C-6, C-14, C-15; H-6*α*, H-6*β* → C-4, C-7, C-8, C-10, C-14; H-9 → C-1, C-5, C-7; H_3_-12 → C-7, C-13; H_3_-13 → C-7, C-11, C-12; H_3_-14*β* → C-4, C-6, C-10; H_3_-15*β* → C-3, C-4; CH_3_COO → CH_3_COO; HRESIMS *m/z* 315.1546 [M+Na]^+^ (calcd. for C_17_H_24_O_4_Na, 315.1572).

(4*R*,5*S*,7*S*,10*S*)-Eremophilane-4,11-diol (**4**): Purified through semipreparative HPLC (*n*-hexane:EtOAc 48:52, flow 3.0 mL/min, *t*_R_ = 79 min). Amorphous solid; ${\left[\alpha \right]}_{D\;}^{26}\;$= +34 (c 0.06, CHCl_3_); IR (film) ν_max_ 3394, 2931, 1383, 757 cm^−1^; ^1^H and ^13^C NMR data see Table 1 and Table 2; gHMBC (selected correlations) H-1*β* → C-2, C-3; H-2*α*, H-2*β* → C-5, C-3, C-1, C-4; H-3*β* → C-5, C-10, C-4; H-6*β* → C-14, C-8, C-5, C-7, C-10, C-4; H-8*β* → C-9, C-11; H-9*α* → C-5, C-10; H-9*β* → C-8, C-7, C-6; H-10*α* → C-14, C-5, C-4; H_3_-12^h^ → C-13, C-7, C-11; H_3_-13^h^ → C-12, C-7, C-11; H_3_-14*β* → C-5, C-6, C-3, C-10; H_3_-15*β* → C-5, C-10, C-4; HRESIMS *m*/*z* 263.1994 [M+Na]^+^ (calcd. for C_15_H_28_O_2_Na, 263.1987).

### 3.5. Mosher’s Esterification Reaction of Compound ***2***

EDC (0.8 mg, 4.2 × 10^−3^ mmol), *N*,*N*-dimethylaminopyridine (DMAP) (0.5 mg, 4.0 × 10^−3^ mmol), and *R*(−)-MPA (0.8 mg, 4.6 × 10^−3^ mmol) were added to a stirred solution of compound **2** (0.5 mg, 2.0 × 10^−3^ mmol) in dry dichloromethane (1 mL). The resulting mixture was stirred for 3–4 h at room temperature. Then, the solvent was evaporated under reduced pressure and the residue was subjected to purification via column chromatography on silica gel to afford compound **9** (0.2 mg, 8.7 × 10^−4^ mmol, 44% yield).

### 3.6. Computational Details of ECD Calculations

The conformational analysis of compounds **1**–**3** was conducted through the application of the semiempirical PM6 method [34]. Quantum mechanical computations were executed utilizing the Gaussian 16 package [35]. A comprehensive geometric optimization was undertaken emploing density functional theory (DFT) within the framework of B3LYP functionals [36,37] and the 6−311+G(2d,p) basis set. Subsequently, calculations were performed to determine the energies, oscillator strengths, and rotational strengths associated with the initial 20 electronic excitations, employing the TD-DFT methodology [38,39]. The solvent’s influence (methanol) was considered within the calculations, incorporating the polarizable continuum model (PCM) with the implementation of the implicit solvation energy (IEF) approach [40,41,42]. To mimic the ECD spectrum of the conformer, a Gaussian function was used, featuring a half-bandwidth of 0.33 eV.

### 3.7. In Vitro Antimicrobial Assay

The antimicrobial activities of compounds (**1**, **3**, **5**–**6**, **8**–**11**, **13**–**17**) were evaluated against six bacterial and two fungal human pathogens. Antibacterial susceptibility of the compounds was tested against *A. baumannii* ATCC19606, *P. aeruginosa* PAO-1, *K. pneumoniae* ATCC700603, *E. coli* ATCC25922, methicillin-resistant *S. aureus* MB5393, and sensitive *S. aureus* ATCC29213, while antifungal activity was tested against *C. albicans* ATCC64124 and *A. fumigatus* ATCC46645 following previously described methodologies [43]. Briefly, each compound was serially diluted in 100% DMSO with a dilution factor of 2 to provide 10 final assay concentrations in all antimicrobial assays. The MIC was defined as the lowest concentration of the compound that inhibited ≥90% of the growth of a microorganism after overnight incubation. Genedata Screener software, version 18.0.4-Standard (Genedata, Inc., Basel, Switzerland) was used to process and analyze the data and to calculate the RZ factor, which predicts the robustness of an assay [43]. In all experiments performed in this work, the RZ factor obtained was between 0.89 and 0.98.

### 3.8. In Vitro Antitumor Assay

The tumour human cell lines used were liver HepG2 (ATCC HB-8065), breast MCF-7 (ATCC HTB-22), lung A549 (ATCC CCL-185), skin A2058 (ATCC CRL-3601), and pancreas Mia PaCa-2 (ATCC CRL-1420). Purified compounds were dissolved in DMSO 100% at 35 mM (compounds **1** and **9**), 29 mM (compound **10**), 27 mM (compound **3**), 26 mM (compounds **5**, **6**, **8**, **11**, and **13**), 19 mM (compounds **16** and **17**), 16 mM (compound **15**), and at 8 mM (compound **14**). The compounds were assayed for cytotoxicity using the manufacturer’s instructions for MTT. The MTT test is a colourimetric assay for measuring the activity of enzymes that reduce 3-(4,5-dimethylthiazol-2-yl)-2,5-diphenyltetrazolium bromide, a yellow tetrazole, to formazan dyes, giving a purple colour. Briefly, after 24 h, seeded cells were treated with compounds at a maximum dilution of 1/200 from the stocks in 10 points of 2-fold dilution per triplicate, for 72 h. MMS (Sigma Aldrich, St. Louis, MO, USA), at 4mM, was used as a positive control of cell death, DMSO 0.5% as a negative control and doxorubicin (Sigma Aldrich) was included as a known chemotherapeutic agent. After the incubation period, the plates containing treated cells were washed with 200 µL of phosphate-buffered saline (PBS) 1X (137 mM NaCl, 2.7 mM KCl, 10 mM Na_2_HPO_4_, 1.8 mM KH_2_PO_4_). Then, MTT dye (Thiazolyl blue tetrazolium bromide, ACROS Organics BV, Geel, Belgium) was added at 0.5 mg/mL and plates were incubated for 2 h. The supernatant was then removed and 100 µL of DMSO 100% were added to each well in order to dissolve the resulting formazan precipitates. Finally, absorbance was measured at 570 nm with an EnVision Multilabel Plate Reader (PerkinElmer, Waltham, CA, USA). Data obtained was analyzed using Genedata Screener Software inhibitory curves fit to a Hill Equation model to calculate IC_50_ and the confidence intervals at 95%.

## 4. Conclusions

In conclusion, we herein reported the isolation and identification of four previously undescribed eremophilane-type sesquiterpenes (**1**–**4**), eleven known eremophilane derivatives (**5**–**17**), and two known diketopiperazines (**18**, **19**) from the fungal strain *E. maritima* BC17. This strain was isolated from sediment samples from the intertidal zone of the inner Bay of Cadiz. Changes in the composition of the culture medium, based on the OSMAC fermentation optimization approach, induced the production of different metabolites, with the culture of strain BC17 on the MA medium for 28 days being the most favourable condition to expand *E. maritima* metabolome. Thirteen of the isolated eremophilanes were examined for cytotoxic and antimicrobial activities. Compound **16** exhibited cytotoxic activity against HepG2, MCF-7, A549, A2058, and Mia PaCa-2 human cancer cell lines with IC_50_ values ranging from 3.75 to 33.44 µM. Compound **10** exhibited selective activity against the fungal strains *Aspergillus fumigatus* ATCC46645 and *Candida albicans* ATCC64124 at 471 µM.

## Figures and Tables

**Table 1:** ^1^H NMR spectroscopic data for compounds **1**–**4**.

	1	2	3	4
Position	*δ*_H_, Mult (*J* in Hz) ^a^	*δ*_H_, Mult (*J* in Hz) ^a^	*δ*_H_, Mult (*J* in Hz) ^b^	*δ*_H_, Mult (*J* in Hz) ^c^
1*α*	3.38, d (3.7)	4.83, q (4.4)	2.20, m	1.39, dt (13.0, 6.6)
1*β*	-	-	2.73, td (13.5, 4.7)	1.73, dtd (13.0, 3.3, 1.6)
2*α*	3.52, dd (3.7, 5.7)	2.85, dd (15.2, 5.1)	1.67, tt (14.2, 3.8)	1.59, dt (13.5, 3.3) ^g^
2*β*	-	2.66, dd (15.1, 4.8)	2.19, ddt (14.2, 4.7, 2.3)	1.55, dq (13.5, 4.3) ^g^
3*α*	1.99, dt (15.5, 6.1)	-	5.12, q (3.4)	1.39, td (13.1, 4.3)
3*β*	1.80, dd (15.5, 12.6)	-	-	1.08, td (13.1, 4.3)
4*α*	1.64, td (12.6, 6.1)	2.53, q (6.6)	1.89, qd (7.0, 3.4)	-
6*α*	2.06, br d (13.4)	2.42, d (13.8)	2.04, d (15.0) ^f^	1.16, td (13.1, 4.2)
6*β*	2.81, d (13.4)	2.92, d (13.8)	2.10, d (15.0) ^f^	1.43, dt (13.1, 3.1)
7*β*	-	-	-	1.35–1.30, m
8*α*	-	-	-	1.37–1.28, m
8*β*	-	-	-	1.61, m
9*α*	6.17, s	6.09, d (0.9)	5.94, s	1.95, m 1.03, q (12.5)
9*β*				
10*α*	-	-	-	1.22, m
12	2.13, d (1.9) ^d^	2.16, d (2.1) ^e^	1.41, s	1.17, s ^h^
13	1.84, d (1.9) ^d^	1.89, d (1.4) ^e^	1.31, s	1.16, s ^h^
14*β*	1.01, d (0.9)	1.11, d (0.8)	1.32, s	0.88, s
15*β*	0.90, d (6.7)	1.12, d (6.6)	1.03, d (7.0)	1.08, s
OCOMe	-	-	2.12, s	-

^a^ 500 MHz, CDCl_3_; ^b^ 400 MHz, CDCl_3_; ^c^ 700 MHz, CD_3_OD; ^d–h^ Interchangeable assignments.

**Table 2:** ^13^C NMR spectroscopic data for compounds **1**–**4**.

	1	2	3	4
Position	*δ*_C_, Type ^a^	*δ*_C_, Type ^a^	*δ*_C_, Type ^b^	*δ*_C_, Type ^c^
1	55.0, CH	73.2, CH	28.3, CH_2_	44.2, CH_2_
2	55.7, CH	47.2, CH_2_	33.9, CH_2_	21.1, CH_2_
3	29.8, CH_2_	208.0, C	72.5, CH	42.3, CH_2_
4	39.1, CH	52.6, CH	42.7, CH	73.1, C
5	39.2, C	43.5, C	41.9, C	35.6, C
6	41.8, CH_2_	42.4, CH_2_	38.0, CH_2_	46.1, CH_2_
7	127.6, C	126.6, C	65.4, C	51.3, CH
8	190.6, C	190.9, C	195.0, C	23.5, CH_2_
9	131.9, CH	129.1, CH	124.1, CH	22.8, CH_2_
10	160.2, C	161.9, C	170.7, C	55.7, CH
11	145.3, C	146.9, C	64.8, C	73.4, C
12	23.0, ^d^ CH_3_	23.0, ^e^ CH_3_	21.3, CH_3_	27.5, ^f^ CH_3_
13	22.5, ^d^ CH_3_	22.7, ^e^ CH_3_	19.2, CH_3_	26.9, ^f^ CH_3_
14	16.0, CH_3_	20.2, CH_3_	23.8, CH_3_	19.2, CH_3_
15	14.6, CH_3_	7.4, CH_3_	12.2, CH_3_	22.5, CH_3_
OCOMe	-	-	170.3, C	-
OCOMe	-	-	21.3, CH_3_	-

^a^ 125 MHz, CDCl_3_; ^b^ 100 MHz, CDCl_3_; ^c^ 175 MHz, CD_3_OD; ^d–f^ Interchangeable assignments.

**Table 3:** Minimum inhibitory concentration (MIC) of eremophilanes **1**, **3**, **5**–**6**, **8**–**11**, and **13**–**17** against eight human pathogens.

	MIC (µM)							
Compound	*A. fumigatus* ATCC46645	*C. albicans* ATCC64124	*K. pneumonia* ATCC700603	*E. coli* ATCC25922	MSSA ATCC29213	MRSA MB5393	*A. baumannii* ATCC19606	*P. aeruginosa* PAO-1
**1**	>552	>552	>552	>552	>552	>552	>552	>552
**3**	>438	>438	>438	>438	>438	>438	>438	>438
**5**	>414	>414	>414	>414	>414	>414	>414	>414
**6**	>410	>410	>410	>410	>410	>410	>410	>410
**8**	>410	>410	>410	>410	>410	>410	>410	>410
**9**	>556	>556	>556	>556	>556	>556	>556	>556
**10**	471	471	>471	>471	>471	>471	>471	>471
**11**	>410	>410	>410	>410	>410	>410	>410	>410
**13**	>414	>414	>414	>414	>414	>414	>414	>414
**14**	>121	>121	>121	>121	>121	>121	>121	>121
**15**	>262	>262	>261	>261	>261	>261	>261	>261
**16**	>300	>300	>300	>300	>300	>300	>300	>300
**17**	>296	>296	>296	>296	>296	>296	>296	>296

**Table 4:** Half-maximal inhibitory concentration (IC_50_) of eremophilanes **1**, **3**, **5**–**6**, **8**–**11**, and **13**–**17** of cell viability in five tumour cell lines. Confidence intervals at 95% are shown in brackets as calculated with the Genedata© Screener software, version 18.0.4-Standard.

	IC_50_ (µM)				
Compound	HepG2	MCF7	A549	A2058	Mia PaCa-2
**1**	>172	>172	>172	>172	159 (142–172)
**3**	>137	>137	>137	>137	>137
**5**	>192	>129	>129	>129	>129
**6**	>128	>128	>128	>128	>128
**8**	>128	>128	>128	>128	>128
**9**	>174	>174	>174	>174	161 (139–187)
**10**	>147	>147	>147	>147	>147
**11**	>128	>128	>128	>128	>128
**13**	>129	>129	>129	125 (116–138)	>129
**14**	>38	>38	>38	>38	>38
**15**	>282	>82	>82	>82	>82
**16**	8.28 (7.88–8.72)	5.53 (5.31–5.78)	33.44 (31.56–35.00)	3.75 (3.44–4.38)	5.00 (4.69–5.31)
**17**	>93	>93	>93	74 (68–80)	>93
Doxorubicin	0.21 (0.19–0.23)	0.21 (0.16–0.28)	0.90 (0.70–1.00)	0.10 (0.08–0.13)	0.43 (0.36–0.50)

## Data Availability

The data presented in this study are available in Supplementary Material.

## Supplementary Materials

The following supporting information can be downloaded at: https://www.mdpi.com/article/10.3390/md21120634/s1, Tables S1–S6: NMR data and optical activities of compounds **5**–**19**; Figures S1–S31: 1D and 2D NMR and HRESIMS spectra of compounds **1**–**4**.
