# Supplementary material for: New Eremophilane-Type Sesquiterpenes from the Marine Sediment-Derived Fungus Emericellopsis maritima BC17 and Their Cytotoxic and Antimicrobial Activities
Source: Mar Drugs. 2023 Dec 11;21(12):634. doi: 10.3390/md21120634 (PMC10745059; doi:10.3390/md21120634)
Supplement: Supplementary file 1 [file marinedrugs-21-00634-s001.zip › marinedrugs-2713222-supplementary materials.pdf]

New eremophilane-type sesquiterpenes from the marine sediment-derived fungus *Emericellopsis maritima* BC17 and their cytotoxic and antimicrobial activities

Jorge R. Virués-Segovia,[1] Carlos Millán,[1] Cristina Pinedo,[1] Victoria E. González-Rodríguez,[2] Sokratis Papaspyrou,[3] David Zorrilla,[4] Thomas A. Mackenzie,[5] María C. Ramos,[5] Mercedes de la Cruz,[5] Josefina Aleu,[1,*] Rosa Durán-Patrón[1,*]

[1]Departamento de Química Orgánica, Facultad de Ciencias, Instituto de Biomoléculas, Campus Universitario Puerto Real s/n, Torre sur, 4ª planta, Universidad de Cádiz, 11510 Puerto Real, Cádiz, Spain.

[2]Departamento de Biomedicina, Biotecnología y Salud Pública, Laboratorio de Microbiología, Facultad de Ciencias del Mar y Ambientales, Universidad de Cádiz, 11510 Puerto Real, Cádiz, Spain.

[3]Departamento de Biología, Facultad de Ciencias del Mar y Ambientales, Universidad de Cádiz, 11510 Puerto Real, Cádiz, Spain.

[4]Departamento de Química Física, Facultad de Ciencias, Universidad de Cádiz, Campus Universitario Puerto Real s/n, 11510, Puerto Real, Cádiz, Spain.

[5]Centro de Excelencia en Investigación de Medicamentos Innovadores en Andalucía, Fundación MEDINA, Granada, Spain.

[*]rosa.duran@uca.es, josefina.aleu@uca.es

Table of contents

Table S1. ^{1}H NMR spectroscopic data for compounds **5-11** in $CDCl_3$. 4

Table S2. ^{1}H NMR spectroscopic data for compounds **12-17** in $CDCl_3$. 5

Table S3. ^{13}C NMR spectroscopic data for compounds **5-11** in $CDCl_3$. 6

Table S4. ^{13}C NMR spectroscopic data for compounds **12-17** in $CDCl_3$. 7

Table S5. ^{1}H and ^{13}C NMR (500 and 125 MHz) spectroscopic data for compounds **18-19** in $CDCl_3$. 8

Table S6. Optical activities for compounds **5-8** and **10-19**. 9

Figure S1. ^{1}H NMR spectrum (500 MHz, $CDCl_3$) of compound **1**. 10

Figure S2. ^{13}C NMR spectrum (125 MHz, $CDCl_3$) of compound **1**. 11

Figure S3. gCOSY spectrum of compound **1**. 12

Figure S4. gHSQC spectrum of compound **1**. 13

Figure S5. gHMBC spectrum of compound **1**. 14

Figure S6a-g. 1D NOESY spectrum of compound **1**. 15

Figure S7. HRESIMS spectrum of compound **1**. 22

Figure S8. ^{1}H NMR spectrum (500 MHz, $CDCl_3$) of compound **2**. 23

Figure S9. ^{13}C NMR spectrum (125 MHz, $CDCl_3$) of compound **2**. 24

Figure S10. gCOSY spectrum of compound **2**. 25

Figure S11. gHSQC spectrum of compound **2**. 26

Figure S12. gHMBC spectrum of compound **2**. 27

Figure S13. 2D NOESY spectrum of compound **2**. 28

Figure S14a-f. 1D NOESY spectrum of compound **2**. 29

Figure S15. HRESIMS spectrum of compound **2**. 35

Figure S16. ^{1}H NMR (400 MHz, $CDCl_3$) spectrum of compound **3**. 36

Figure S17. ^{13}C NMR (100 MHz, $CDCl_3$) spectrum of compound **3**. 37

Figure S18. gCOSY spectrum of compound **3**. 38

Figure S19. gHSQC spectrum of compound **3**. 39

Figure S20. gHMBC spectrum of compound **3**. 40

Figure S21. 2D NOESY spectrum of compound **3**. 41

Figure S22a-f. 1D NOESY spectrum of compound **3**. 42

Figure S23. HRESIMS spectrum of compound **3**. 48

Figure S24a. ^{1}H NMR spectrum (700 MHz, CD_3OD) of compound **4**. 49

Figure S24b. Expansion of ^{1}H NMR spectrum (700 MHz, CD_3OD) of compound **4**. 50

Figure S25. ^{13}C NMR spectrum (175 MHz, CD_3OD) of compound **4**. 51

Figure S26. gCOSY spectrum of compound **4**. 52
Figure S27. gHSQC spectrum of compound **4**. 53
Figure S28. gHMBC spectrum of compound **4**. 54
Figure S29a-e. 1D NOESY spectra of compound **4**. 55
Figure S30a-d. 1D TOCSY spectra of compound **4**. 60
Figure S31. HRESIMS spectrum of compound **4**. 64

Table S1. ^{1}H NMR spectroscopic data for compounds **5-11** in $CDCl_3$.

	**5**	**6**	**7**	**8**	**9**	**10**	**11**
Position	***δ*H, Mult (*J* in Hz)**[a]	***δ*H, Mult (*J* in Hz)**[a]	***δ*H, Mult (*J* in Hz)**[b]	***δ*H, Mult (*J* in Hz)**[a]	***δ*H, Mult (*J* in Hz)**[a]	***δ*H, Mult (*J* in Hz)**[a]	***δ*H, Mult (*J* in Hz)**[a]
1*α*	2.79-2.68, m	2.12, ddd (14.4, 4.4, 2.6)	2.16, ddd (14.4, 4.5, 2.4)	-		1.42, m[h]	2.37, m
1*β*	2.79-2.68, m	2.76, tdd (14.4, 5.1, 1.9)	2.57, tdd (14.4, 5.1, 1.9)	4.33, t (3.0)	6.99, dt (9.8, 0.7)	1.35, td (12.2, 11.7, 2.8)[h]	2.59, ddd (15.2, 3.4, 2.1)
2*α*	2.60-2.48, m	1.69, tdd (14.0, 4.4, 2.8)	1.72-1.64, m	1.99, dq (13.9, 3.0)	6.21, dd (9.8, 0.7)	2.13, m[i]	-
2*β*	2.60-2.48, m	2.00, ddt (14.0, 5.1, 2,6)	2.06, ddt (14.2, 5.1, 2.4)	1.67, tt (13.9, 3.5)		2.05-1.97, m[i]	4.2, br s
3*α*	-	3.93, q (2.8)	5.06, q (2.8)	1.83, qd (13.4, 3.3)	-	1.31-1.22, m[j]	1.69, ddd (7.9, 3.0, 1.7, 2H)
3*β*	-	-	-	1.39, dq (13.4, 3.5)	-	1.73, m[j]	
4*α*	2.57, q (6.6)	1.55, qd (7.3, 2.8)	1.72-1.64, m	1.48, dqd (13.4, 6.9, 3.3)	2.61, q (6.8)	1.31-1.22, m	2.05–1.89, m
6*α*	2.45, br d (13.9)	2.08, br d (13.0)	2.09, br d (13.1)	2.12, br d (14.2)	2.38, br d (13.9)	1.77, dt (12.7, 2.3)[k]	2.05–1.89, m
6*β*	2.82, d (13.9)	2.87, d (13.0)	2.89, d (13.1)	2.90, d (14.2)	2.99, d (13.9)	1.16, td (12.7, 0.9)[k]	2.05–1.89, m
7*β*	-	-	-	-	-	2.21, m	3.13, dd (13.8, 5.1)
8*α*	-	-	-	-	-	2.05-1.97, m[l]	-
8*β*	-	-	-	-	-	1.87, dddd (16.9, 11.6, 3.4, 2.0)[l]	-
9	5.92, dd (1.8, 0.9)	5.77, d (1.9)	5.78, d (1.9)	5.84, s	6.07, t (0.7)	5.31, dt (5.6, 2.0)	5.81, d (2.0)
12	2.11, d (1.9)[c]	2.09, s[d]	2.10, s[e]	2.11, s[f]	2.18, d (2.2)[g]	1.74, t (1.1)	1.73, dd (1.5, 0.8)
13	1.86, d (1.2)[c]	1.83, d (1.0)[d]	1.83, d (1.0)[e]	1.85, s[f]	1.92, d (1.5)[g]	4.71, m	a: 4.97, p (1.5)
							b: 4.81, dq (1.8, 0.8)
14*β*	0.95, s	1.15, s	1.12, s	1.14, s	0.98, s	0.97, s	1.16, s
15*β*	1.08, d (6.7)	1.12, d (7.3)	1.02, d (7.1)	0.98, d (6.9)	1.17, d (6.8)	0.84, d (6.6)	0.92, d (6.9)
OCO<u>Me</u>	-	-	2.10, s	-	-	-	-

[a] 500 MHz; [b] 700 MHz; [c-l] Interchangeable assignments.

Table S2. ^{1}H NMR spectroscopic data for compounds **12-17** in $CDCl_3$.

	**12**	**13**	**14**	**15**	**16**	**17**
Position	**δ_H, Mult (*J* in Hz)**[b]	**δ_H, Mult (*J* in Hz)**[a]	**δ_H, Mult (*J* in Hz)**[c]	**δ_H, Mult (*J* in Hz)**[b]	**δ_H, Mult (*J* in Hz)**[b]	**δ_H, Mult (*J* in Hz)**[b]
1α	4.56, dd (10.1, 2.0)	6.31-6.23, m	3.76, d (3.6)	3.63, d (3.5)	3.63, d (3.5)	3.15, dd (4.0, 1.2)
1β	-		-	-	-	-
2α	-	6.31-6.23, m	3.87, dd (5.1, 3.6)	3.89, dd (5.0, 3.5)	3.95, dd (5.1, 3.5)	3.45, dd (5.0, 4.0)
2β	3.50, dd (10.1. 3.3)		-	-	-	-
3α	-	4.18, q (4.6)	4.11, dt (9.9, 5.1)	5.13, t (5.0)	5.13, t (5.1)	5.23, dd (5.9, 5.0)
3β	3.99, t (3.0)	-	-	-	-	-
4α	1.63-1.46, m	1.76, td (7.2, 4.6)	1.54, m	1.77, m	1.77, qd (7.1, 5.1)	1.64, p (7.1)
6α	2.00, dd (13.0, 4.6)[d]	2.01, dd (12.9, 4.9)[e]	2.09, dd (14.7, 1.0)	2.12, d (14.7)	2.14, d (14.2)	1.78, dd (13.2, 1.0)
6β	1.86, q (13.7, 12.3)[d]	1.85, dd (13.9, 12.9)[e]	1.92, d (14.7)	1.92, d (14.7)	1.82, d (14.2)	1.38, d (13.2)
7β	3.15, dd (14.4, 4.5)	3.24, dd (13.9, 4.9)	-	-	-	-
9α	6.22, d (2.0)	5.79, s	6.39, s	6.38, s	6.41, s	1.90, dd (12.6, 2.1)
9β						2.29, q (12.6)
10α	-	-	-	-	-	2.23, ddd (12.6, 2.1, 1.2)
12	1.73, s	1.72, dd (1.5, 0.8)	1.38, s	1.39, s	9.70, s	3.82, m
13	a: 4.98, m<br>b: 4.82, s	a: 4.98, p (1.5)<br>b: 4.86, dt (1.8, 0.8)	1.52, s	1.52, s	1.47, s	1.44, s
14β	1.40, s	1.32, s	1.28, s	1.30, s	1.43, s	1.15, s
15β	1.17, d (7.1)	1.15, d (7.2)	1.11, d (7.1)	1.00, d (7.1)	1.01, d (7.1)	0.90, d (7.1)
OCO<u>Me</u>	-	-	-	2.14, s	2.15, s	2.11, s

[a] 400 MHz; [b] 500 MHz; [c] 600 MHz; [d-e] Interchangeable assignments.

Table S3. ^{13}C NMR spectroscopic data for compounds **5-11** in $CDCl_3$.

	**5**	**6**	**7**	**8**	**9**	**10**	**11**
Position	***δ*C, Type**[a]	***δ*C, Type**[a]	***δ*C, Type**[b]	***δ*C, Type**[a]	***δ*C, Type**[a]	***δ*C, Type**[a]	***δ*C, Type**[a]
1	31.1, CH_2	26.7, CH_2	27.1, CH_2	72.7, CH	142.0, CH	31.1, CH_2	40.0, CH_2
2	39.8, CH_2	33.7, CH_2	30.5, CH_2	32.8, CH_2	131.8, CH	32.6, CH_2	66.8, CH
3	210.2, C	71.3, CH	73.3, CH	24.8, CH_2	200.0, C	27.8, CH_2	37.1, CH_2
4	52.6, CH	45.4, CH	44.2, CH	42.3, CH	51.4, CH	44.2, CH	36.3, CH
5	44.6, C	41.4, C	41.3, C	41.0, C	41.4, C	38.7, C	39.3, C
6	40.7, CH_2	41.9, CH_2	41.7, CH_2	42.3, CH_2	40.0, CH_2	43.3, CH_2	41.2, CH_2
7	126.7, C	127.9, C	127.6, C	128.1, C	126.3, C	37.8, CH	51.0, CH
8	191.0, C	192.2, C	191.8, C	192.4, C	190.6, C	31.3, CH_2	198.2, C
9	127.7, CH	126.1, CH	126.6, CH	129.1, CH	131.6, CH	118.8 CH	127.0, CH
10	162.8, C	168.3, C	166.7, C	165.6, C	156.6, C	144.4, C	166.2, C
11	144.6, C	143.1, C	143.6, C	144.3, C	148.4, C	150.6, C	143.6, C
12	22.3,[c] CH_3	22.6,[d] CH_3	22.7,[e] CH_3	22.8,[f] CH_3	23.4,[g] CH_3	20.8, CH_3	20.0, CH_3
13	22.7,[c] CH_3	22.1,[d] CH_3	22.2,[e] CH_3	22.4,[f] CH_3	23.0,[g] CH_3	108.3, CH_2	114.2, CH_2
14	18.5, CH_3	18.9, CH_3	18.4, CH_3	18.1, CH_3	18.1, CH_3	18.1, CH_3	15.4, CH_3
15	7.5, CH_3	12.1, CH_3	11.8, CH_3	15.5, CH_3	7.1, CH_3	15.7, CH_3	14.7, CH_3
OCOMe	-	-	170.6, C	-	-	-	-
OCOMe	-	-	21.2, CH_3	-	-	-	-

[a] 125 MHz; [b] 175 MHz; [c-g] Interchangeable assignments.

Table S4. ^{13}C NMR spectroscopic data for compounds **12-17** in $CDCl_3$.

	**12**	**13**	**14**	**15**	**16**	**17**
Position	***δ*C, Type**[b]	***δ*C, Type**[a]	***δ*C, Type**[c]	***δ*C, Type**[b]	***δ*C, Type**[b]	***δ*C, Type**[b]
1	69.5, CH	129.3, CH	58.0, CH	55.9, CH	55.9, CH	57.5, CH
2	76.2, CH	136.1, CH	58.0, CH	55.1, CH	55.5, CH	50.2, CH
3	73.9, CH	68.2, CH	67.7, CH	69.9, CH	69.8, CH	71.3, CH
4	44.4, CH	42.0, CH	44.0, CH	42.0, CH	42.8, CH	44.0, CH
5	39.5, C	36.2, C	37.1, C	37.2, C	38.1, C	38.4, C
6	42.9, CH_2	40.4, CH_2	42.1, CH_2	41.8, CH_2	41.6, CH_2	35.6, CH_2
7	50.1, CH	51.4, CH	62.3, C	62.4, C	67.3, C	68.3, C
8	198.5, C	199.0, C	194.0, C	193.9, C	191.6, C	102.0, C
9	121.5, CH	125.5, CH	131.9, CH	131.4, CH	129.9, CH	34.4, CH_2
10	166.5, C	161.6, C	160.5, C	160.7, C	164.5, C	41.7, CH
11	143.3, C	143.7, C	64.6, C	64.6, C	67.4, C	61.1, C
12	20.1, CH_3	19.8, CH_3	21.5, CH_3	21.6, CH_3	198.6, C	69.9, CH_2
13	114.4, CH_2	114.7, CH_2	19.4, CH_3	19.5, CH_3	13.7, CH_3	11.3, CH_3
14	19.5, CH_3	18.9, CH_3	23.4, CH_3	22.9, CH_3	21.9, CH_3	15.5, CH_3
15	11.3, CH_3	10.4, CH_3	10.6, CH_3	10.4, CH_3	10.1, CH_3	9.7, CH_3
O<u>C</u>OMe	-	-	-	170.7, C	170.6, C	171.0, C
OCO<u>Me</u>	-	-	-	20.7, CH_3	20.7, CH_3	20.9, CH_3

[a] 100 MHz; [b] 125 MHz; [c] 150 MHz.

Table S5. ^{1}H and ^{13}C NMR (500 and 125 MHz) spectroscopic data for compounds **18-19** in $CDCl_3$.

	**18**		**19**	
Position	**δ_H, Mult (*J* in Hz)**	**δ_C, Type**	**δ_H, Mult (*J* in Hz)**	**δ_C, Type**
1	-	170.0,[b] C	-	169.7,[d] C
3a	3.57, m, 2H	45.5, CH_2	3.62, m	45.1, CH_2
3b			3.53, ddd (12.0, 9.1, 3.0)	
4a	2.18-1.97, m	22.7, CH_2	2.09-1.99, m	22.4, CH_2
4b	1.90, m		1.90, m	
5a	2.35, dtd (13.3, 6.9, 3.0)	28.1, CH_2	2.37, m	28.5, CH_2
5b	2.18-1.97, m		2.09-1.99, m	
6α	4.11, t (8.2)	59.0, CH	4.06, m	58.8, CH
7	-	166.1,[b] C	-	164.9,[d] C
8	5.65, br s	-	5.63, br s	-
9α	4.01, dd (9.7, 3.9)	53.4, CH	3.96, br t (2.5)	60.5, CH
10a	2.18-1.97, m	38.6, CH_2	2.31, m	35.2, CH
10b	1.51, m		-	
11a	1.72, m	24.8, CH	1.42, dqd (13.3, 7.5, 3.8)	24.0, CH_2
11b	-		1.16, m	
12	1.00, d (6.6)[a]	23.3,[c] CH_3	0.92, t (7.5)	12.0, CH_3
Me-10	-	-	1.04, d (7.3)	16.0, CH_3
Me-11	0.95, d (6.6)[a]	21.2,[c] CH_3	-	-

[a-d] Interchangeable assignments.

Table S6. Optical activities for compounds **5-8** and **10-19**.

Compound	$[\alpha]_D^T$
**5**	+31 (c 0.06, $CHCl_3$, 26ºC)
**6**	+110 (c 0.11, $CHCl_3$, 21ºC)
**7**	+34 (c 0.21, $CHCl_3$, 26ºC)
**8**	+47 (c 0.05, $CHCl_3$, 22ºC)
**10**	+89 (c 0.04, Hexane, 24ºC)
	+15 (c 0.23, $CHCl_3$, 24ºC)
**11**	+90 (c 0.12, $CHCl_3$, 24ºC)
**12**	+25 (c 0.05, MeOH, 22ºC)
**13**	+377 (c 0.25, $CHCl_3$, 22ºC)
**14**	+71 (c 0.06, $CHCl_3$, 23ºC)
**15**	+137 (c 0.07, $CHCl_3$, 23ºC)
**16**	+239 (c 0.10, $CHCl_3$, 24ºC)
**17**	+78 (c 0.11, $CHCl_3$, 25ºC)
**18**	-89 (c 0.3, $CHCl_3$, 27ºC)
**19**	-92 (c 0.4, $CHCl_3$, 27ºC)
	-66 (c 0.4, EtOH, 26ºC)

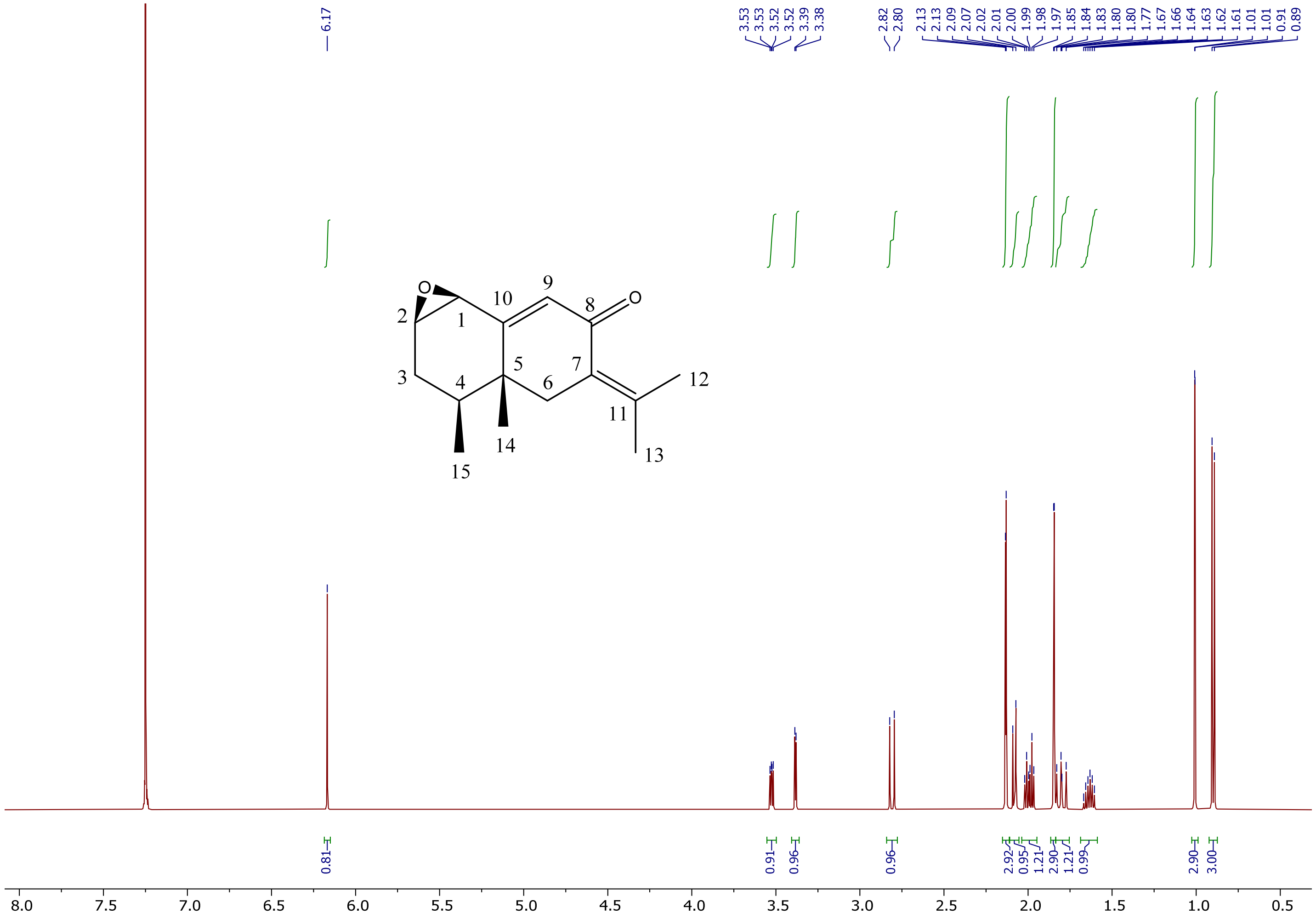

Figure S1. ^{1}H NMR spectrum (500 MHz, $CDCl_3$) of compound **1**.

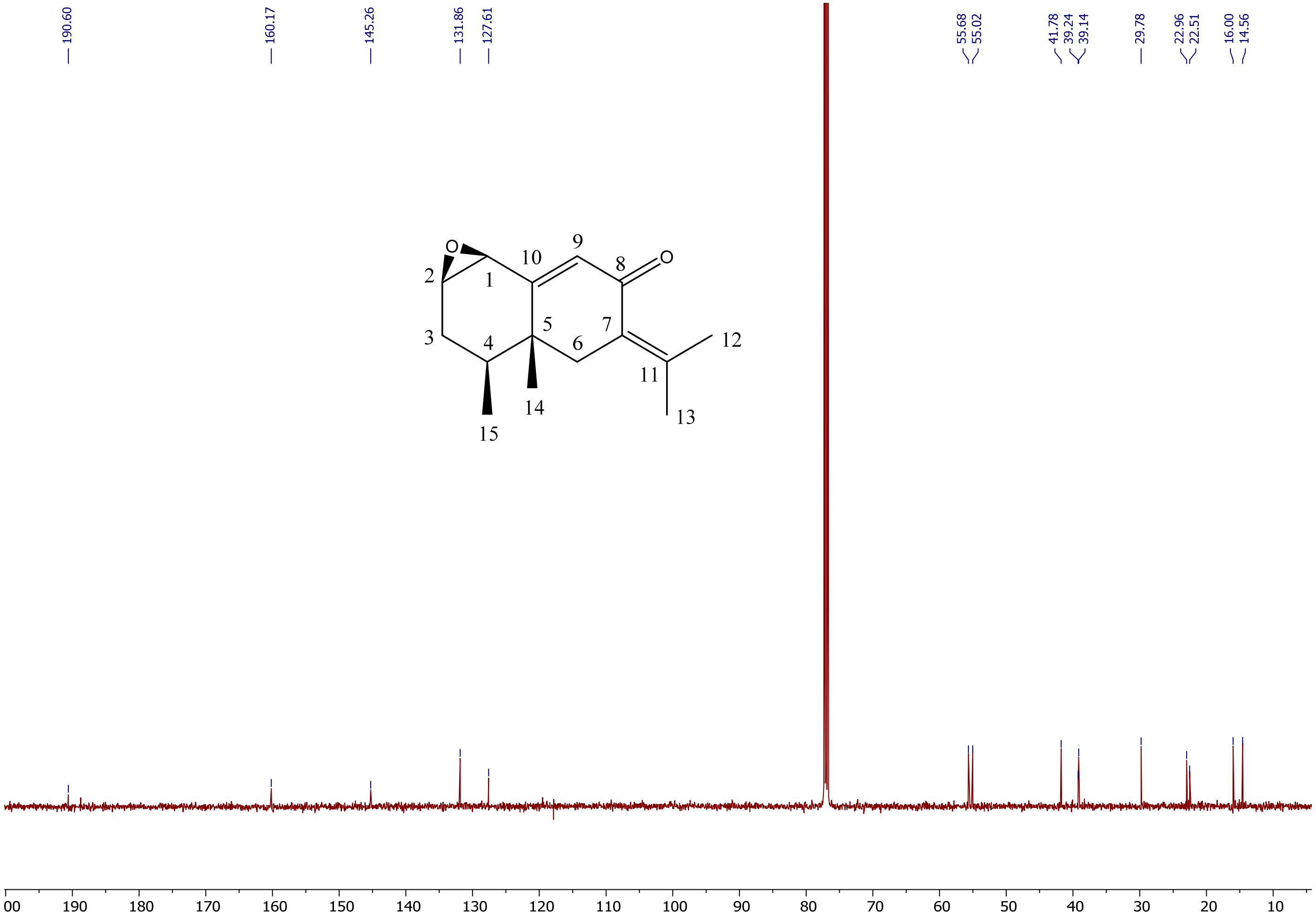

Figure S2. ^{13}C NMR spectrum (125 MHz, $CDCl_3$) of compound **1**.

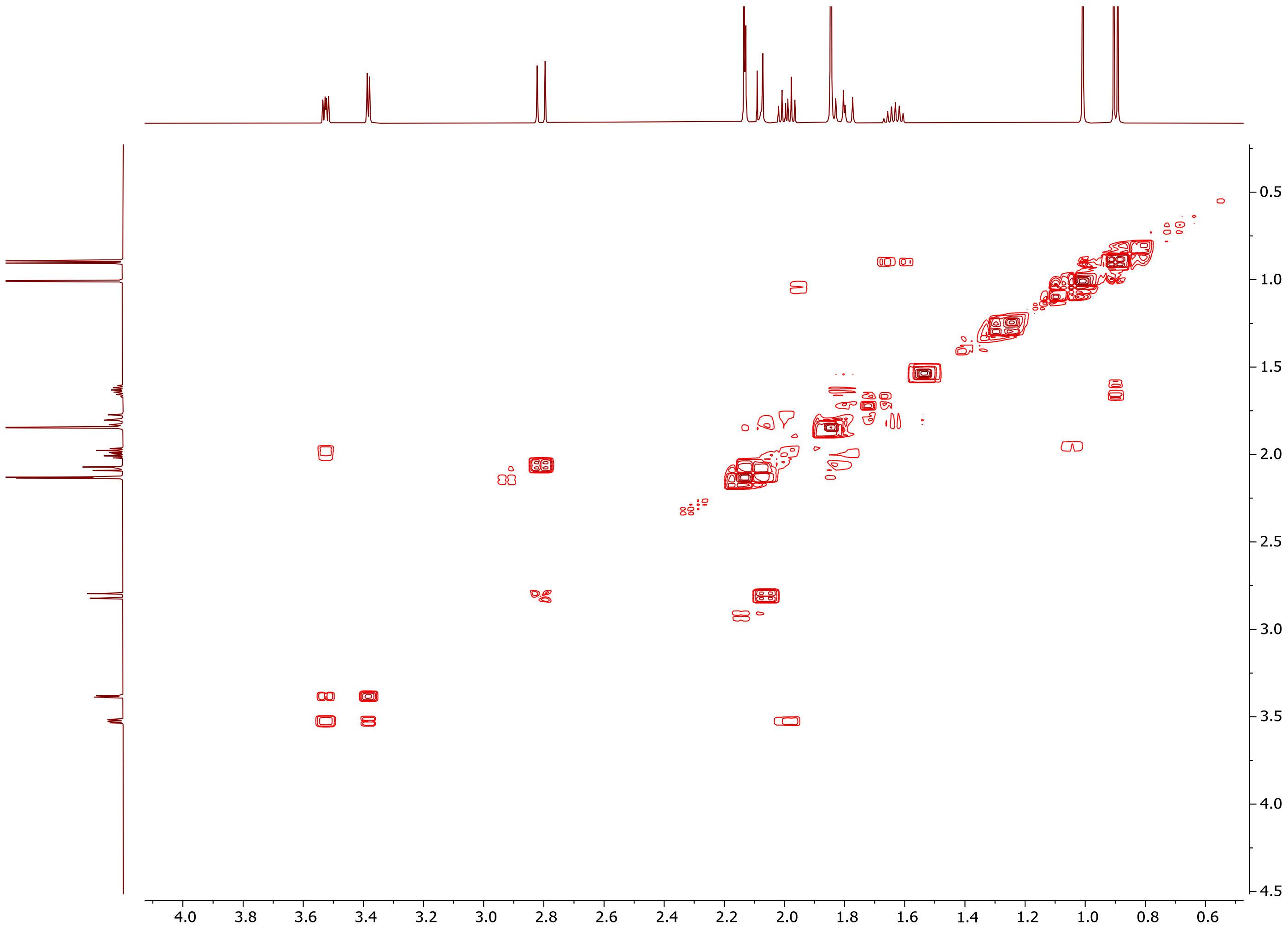

Figure S3. gCOSY spectrum of compound **1**.

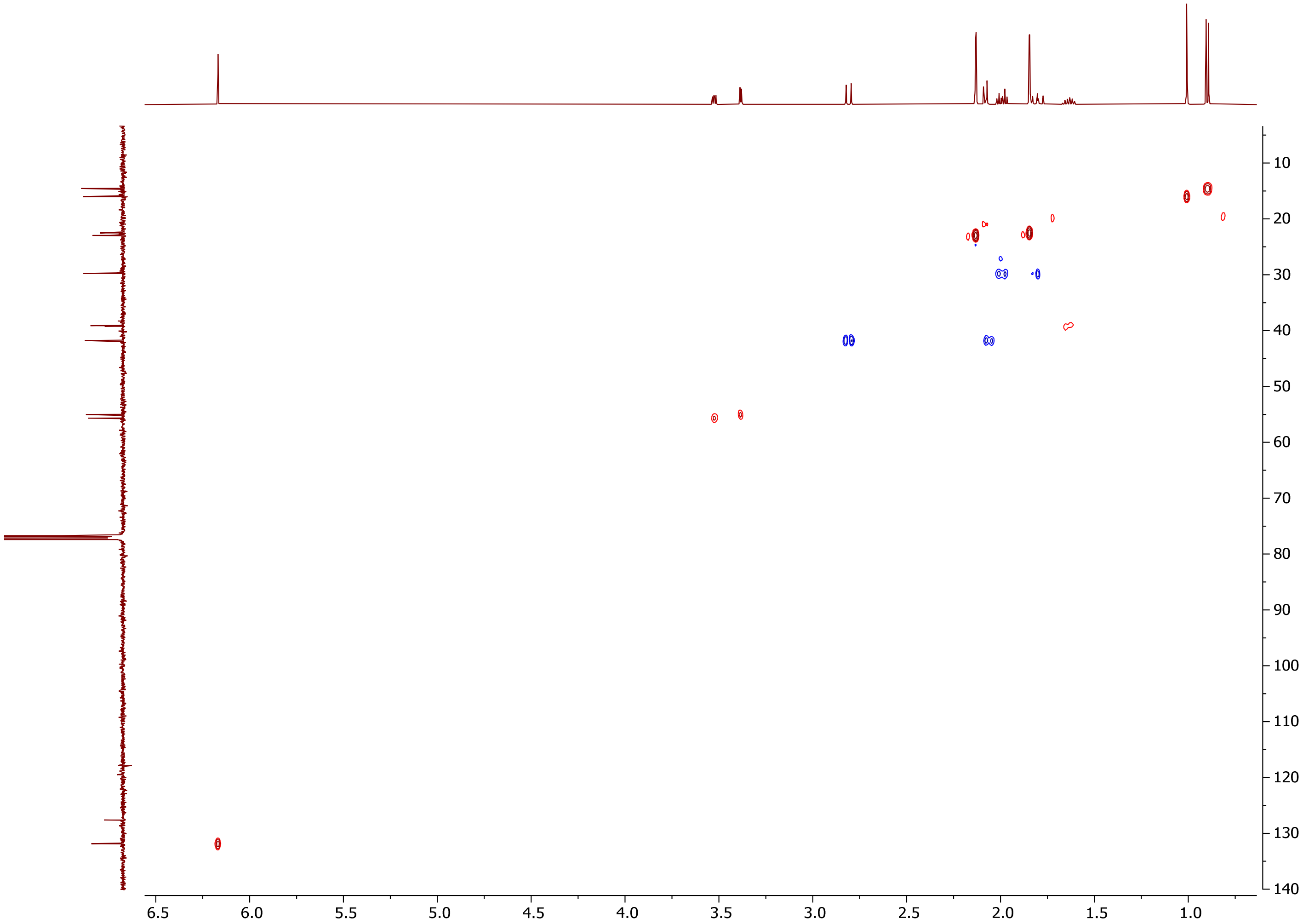

Figure S4. gHSQC spectrum of compound **1**.

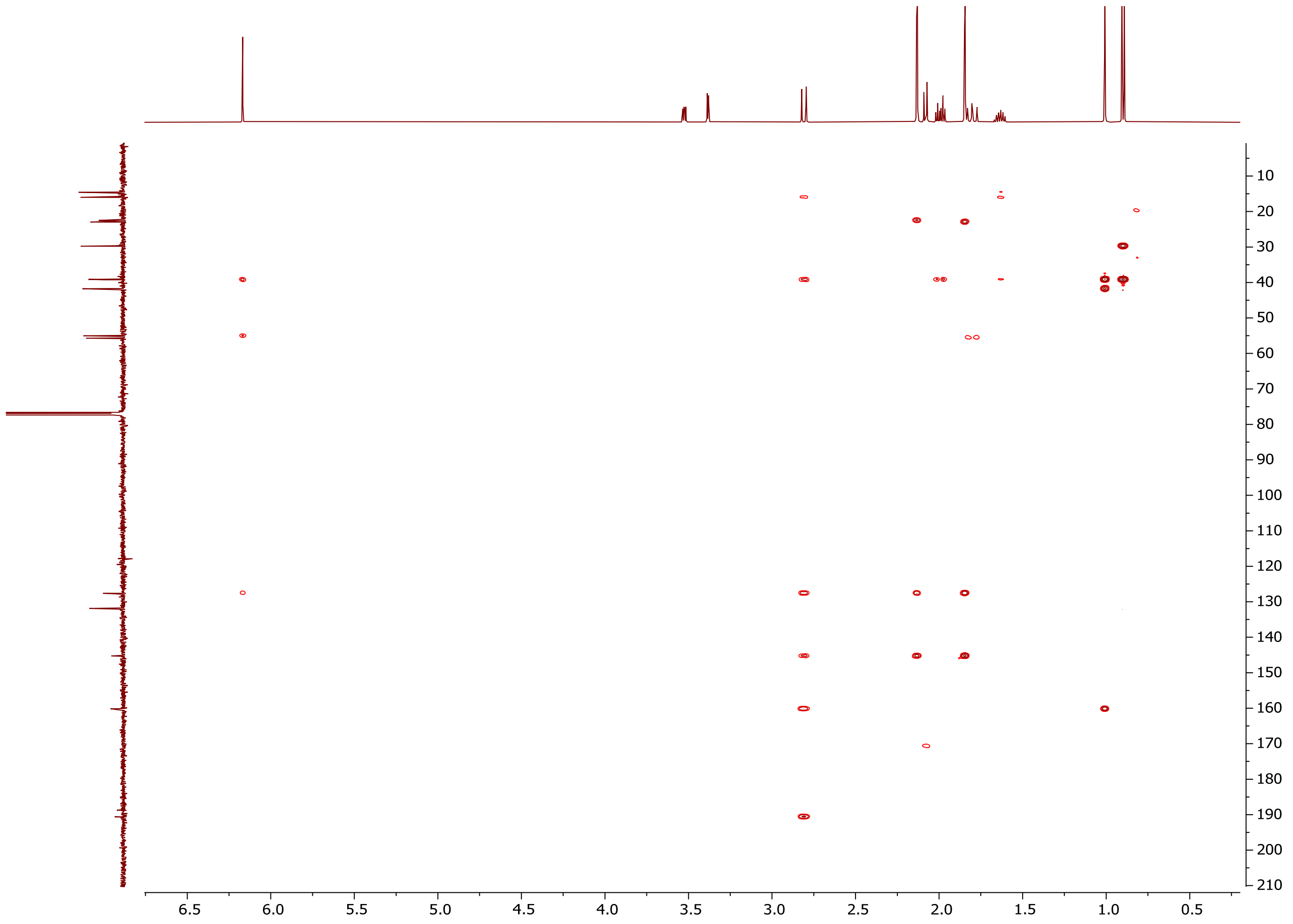

Figure S5. gHMBC spectrum of compound **1**.

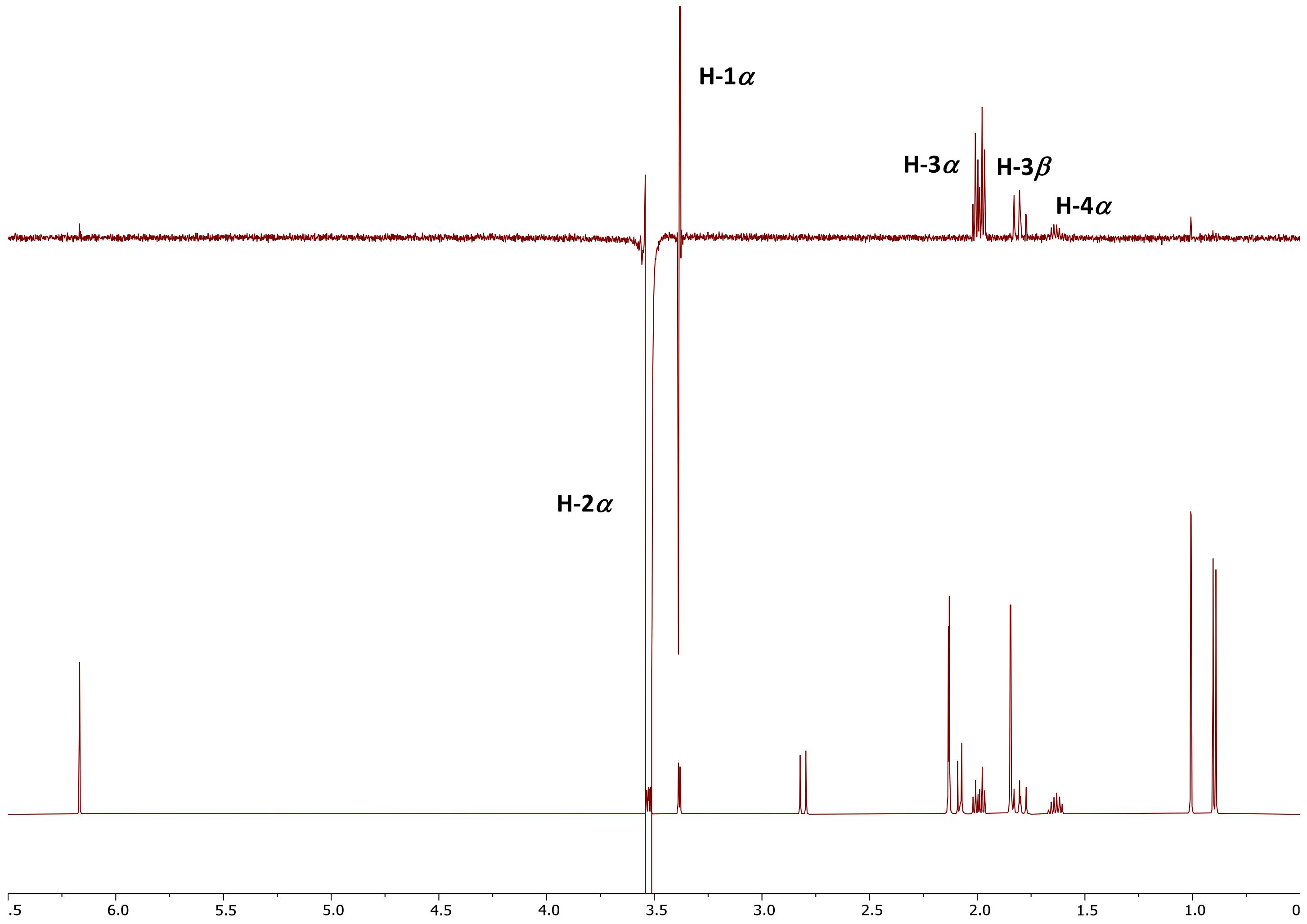

Figure S6a. 1D NOESY spectrum of compound **1**.

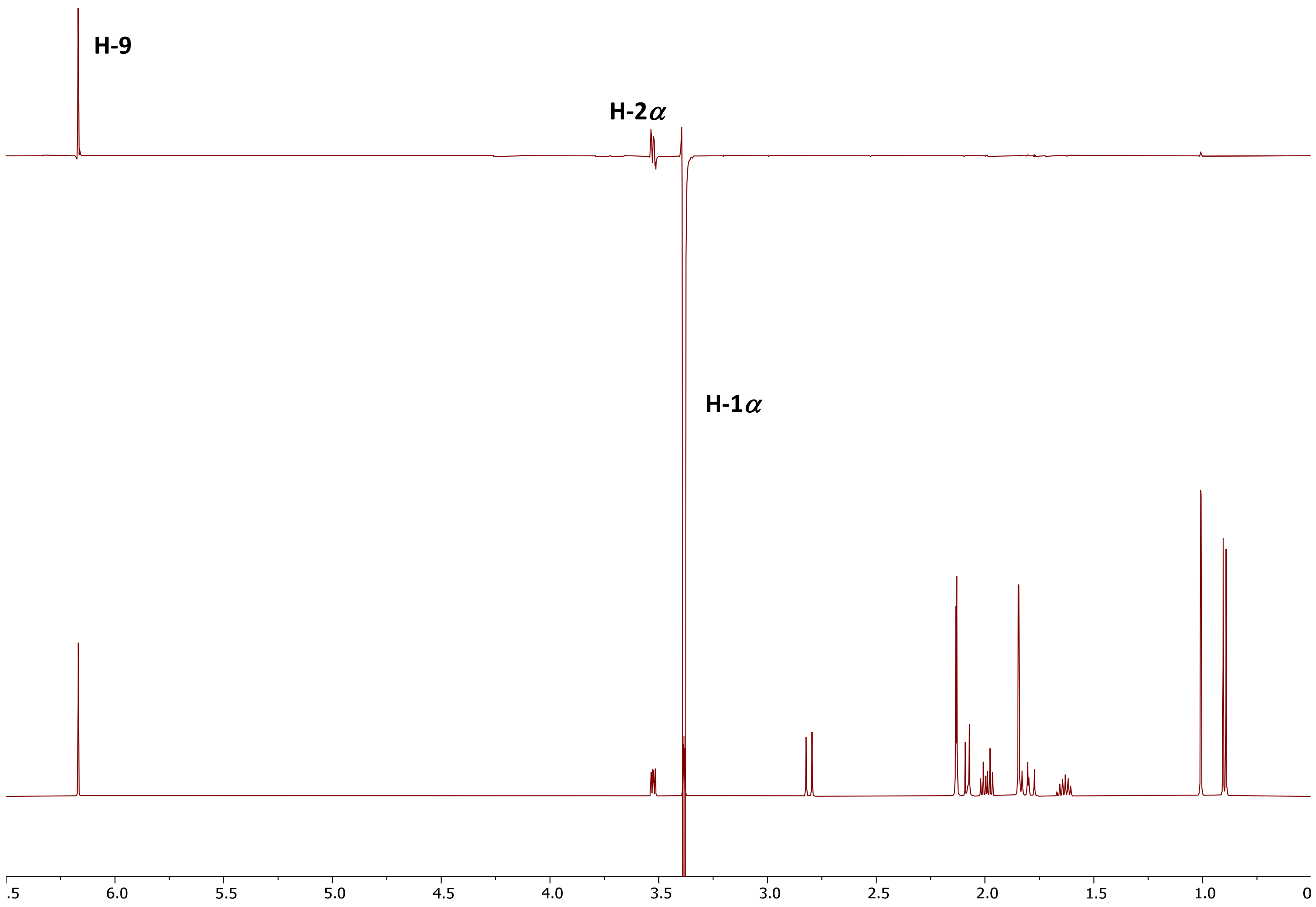

Figure S6b. 1D NOESY spectrum of compound **1**.

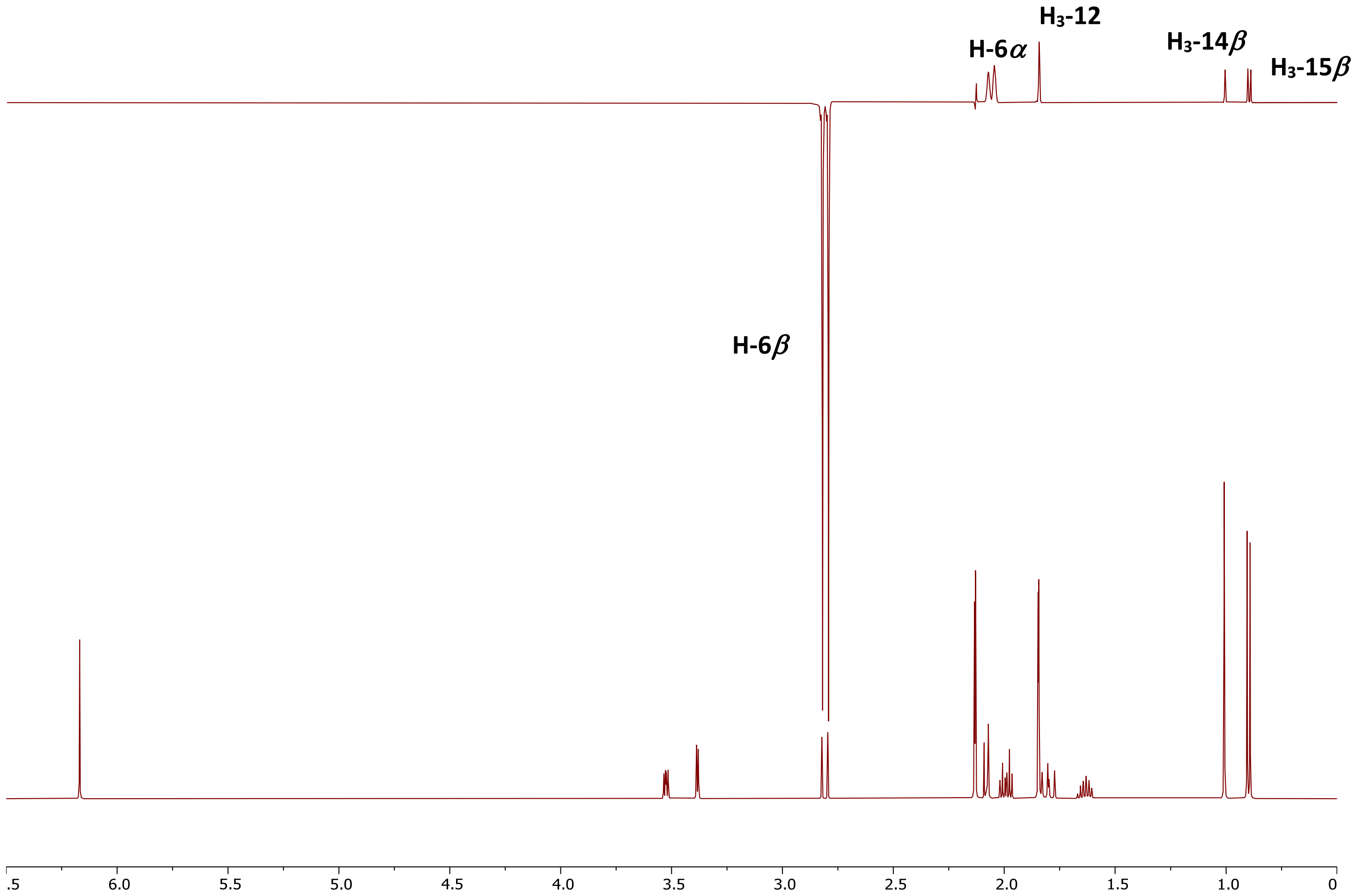

Figure S6c. 1D NOESY spectrum of compound **1**.

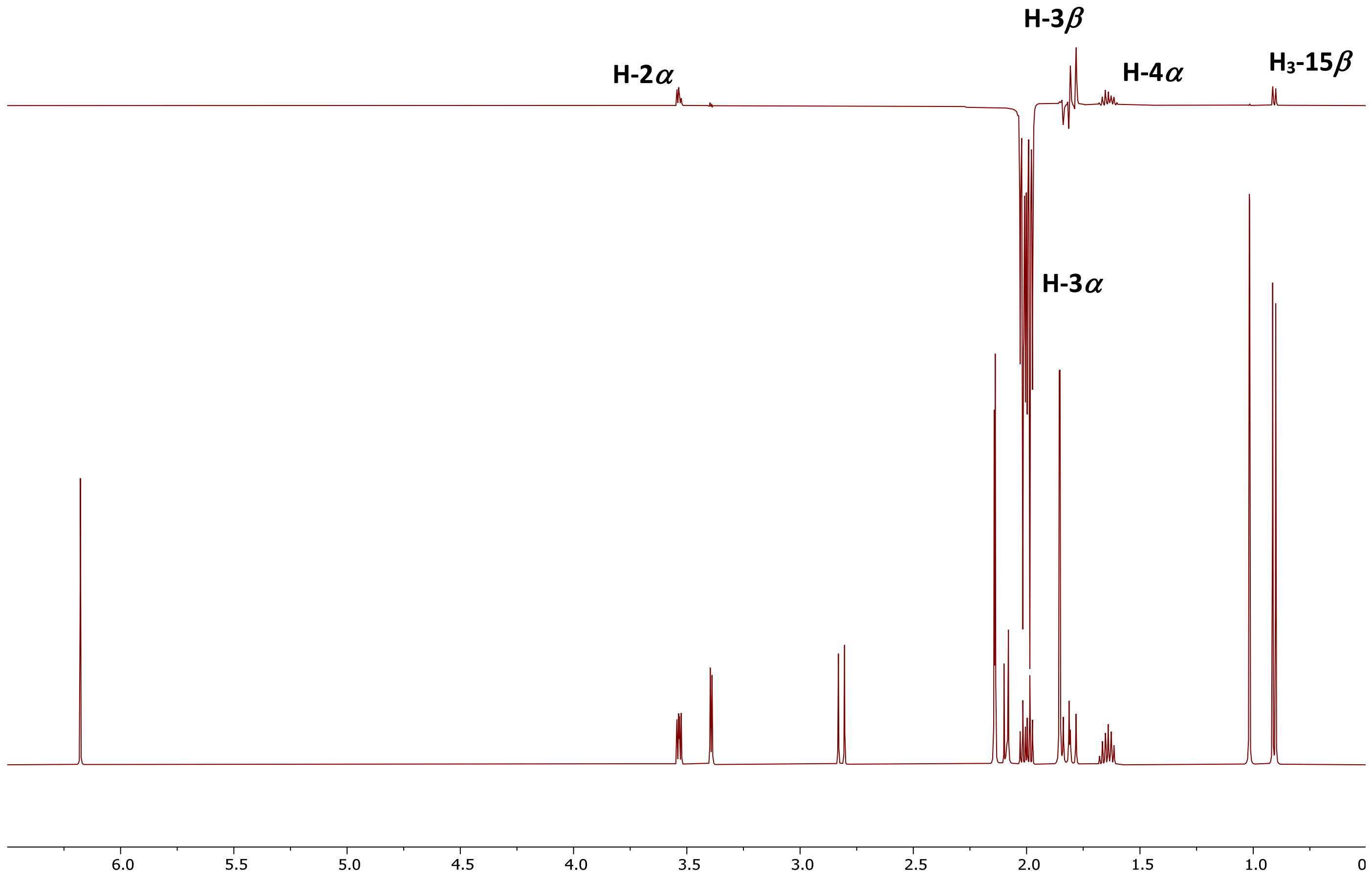

Figure S6d. 1D NOESY spectrum of compound **1**.

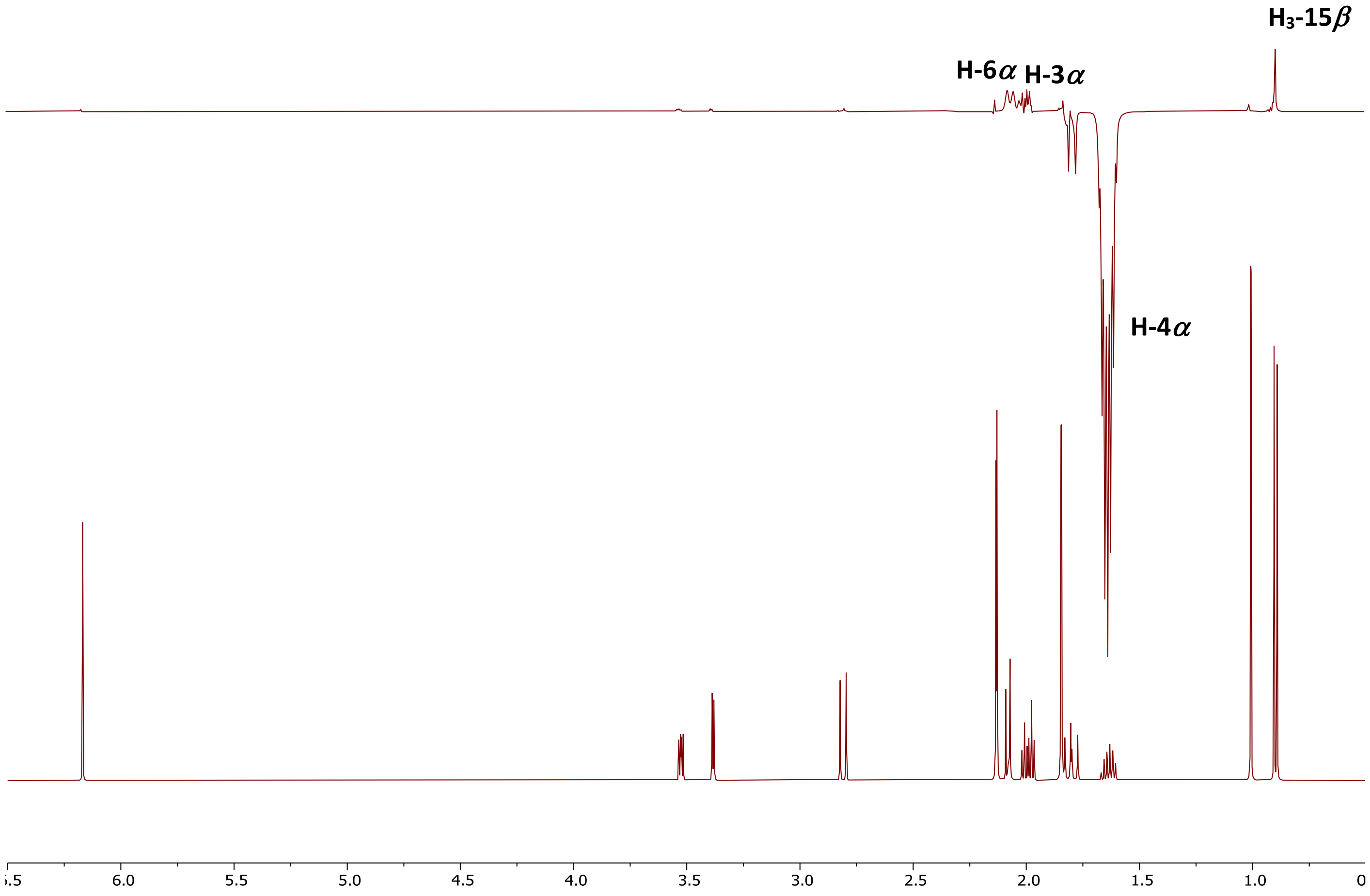

Figure S6e. 1D NOESY spectrum of compound **1**.

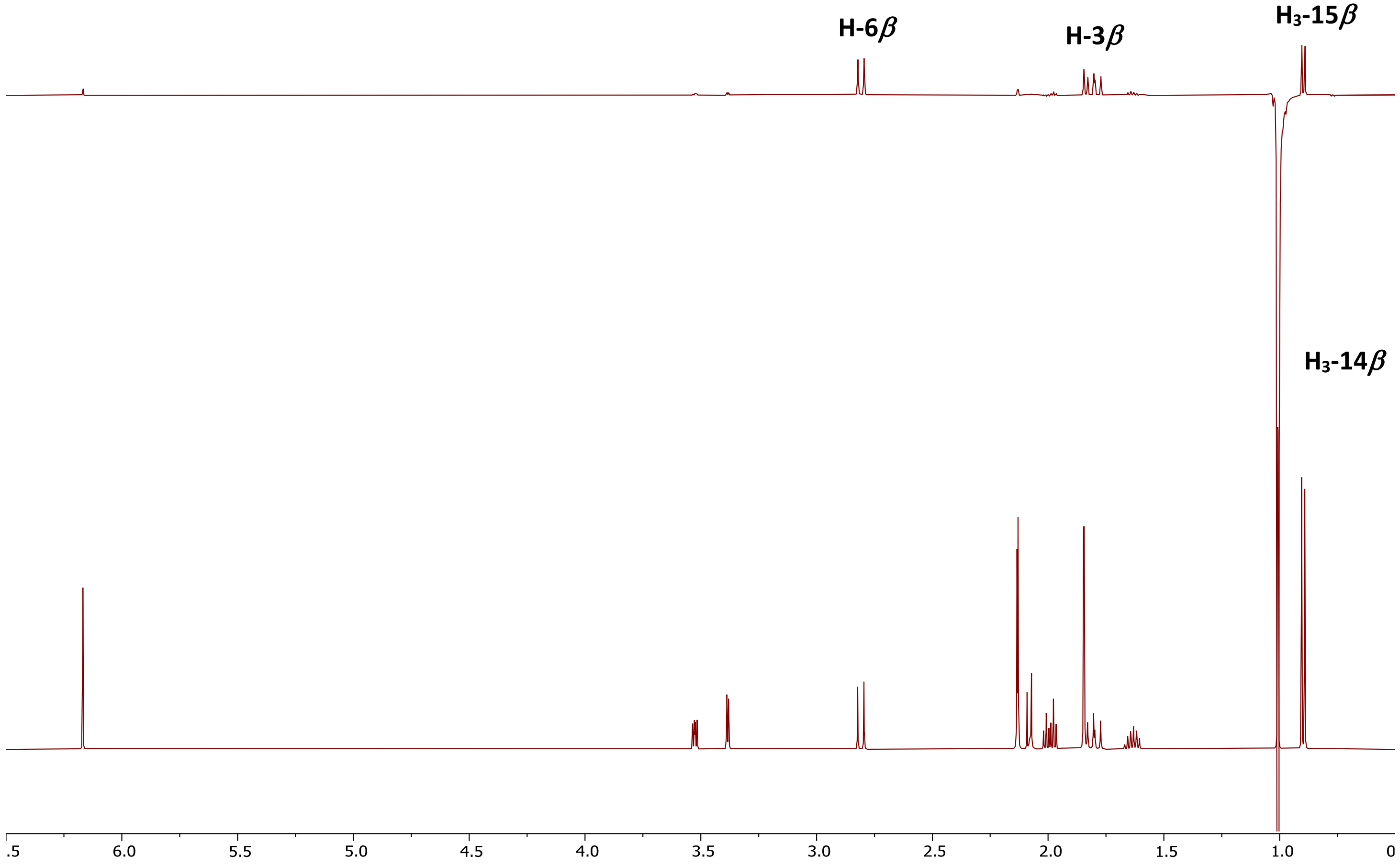

Figure S6f. 1D NOESY spectrum of compound **1**.

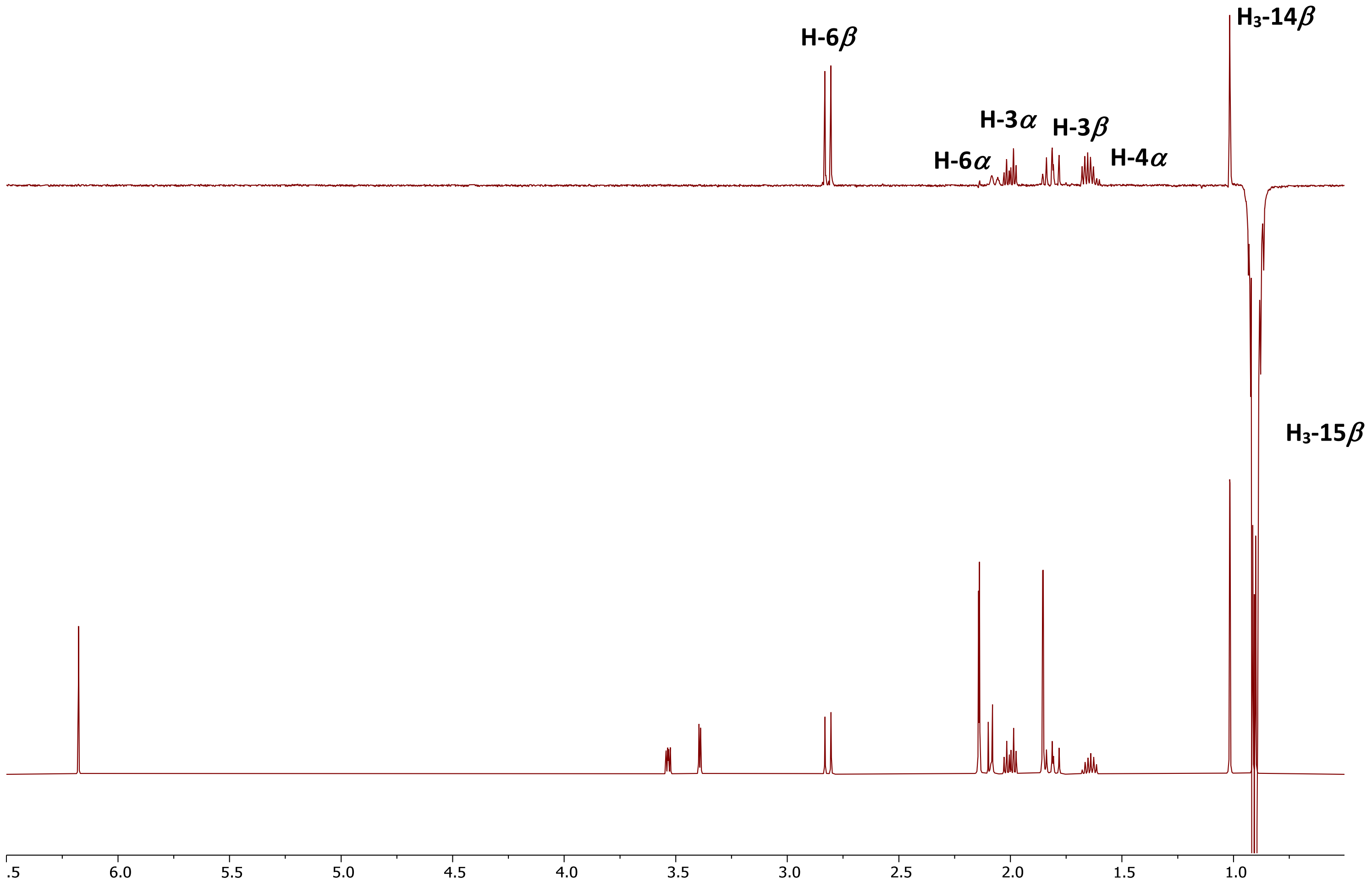

Figure S6g. 1D NOESY spectrum of compound **1**.

Single Mass Analysis

Tolerance = 5.0 mDa / DBE: min = -1.5, max = 80.0
Element prediction: Off
Number of isotope peaks used for i-FIT = 3

Monoisotopic Mass, Even Electron Ions
65 formula(e) evaluated with 2 results within limits (up to 5 closest results for each mass)
Elements Used:
C: 0-100 H: 0-100 O: 0-20 Na: 0-1
ROSA DURAN
CARLOS-4-F30 371 (4.271)

1: TOF MS ES+
1.50e+006

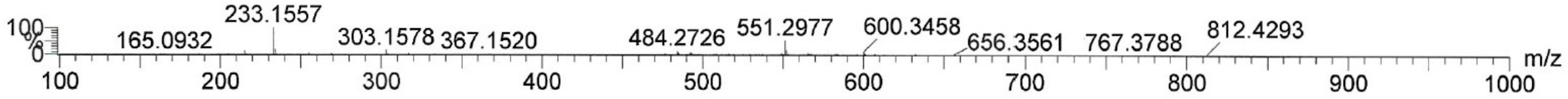

Mass	Calc. Mass	mDa	PPM	DBE	i-FIT	Norm	Conf(%)	Formula
Minimum:				-1.5				
Maximum:		5.0	5.0	80.0				
233.1557	233.1542	1.5	6.4	5.5	802.2	0.032	96.84	C15 H21 O2
	233.1517	4.0	17.2	2.5	805.6	3.453	3.16	C13 H22 O2 Na

Figure S7. HRESIMS spectrum of compound **1**.

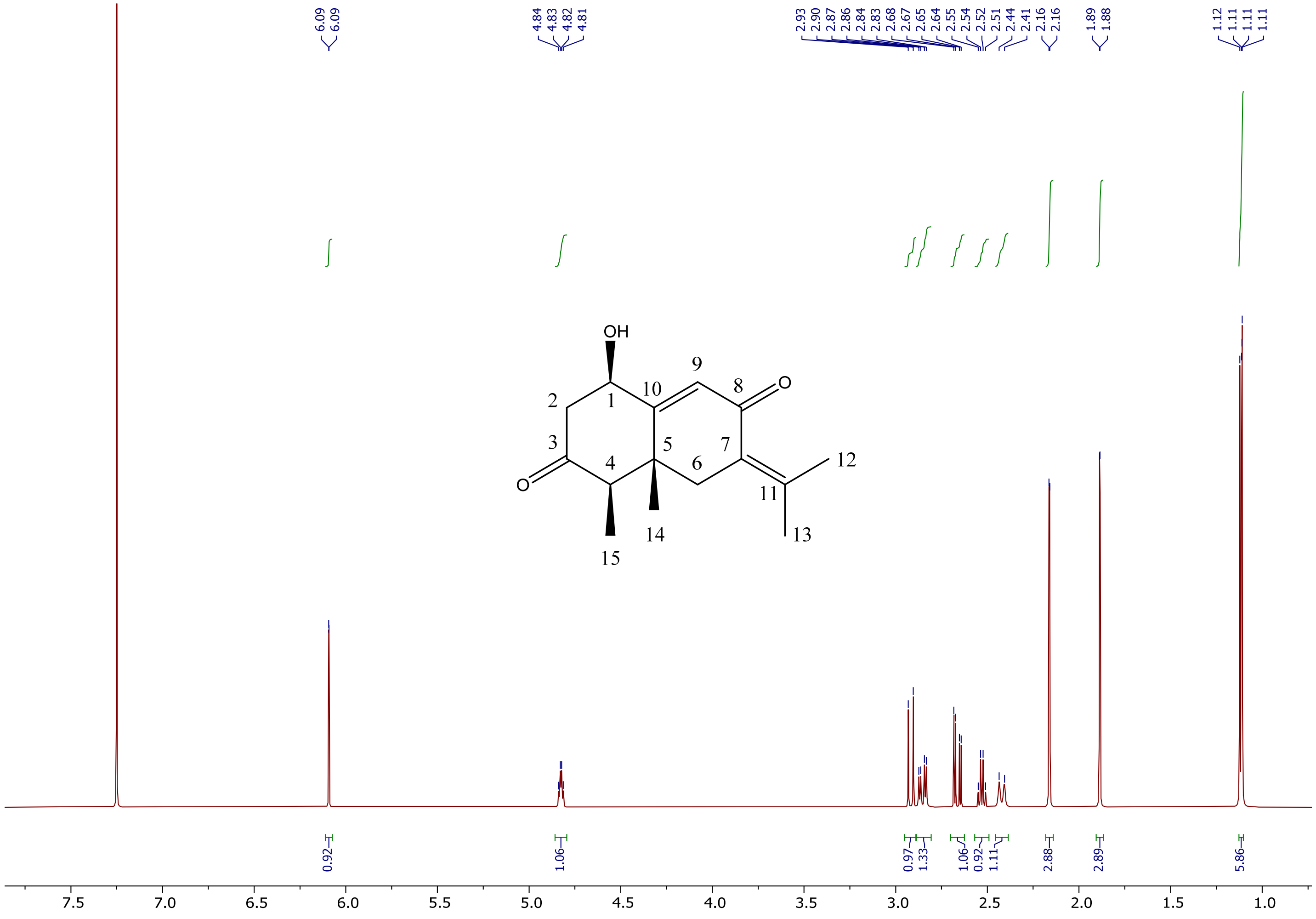

Figure S8. ^{1}H NMR spectrum (500 MHz, $CDCl_3$) of compound **2**.

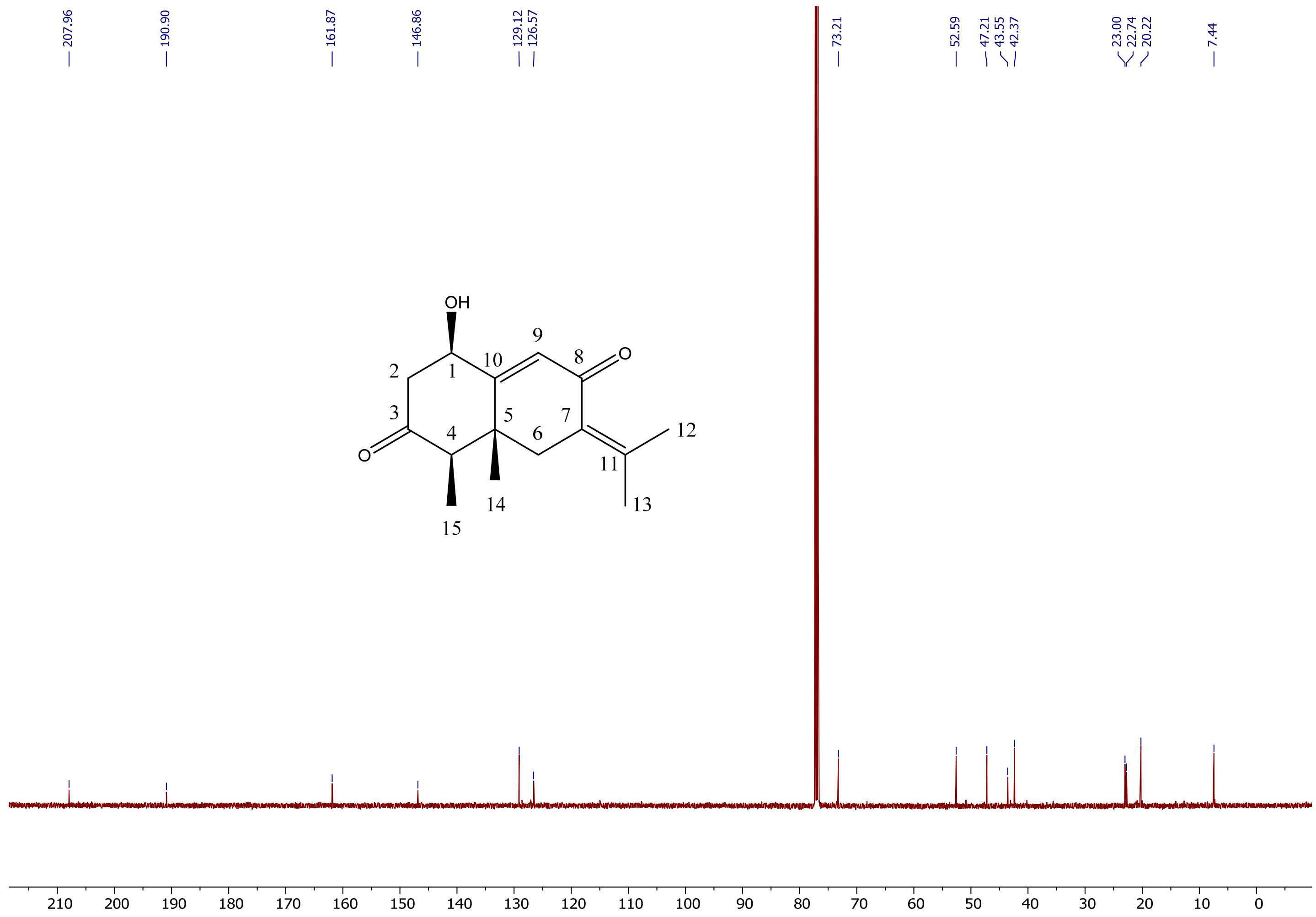

Figure S9. ^{13}C NMR spectrum (125 MHz, $CDCl_3$) of compound **2**.

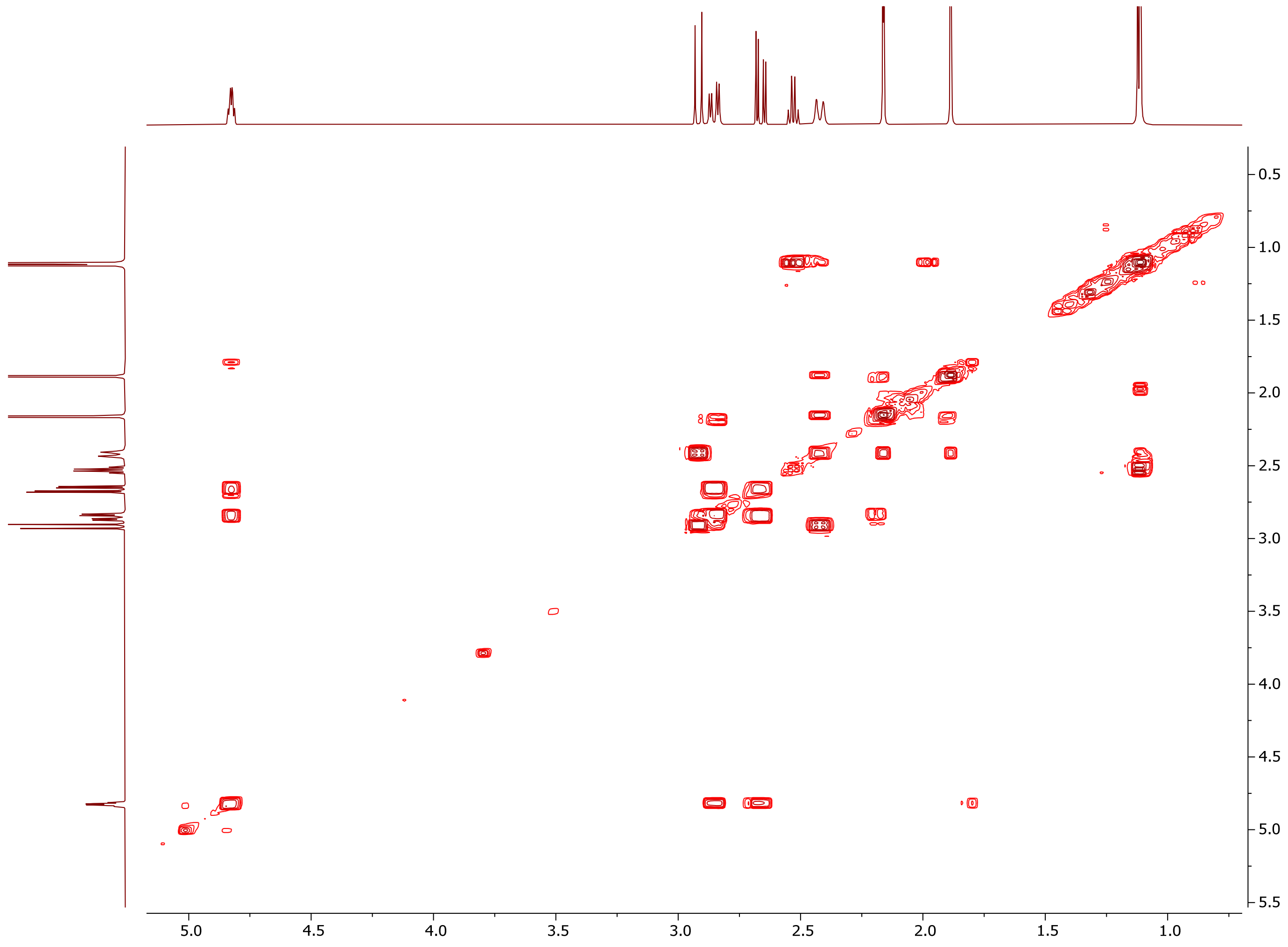

Figure S10. gCOSY spectrum of compound **2**.

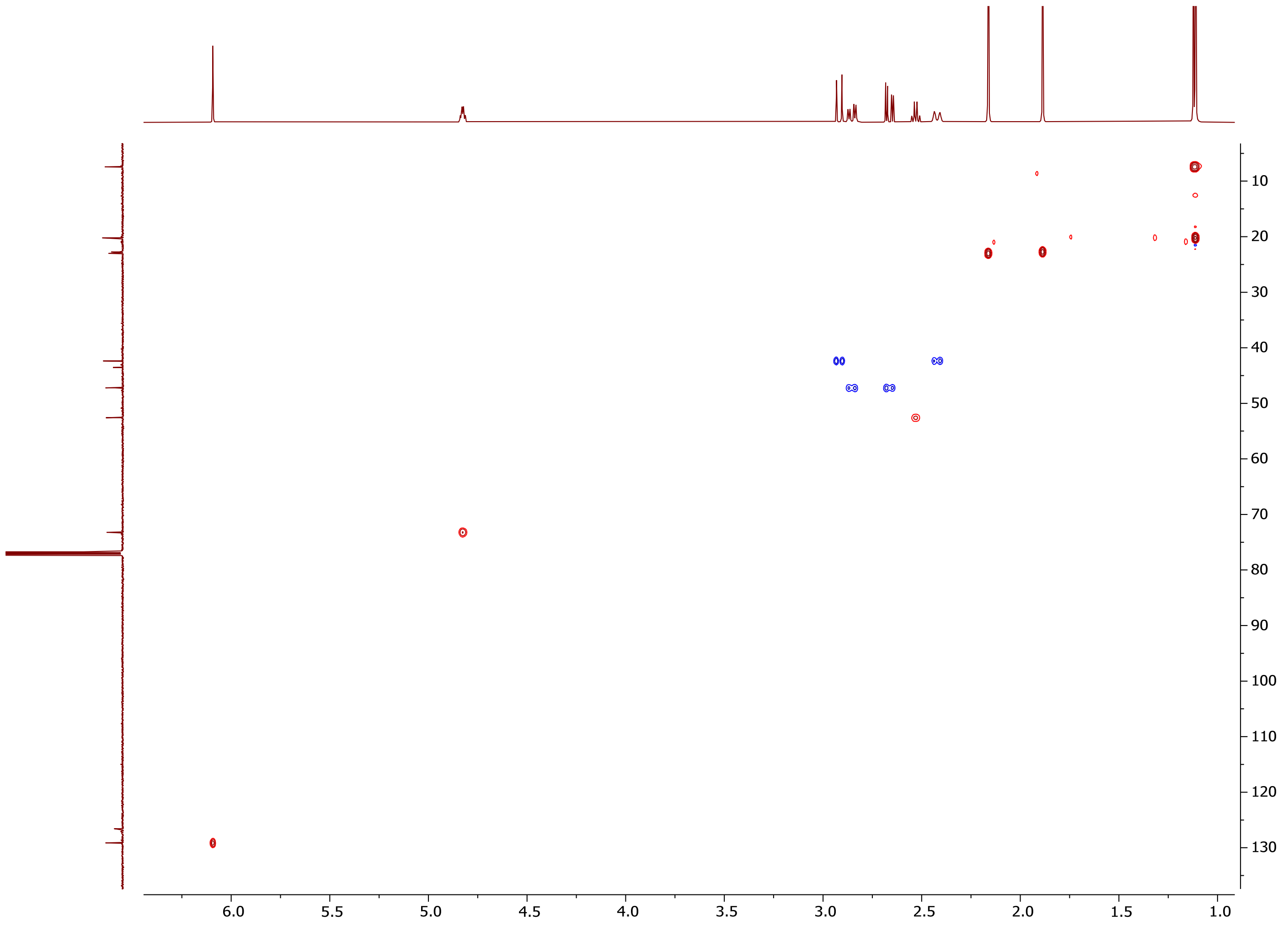

Figure S11. gHSQC spectrum of compound **2**.

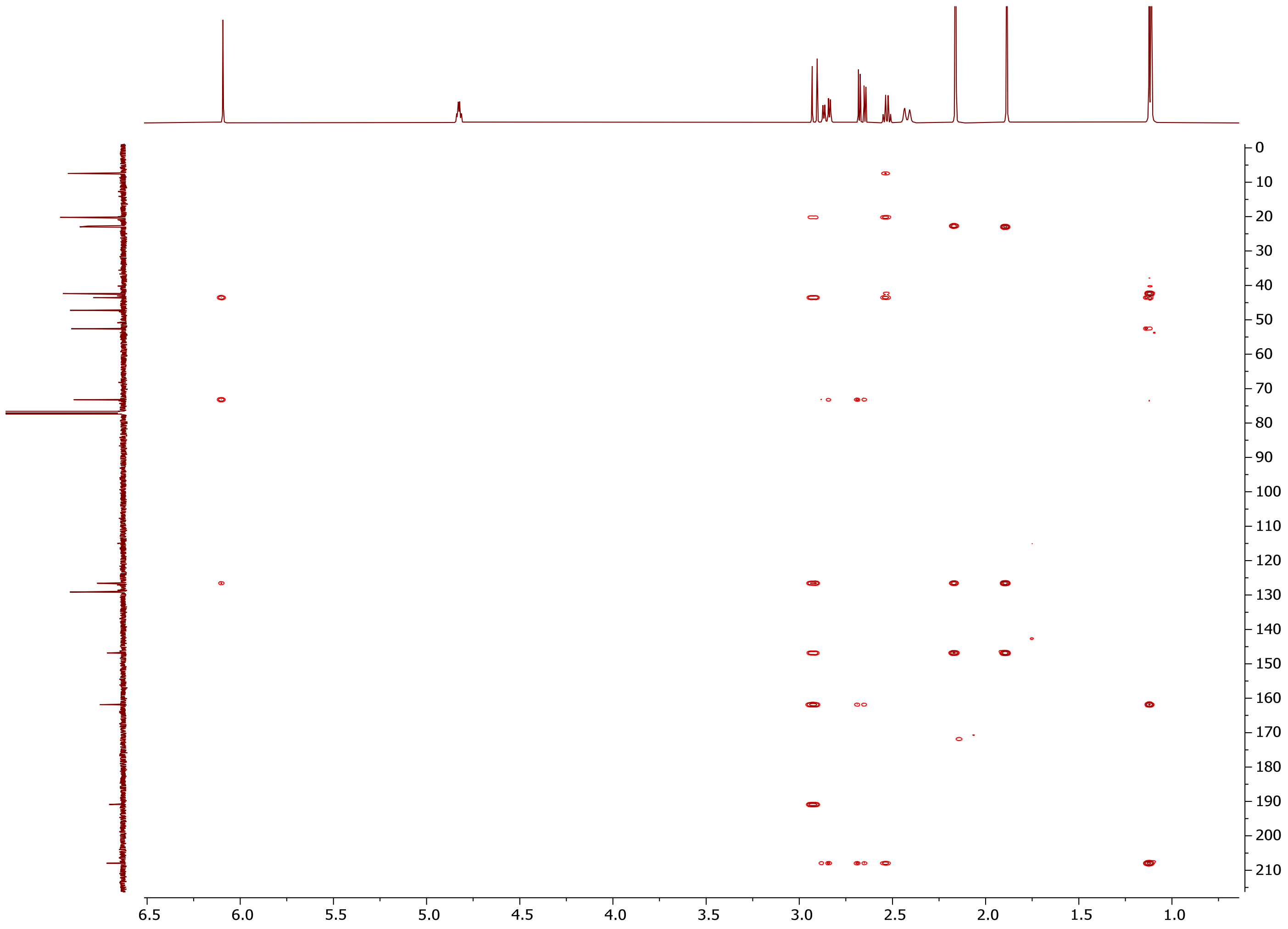

Figure S12. gHMBC spectrum of compound **2**.

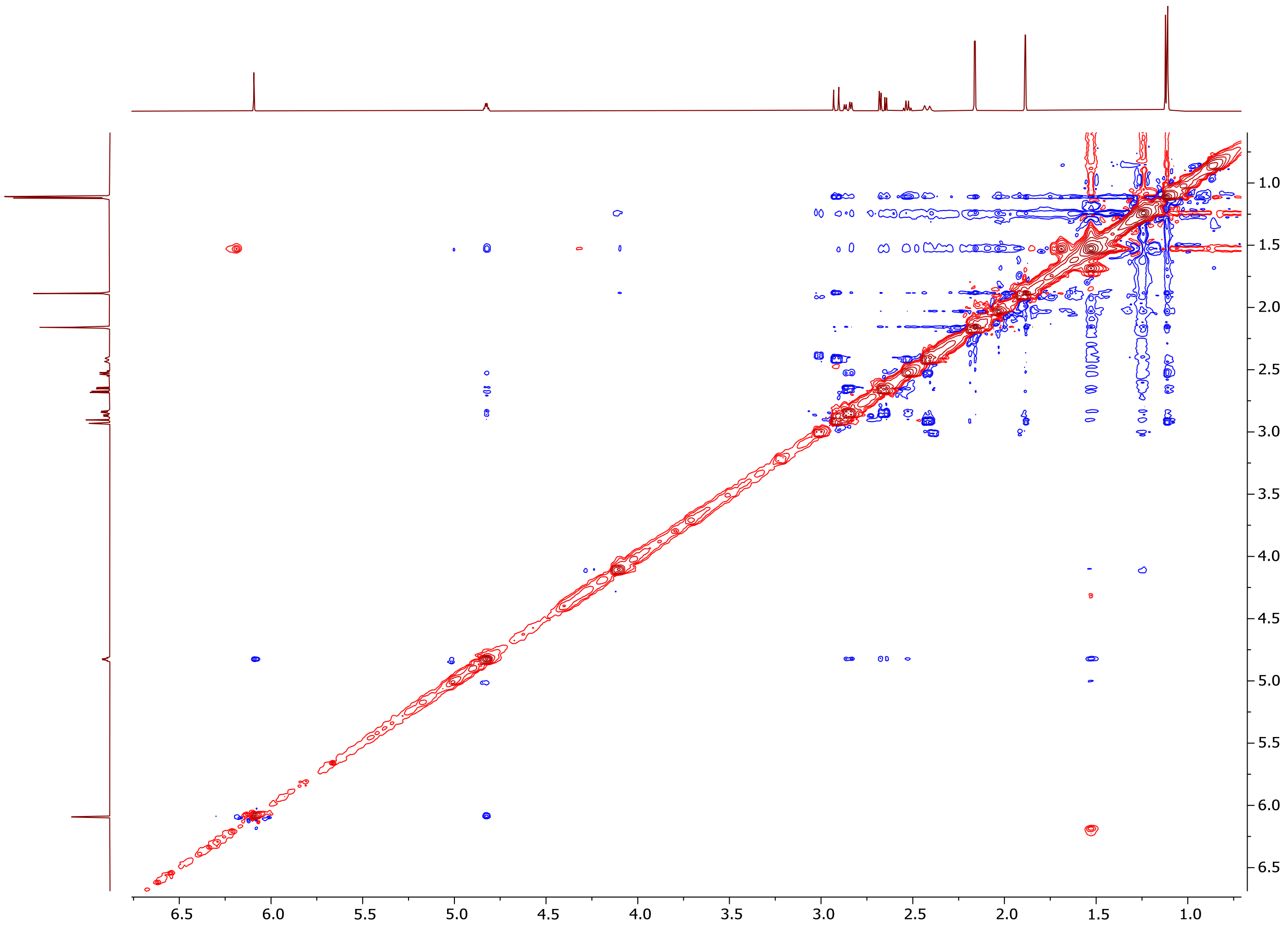

Figure S13. 2D NOESY spectrum of compound **2**.

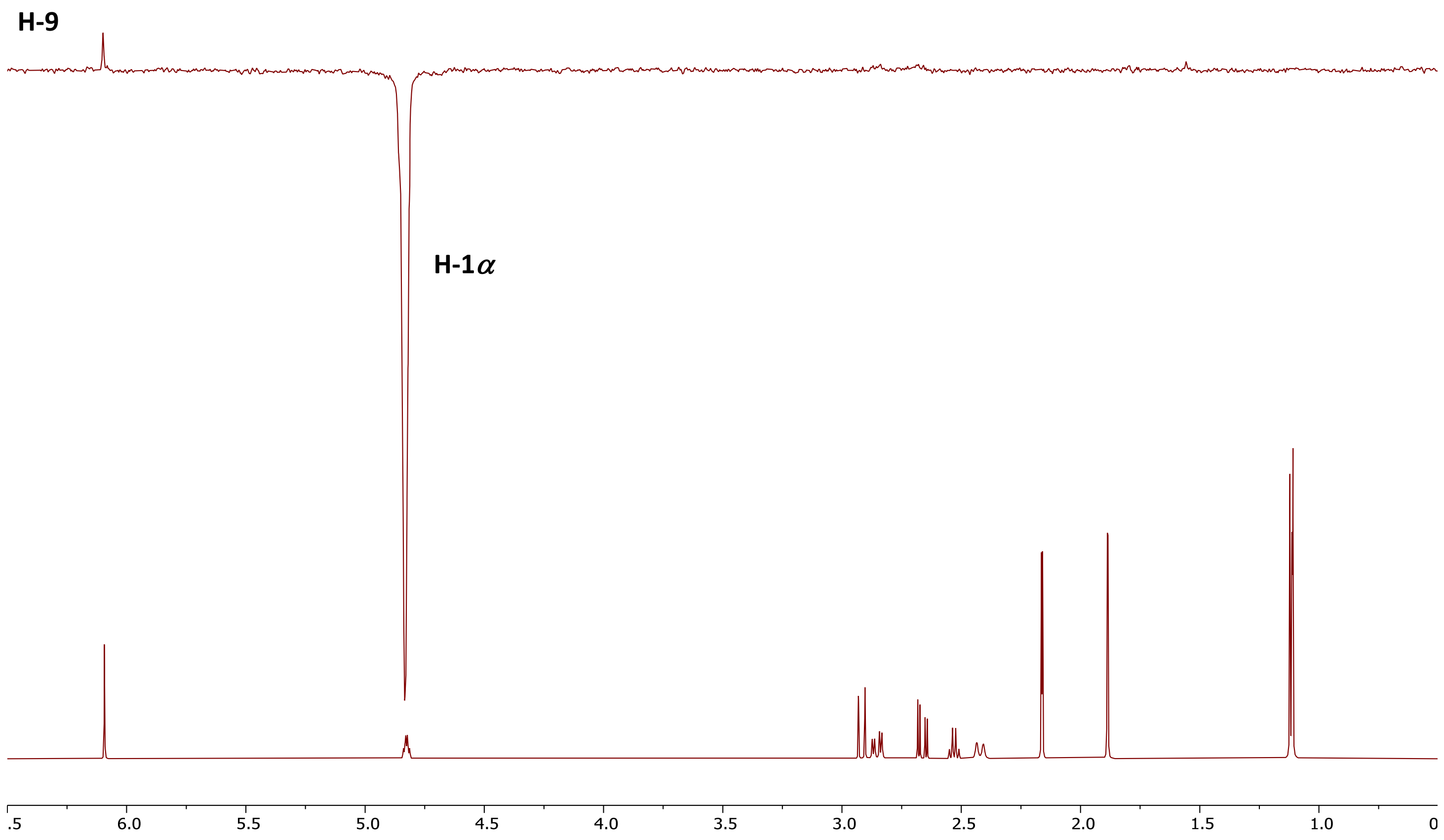

Figure S14a. 1D NOESY spectrum of compound **2**.

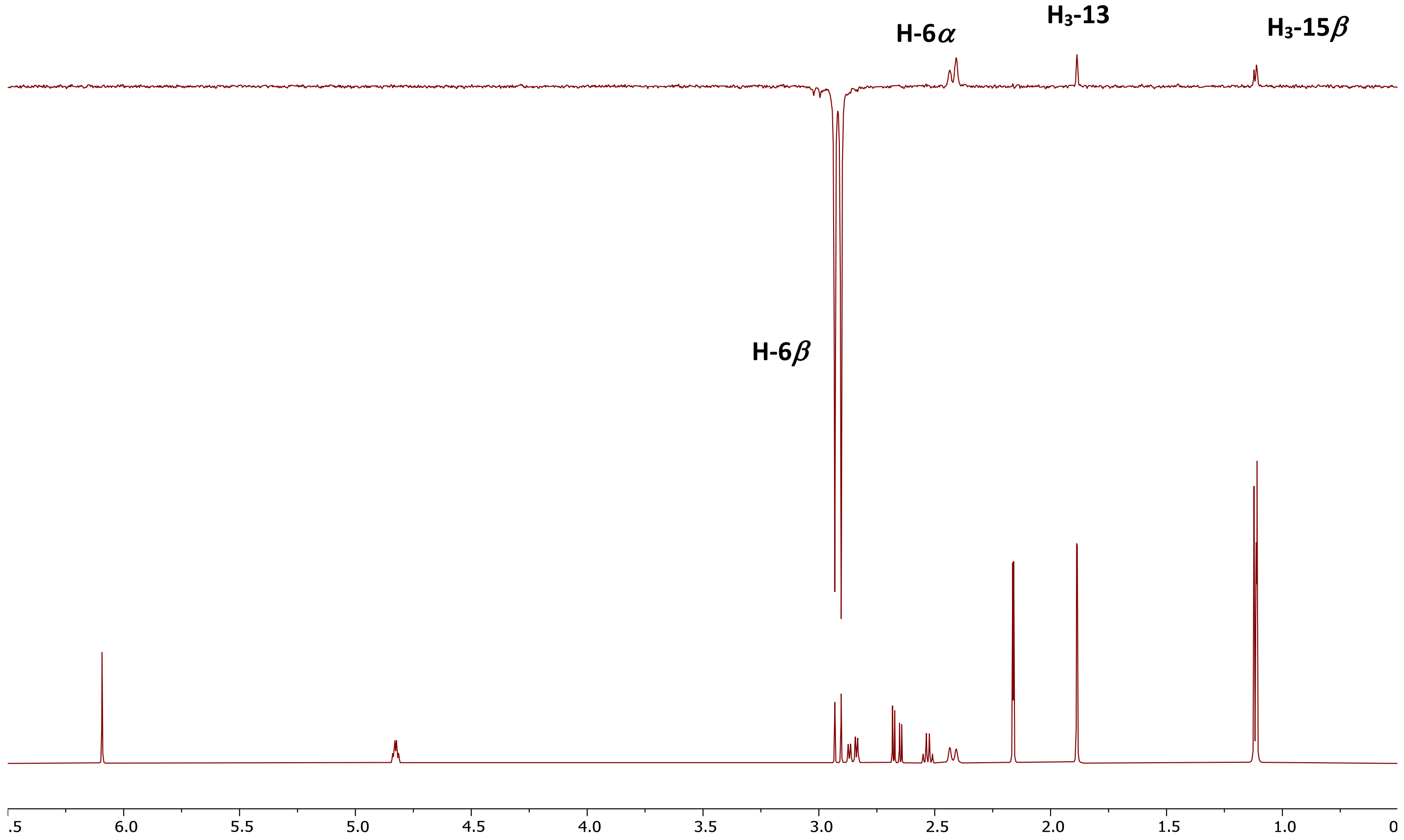

Figure S14b. 1D NOESY spectrum of compound **2**.

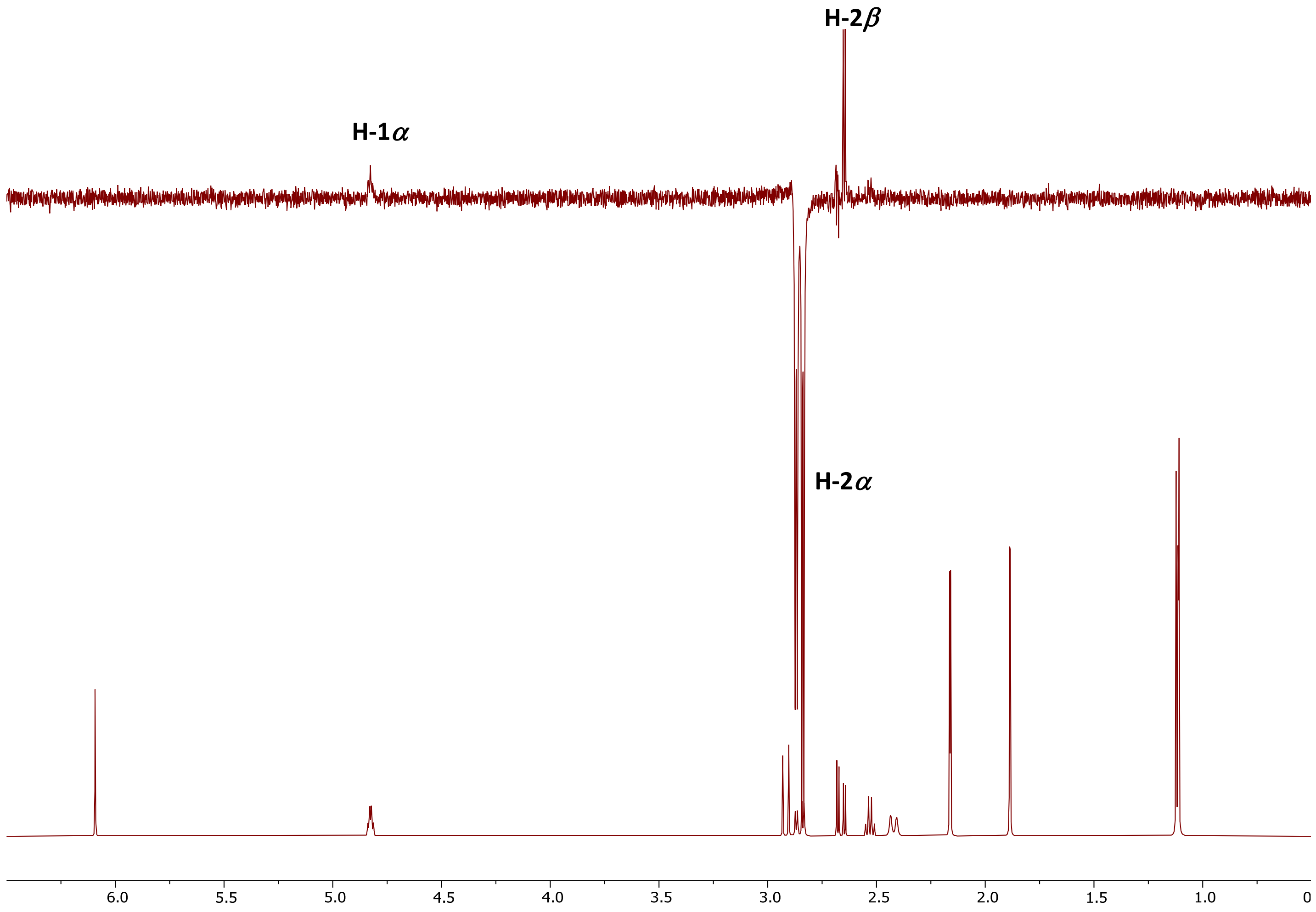

Figure S14c. 1D NOESY spectrum of compound **2**.

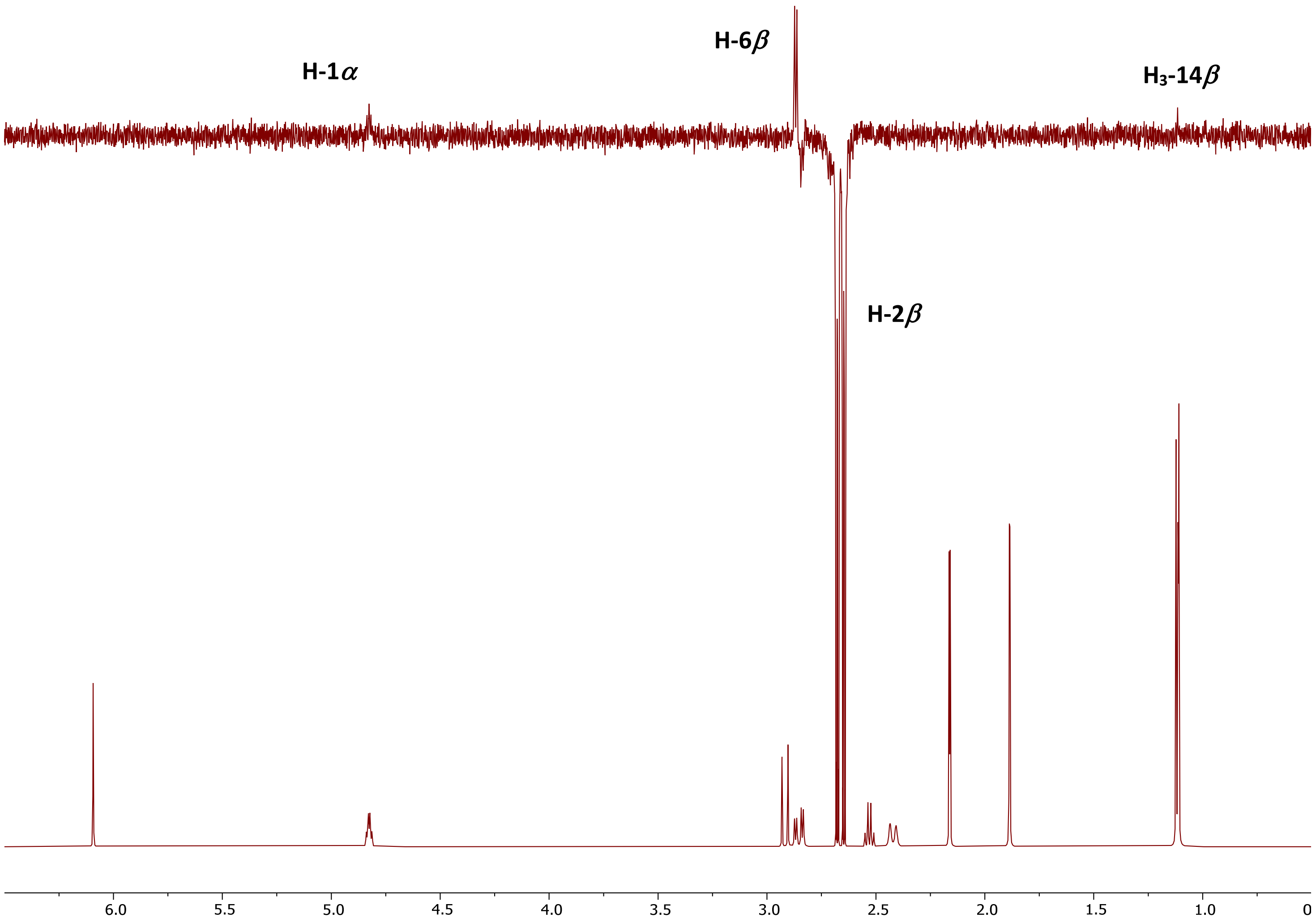

Figure S14d. 1D NOESY spectrum of compound **2**.

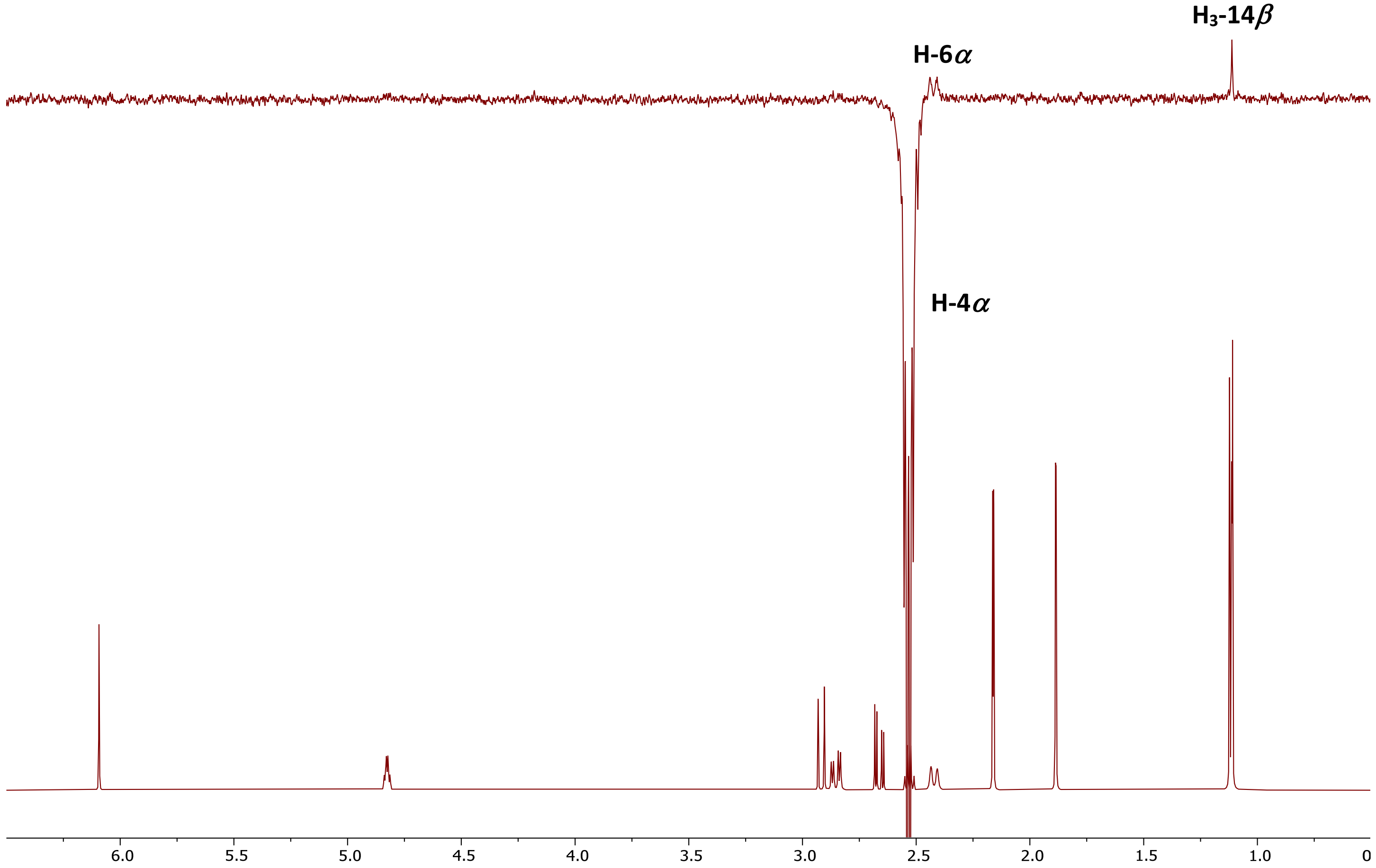

Figure S14e. 1D NOESY spectrum of compound **2**.

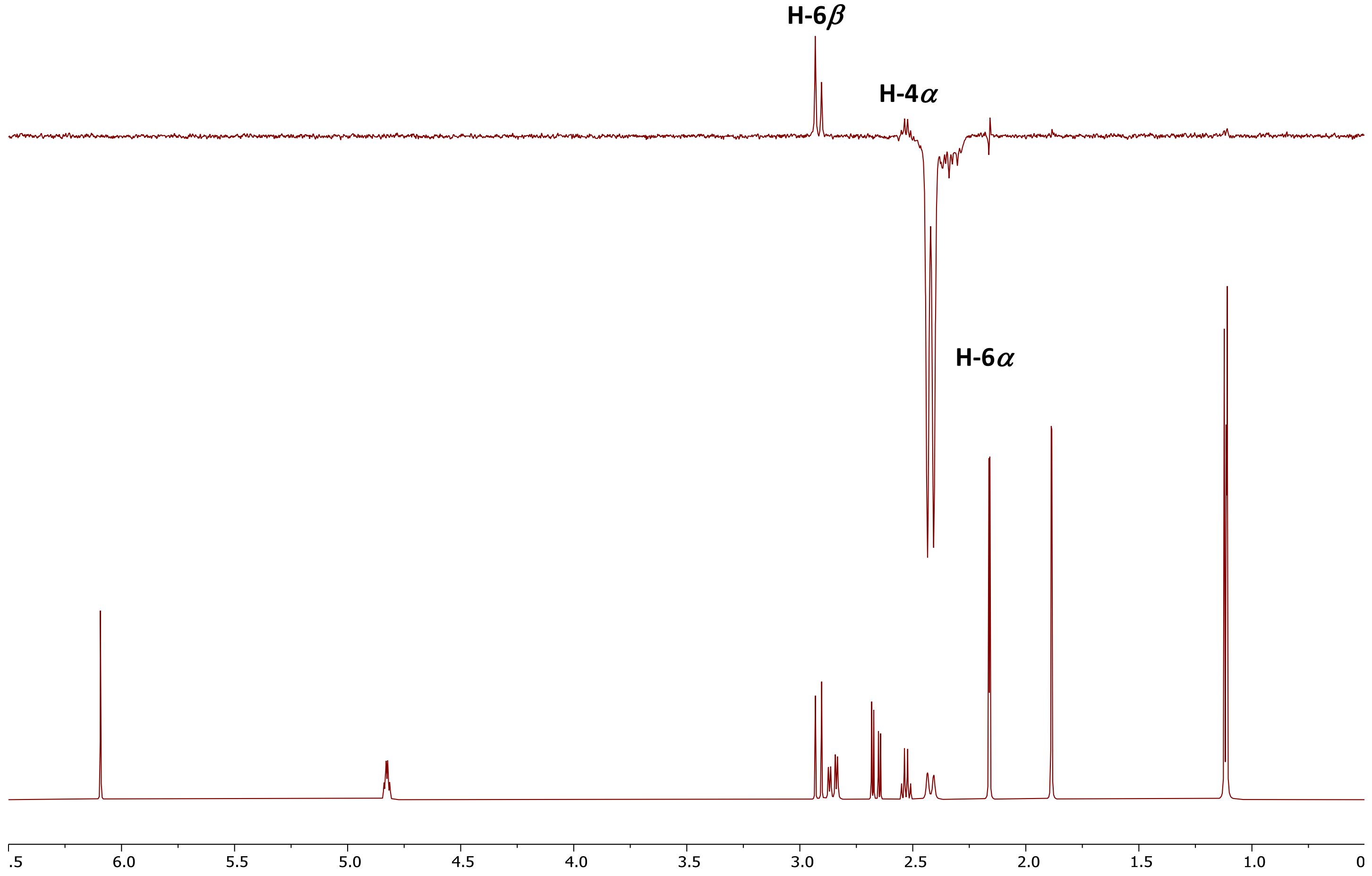

Figure S14f. 1D NOESY spectrum of compound **2**.

Single Mass Analysis

Tolerance = 5.0 mDa / DBE: min = -1.5, max = 80.0
Element prediction: Off
Number of isotope peaks used for i-FIT = 3

Monoisotopic Mass, Even Electron Ions
197 formula(e) evaluated with 1 results within limits (up to 5 closest results for each mass)
Elements Used:
C: 0-100 H: 0-100 O: 0-50 Cl: 0-20 Br: 0-8

2-15-F50-NEG 72 (1.335)
1: TOF MS ES-

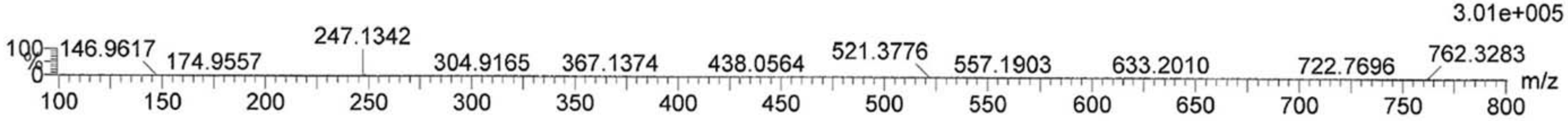

Minimum:				-1.5				
Maximum:		5.0	5.0	80.0				
Mass	Calc. Mass	mDa	PPM	DBE	i-FIT	Norm	Conf(%)	Formula
247.1342	247.1334	0.8	3.2	6.5	289.6	n/a	n/a	C15 H19 O3

Figure S15. HRESIMS spectrum of compound **2**.

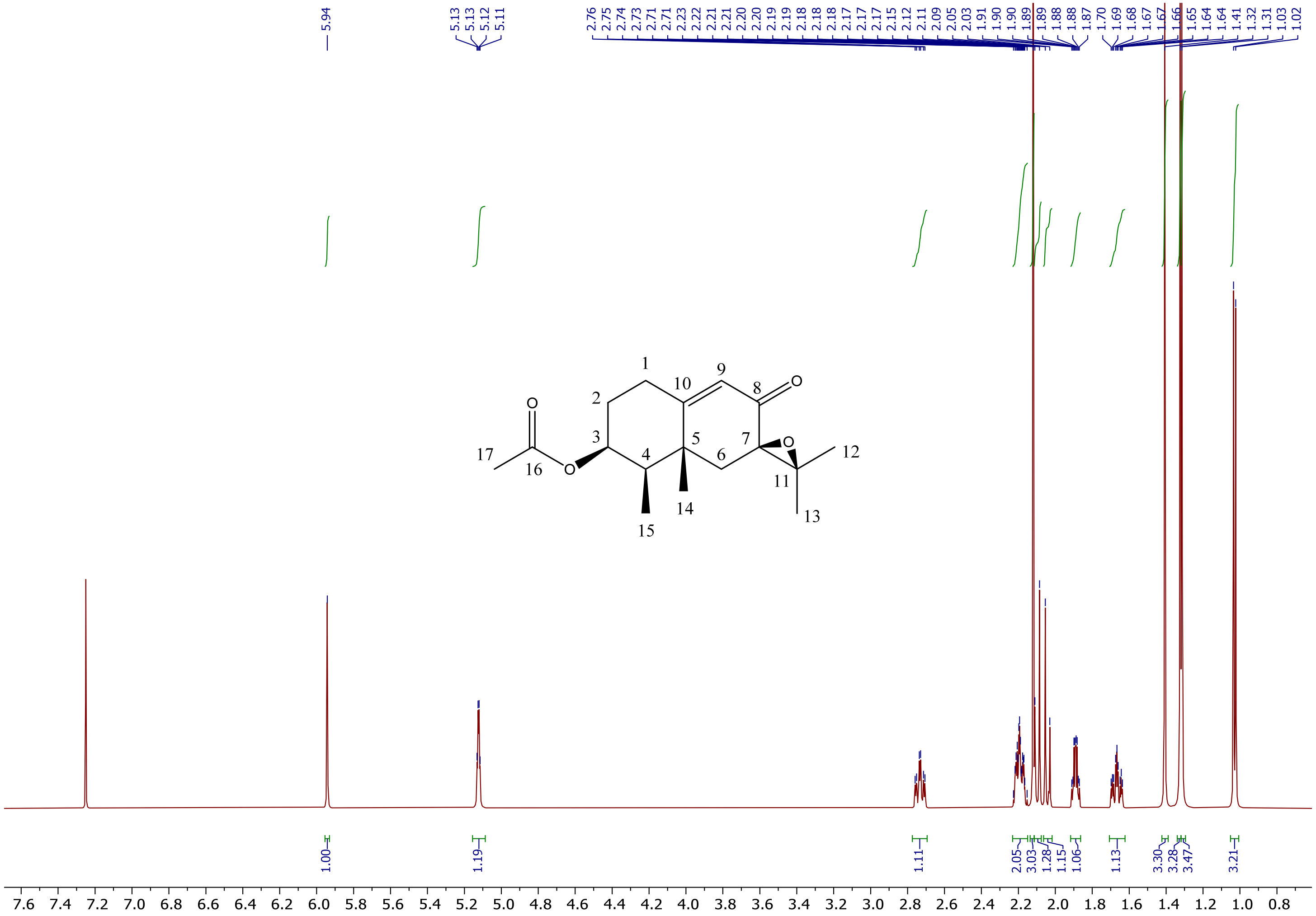

Figure S16. ^{1}H NMR (400 MHz, $CDCl_3$) spectrum of compound **3**.

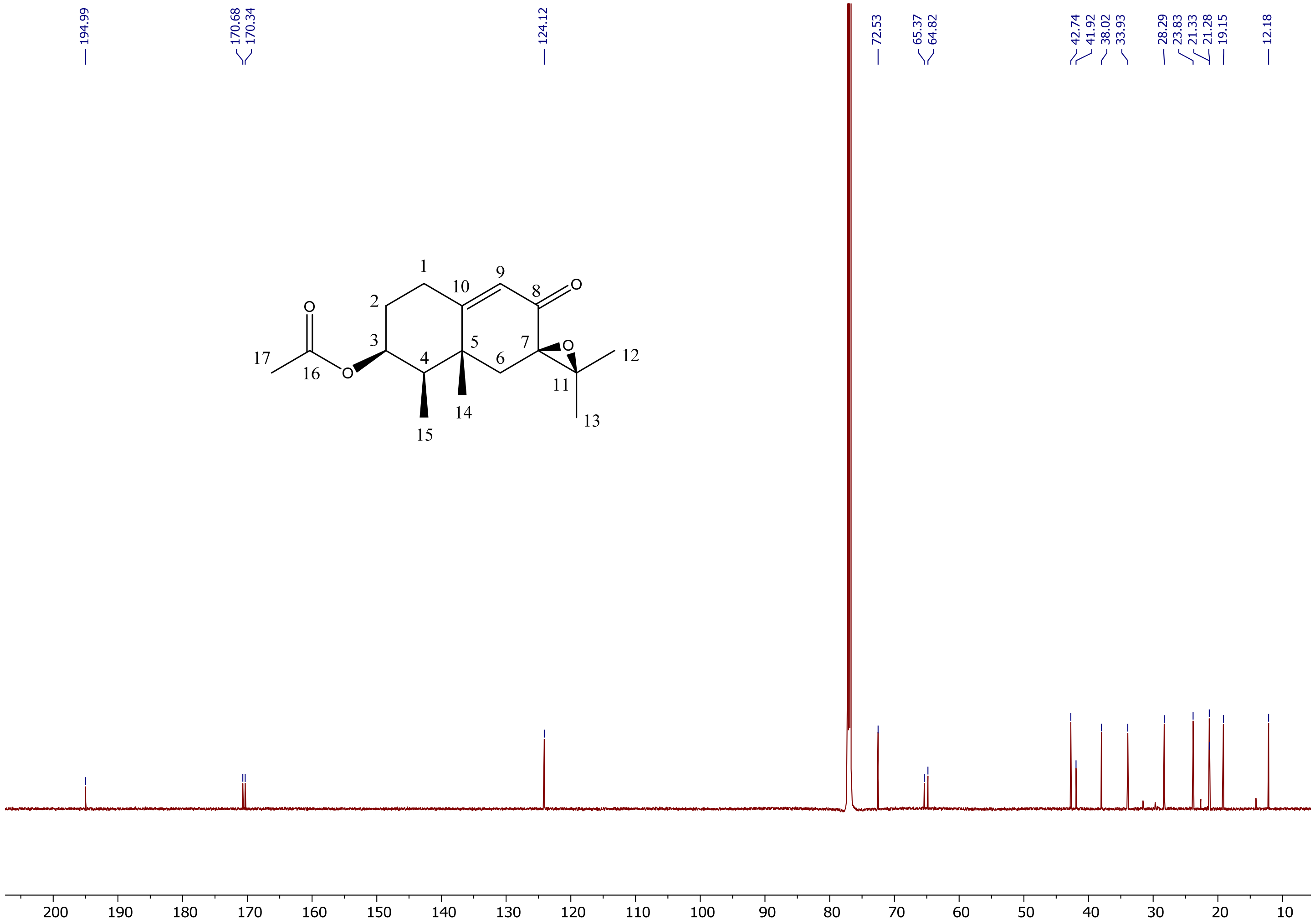

Figure S17. ^{13}C NMR (100 MHz, $CDCl_3$) spectrum of compound **3**.

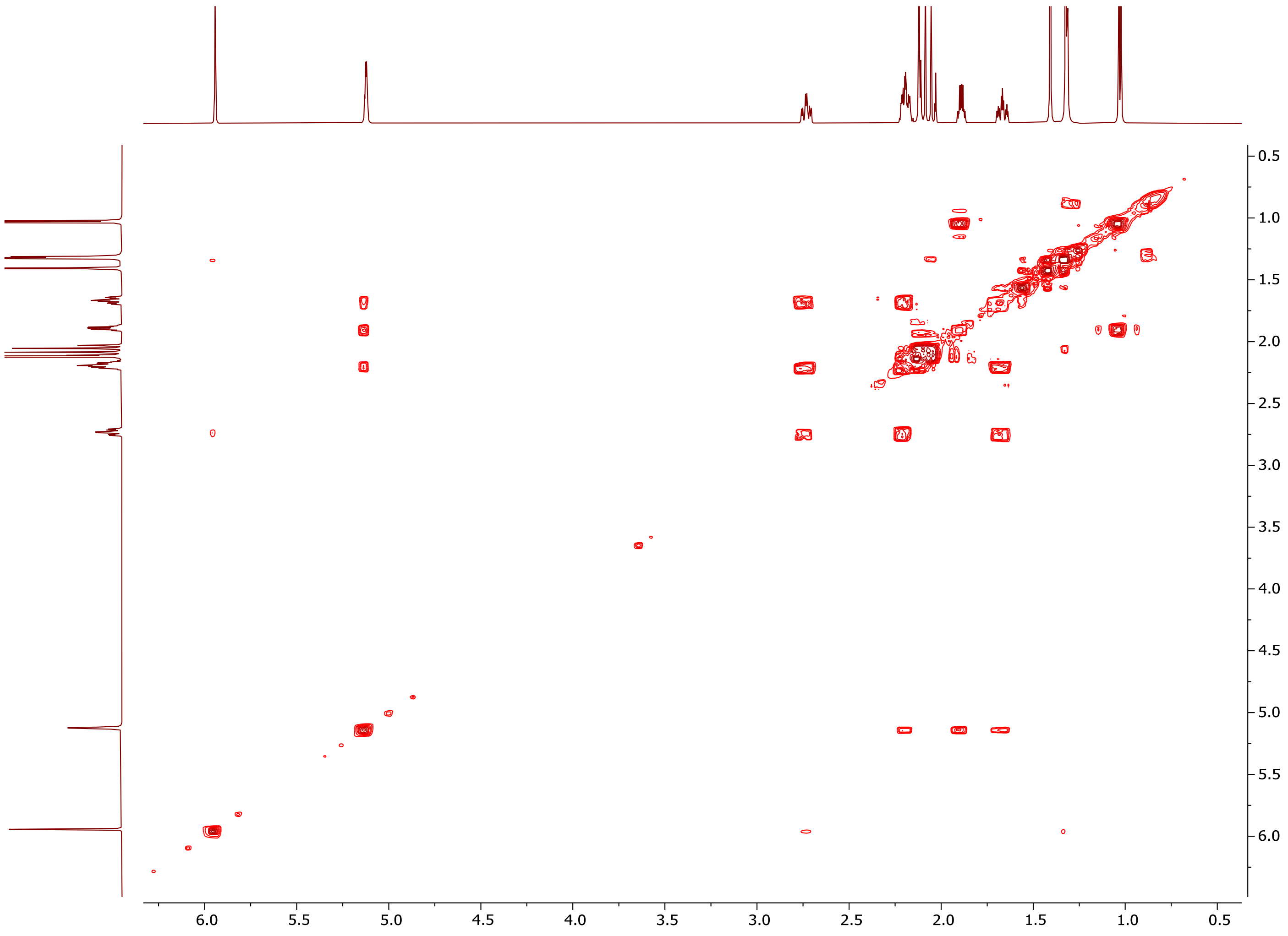

Figure S18. gCOSY spectrum of compound **3**.

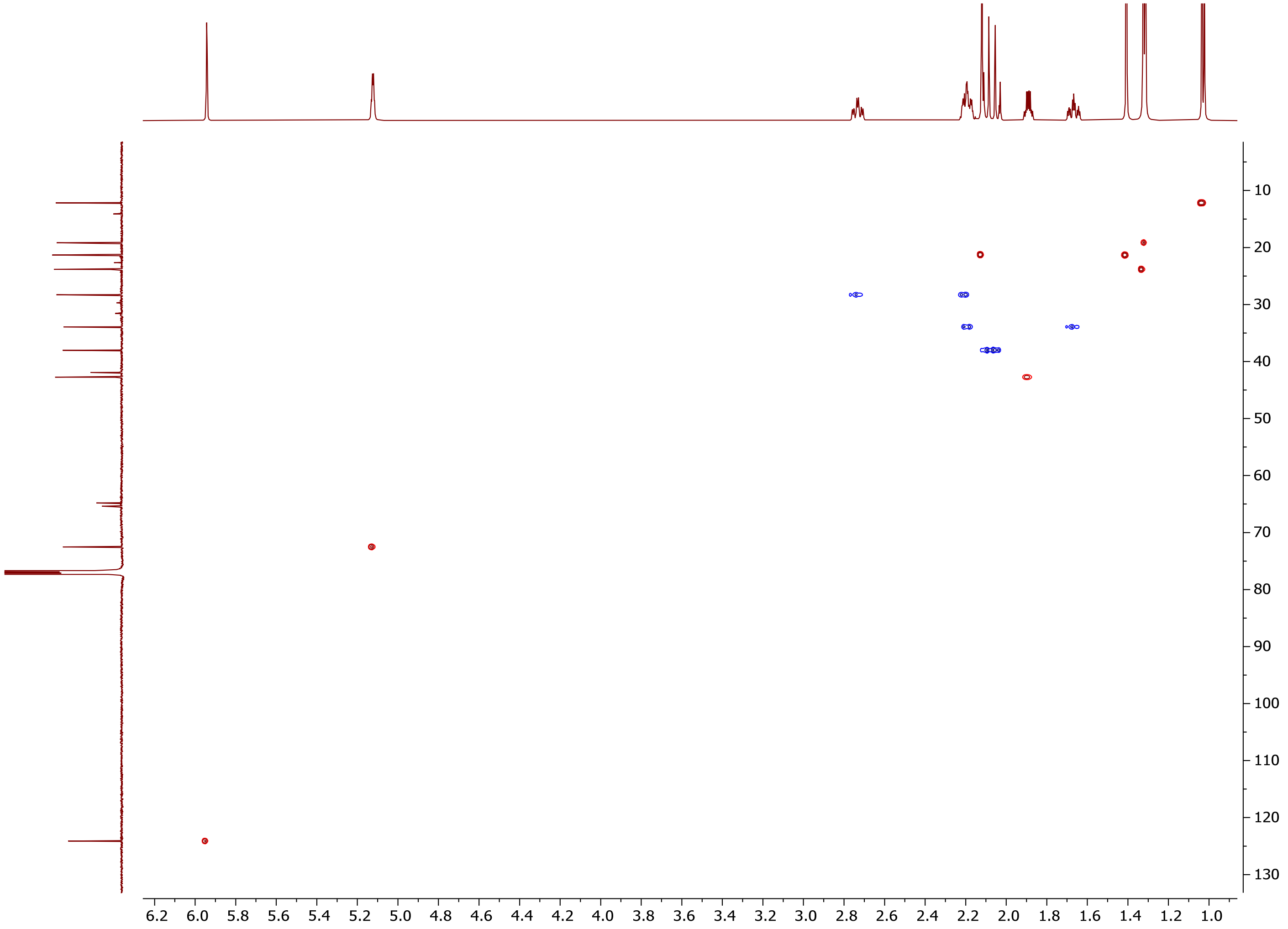

Figure S19. gHSQC spectrum of compound **3**.

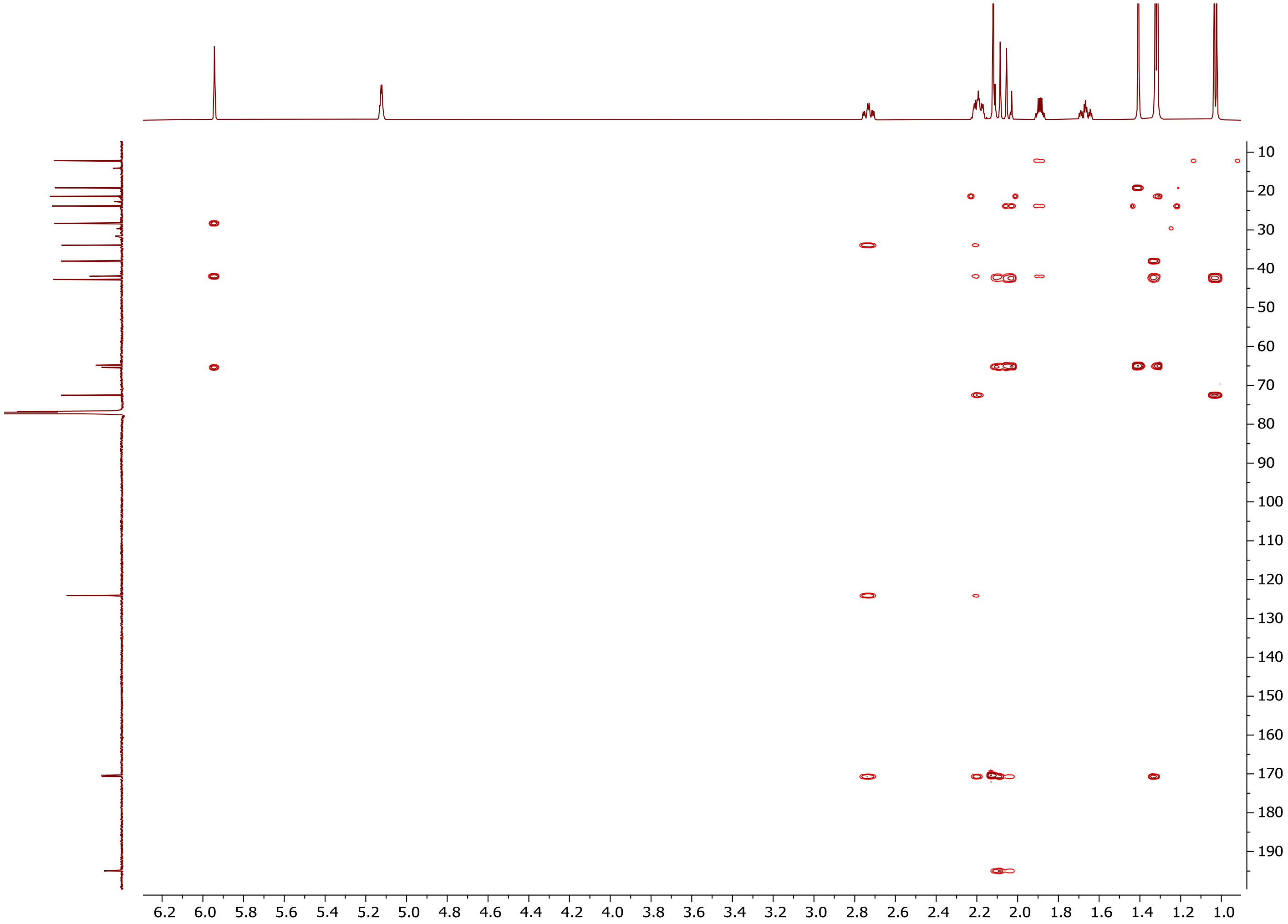

Figure S20. gHMBC spectrum of compound **3**.

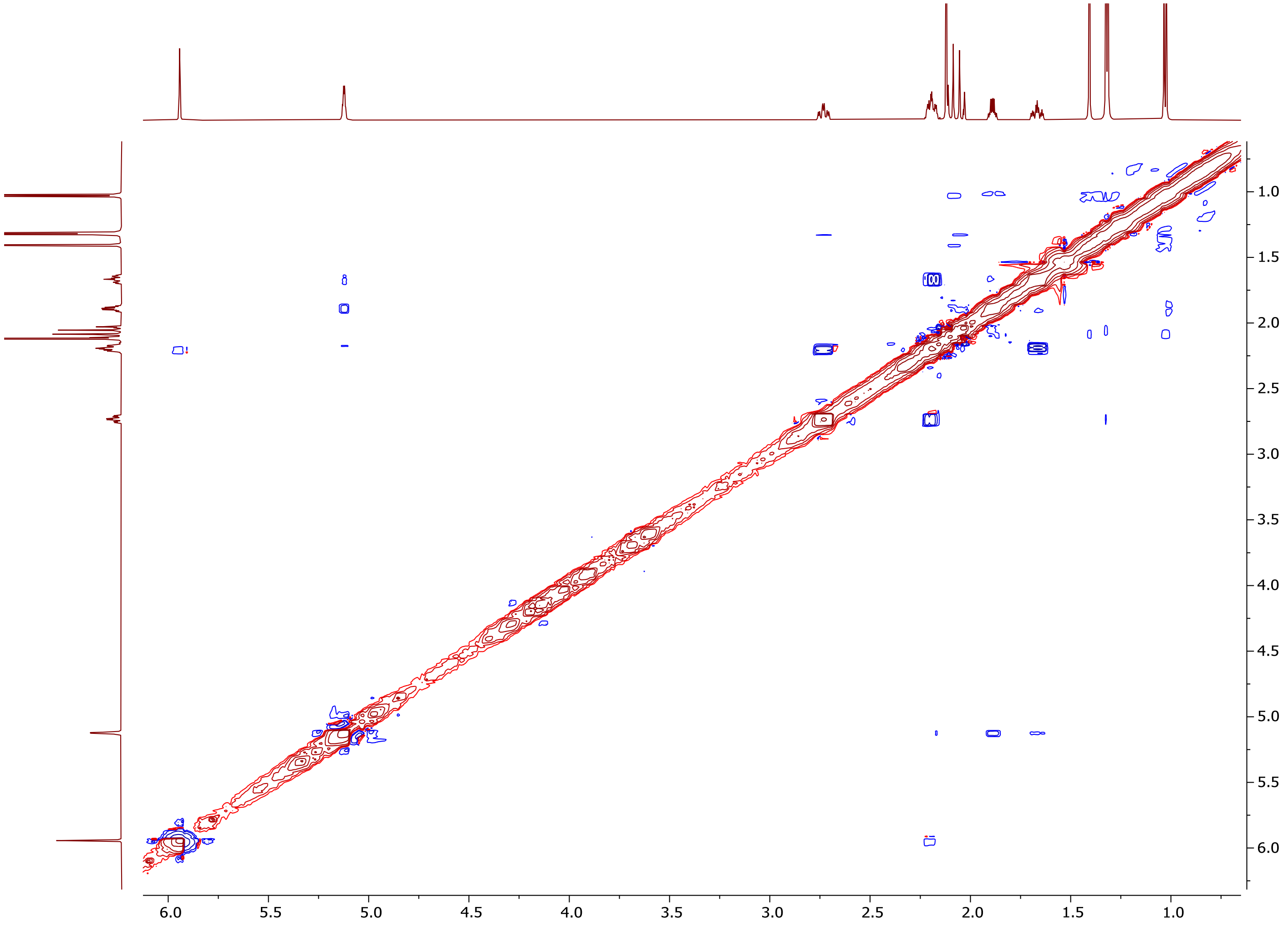

Figure S21. 2D NOESY spectrum of compound **3**.

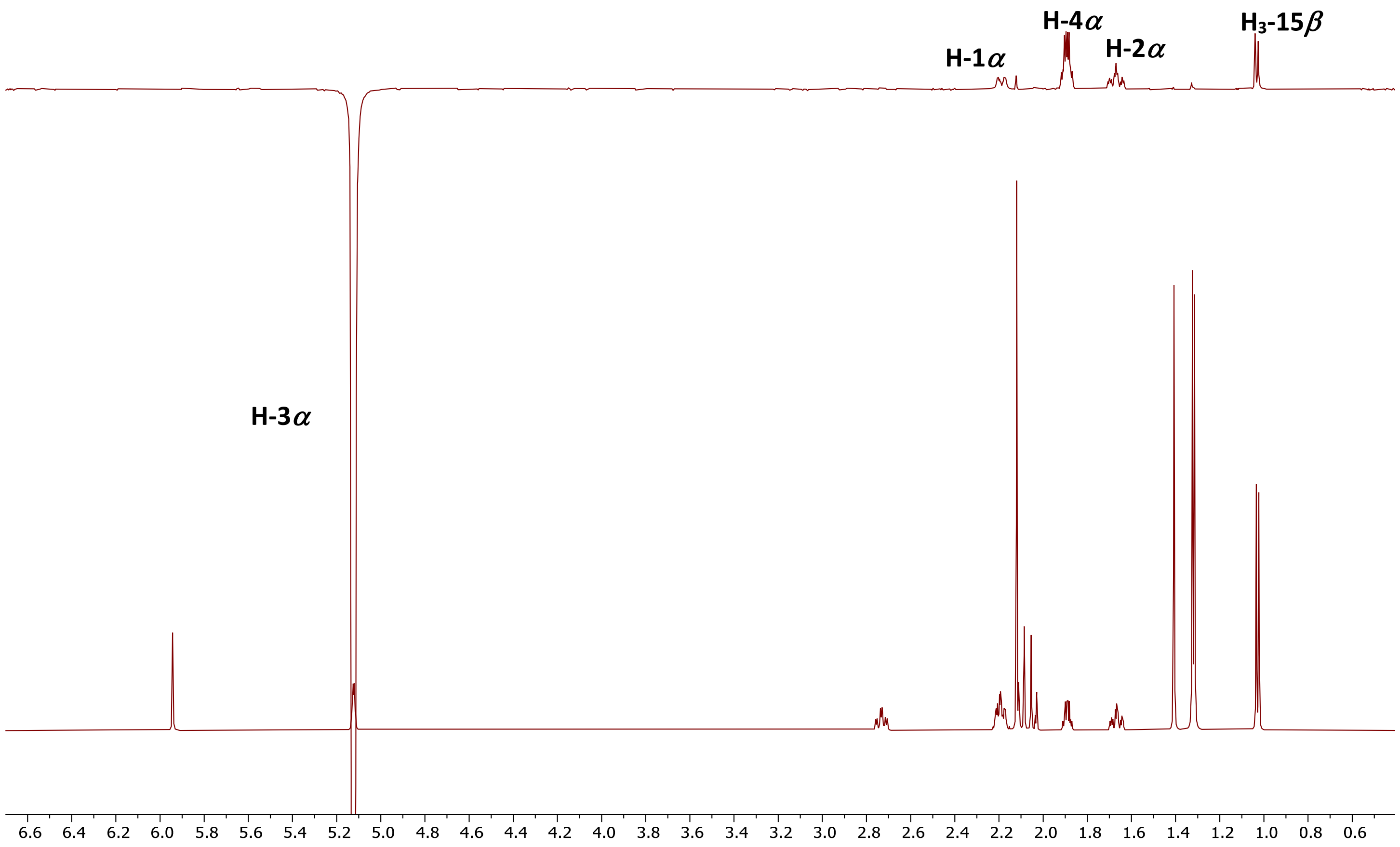

Figure S22a. 1D NOESY spectrum of compound **3**.

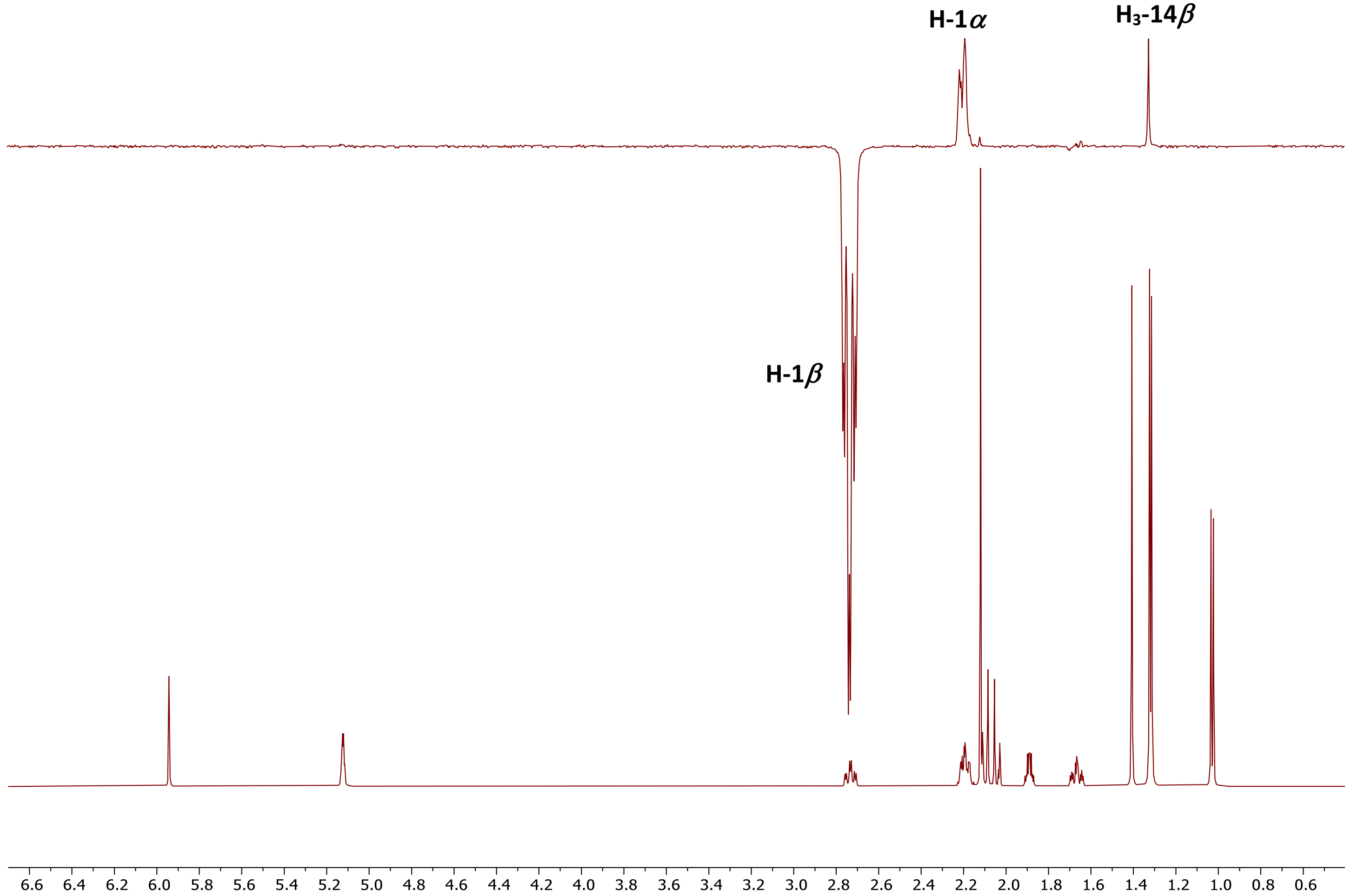

Figure S22b. 1D NOESY spectrum of compound **3**.

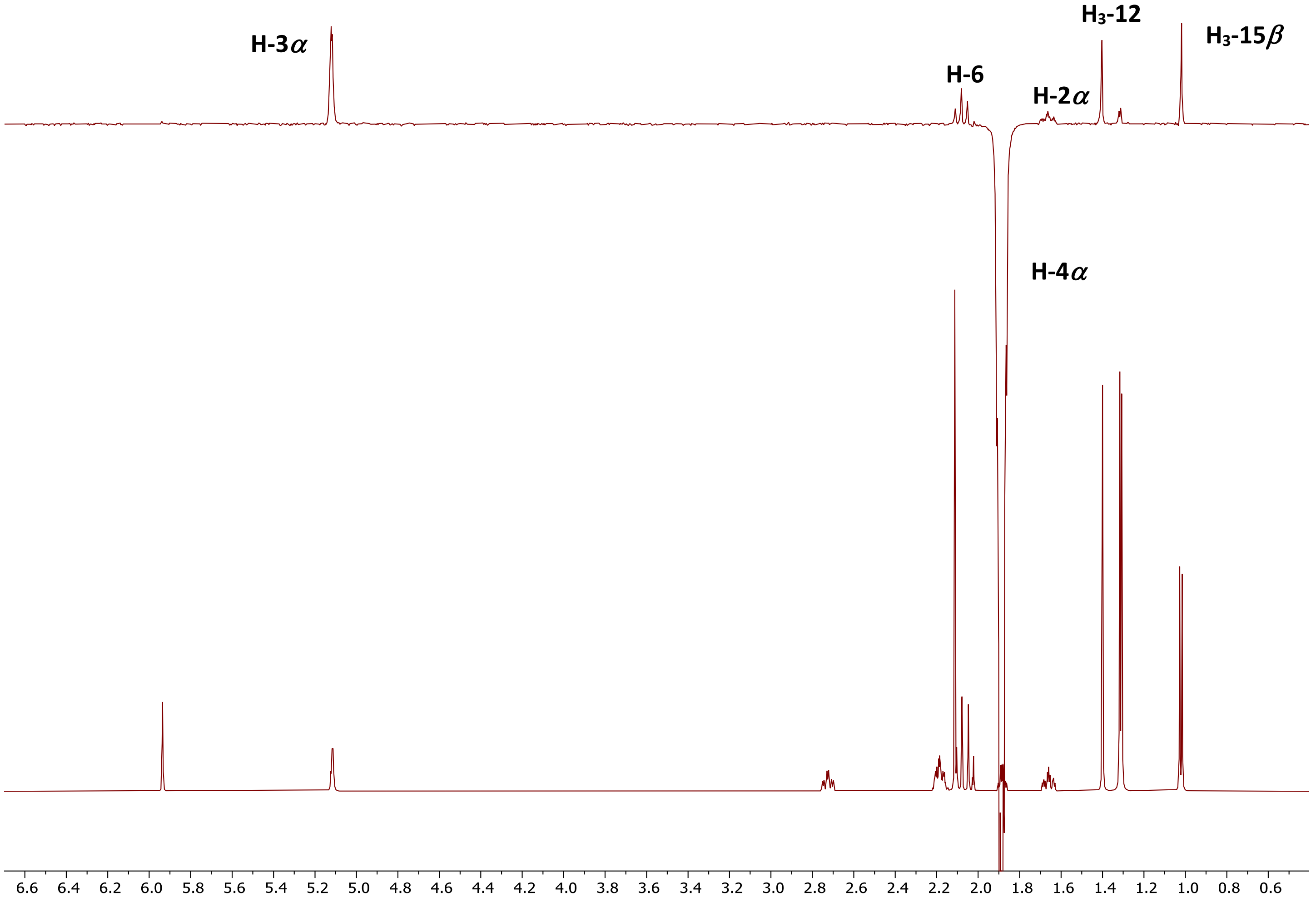

Figure S22c. 1D NOESY spectrum of compound **3**.

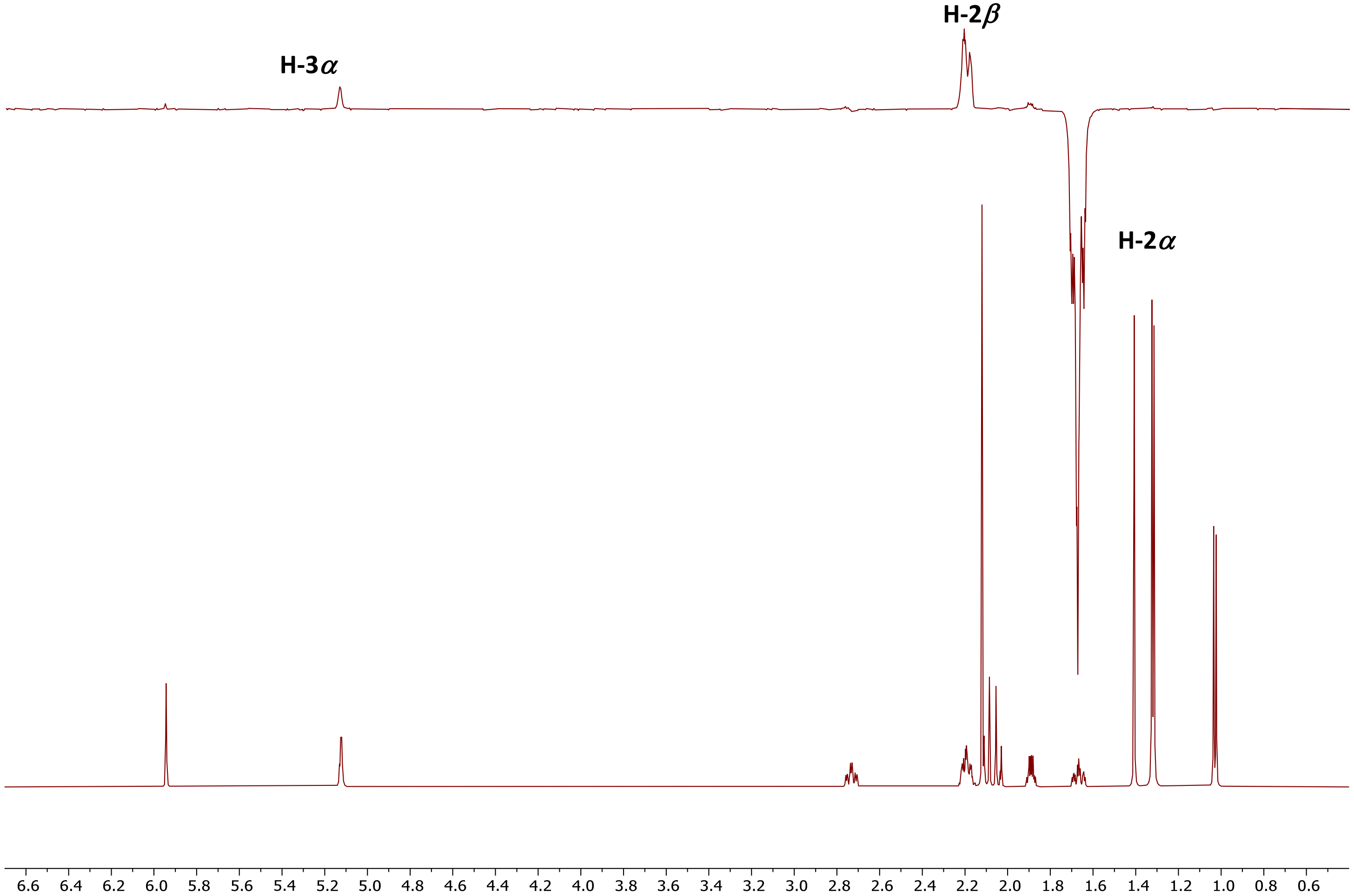

Figure S22d. 1D NOESY spectrum of compound **3**.

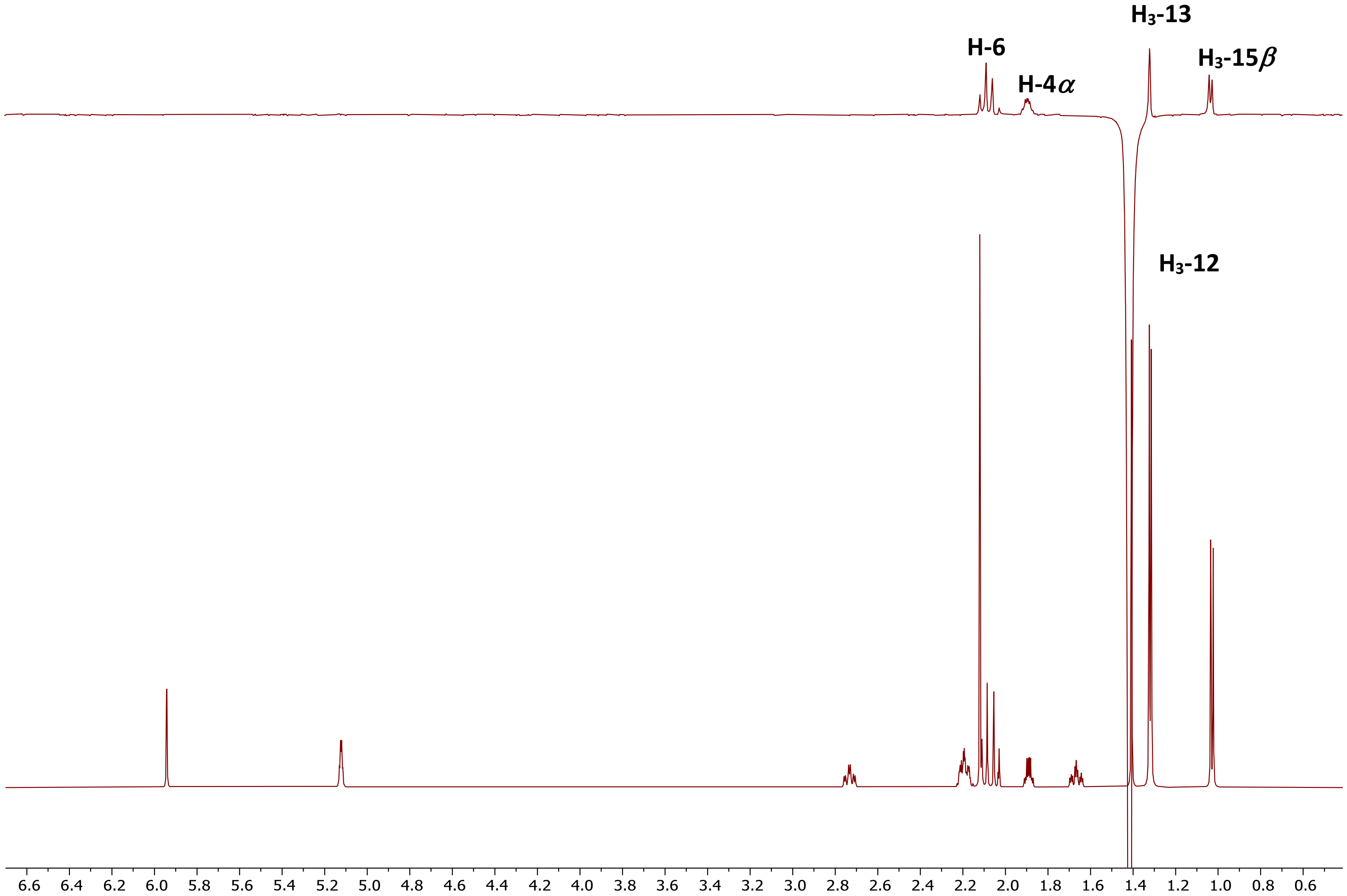

Figure S22e. 1D NOESY spectrum of compound **3**.

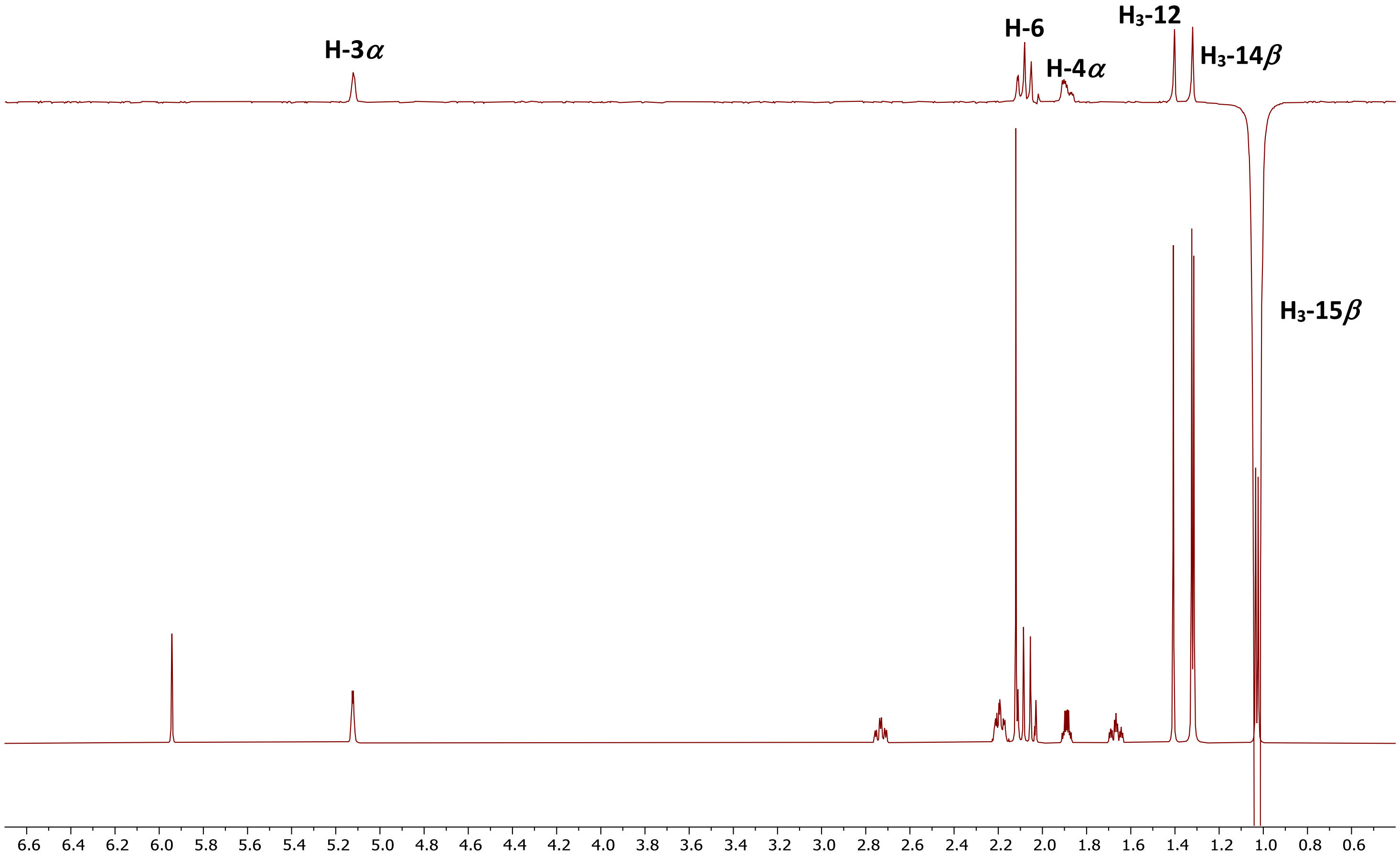

Figure S22f. 1D NOESY spectrum of compound **3**.

Single Mass Analysis

Tolerance = 5.0 mDa / DBE: min = -1.5, max = 80.0
Element prediction: Off
Number of isotope peaks used for i-FIT = 5

Monoisotopic Mass, Even Electron Ions
221 formula(e) evaluated with 1 results within limits (up to 5 closest results for each mass)
Elements Used:
C: 0-100 H: 0-100 O: 0-50 Na: 0-1 Br: 0-8

EMIF40BC30P2 103 (1.912)
1: TOF MS ES+

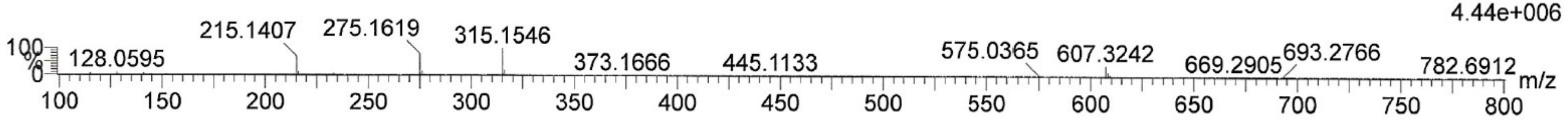

Minimum:				-1.5				
Maximum:		5.0	5.0	80.0				
Mass	Calc. Mass	mDa	PPM	DBE	i-FIT	Norm	Conf(%)	Formula
315.1546	315.1572	-2.6	-8.2	5.5	1290.1	n/a	n/a	C17 H24 O4 Na

Figure S23. HRESIMS spectrum of compound **3**.

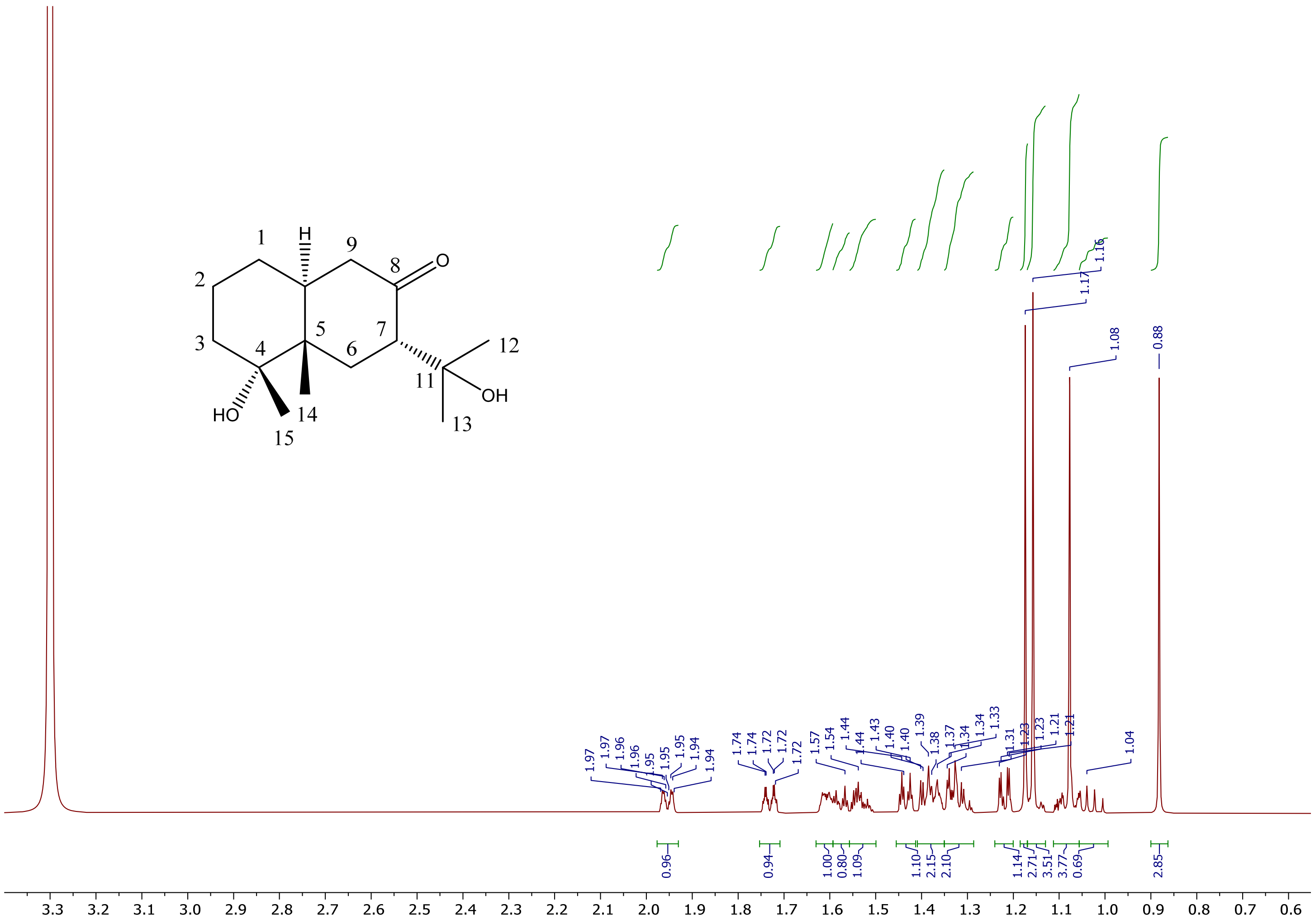

Figure S24a. ^{1}H NMR spectrum (700 MHz, CD_3OD) of compound **4**.

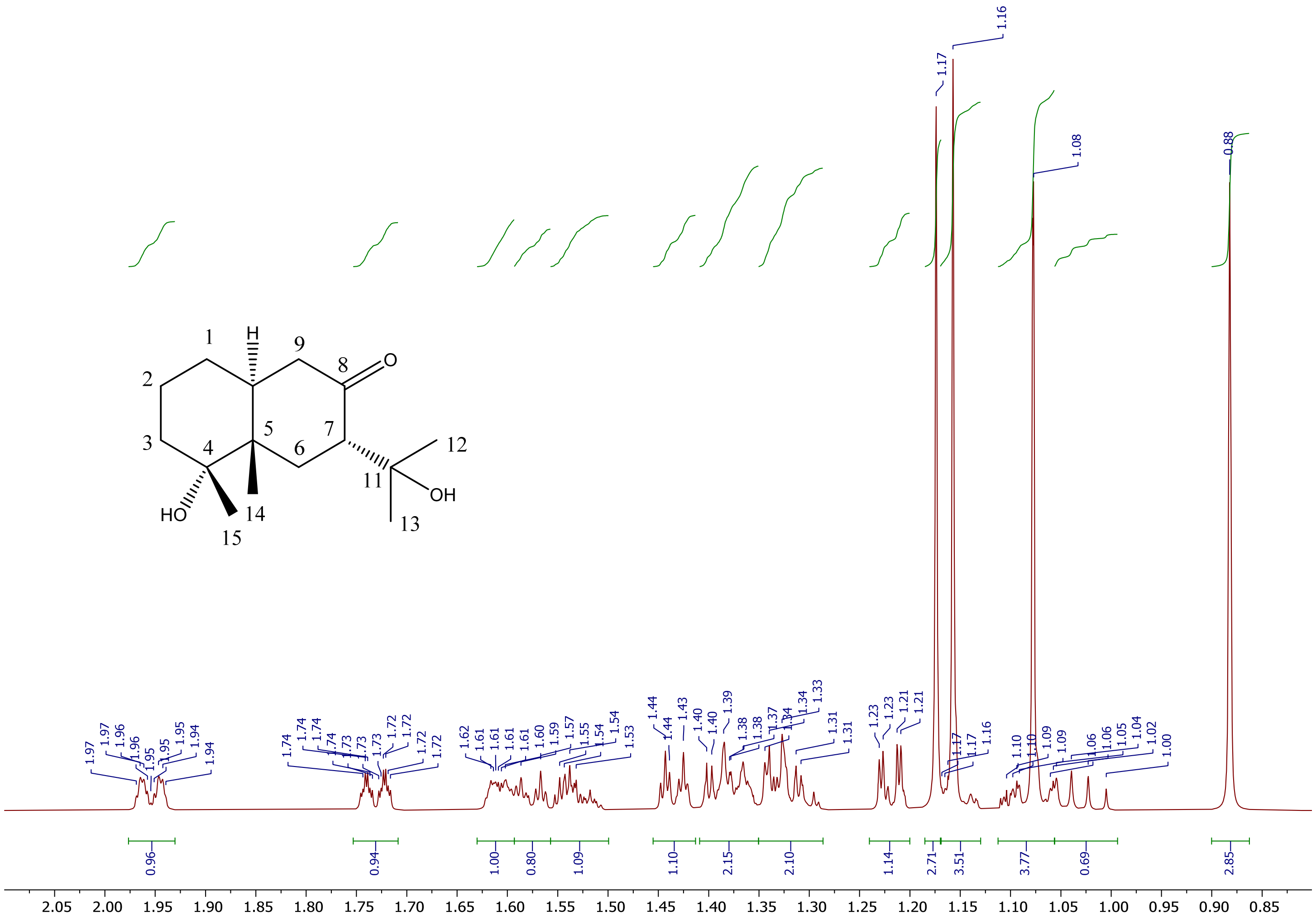

Figure S24b. Expansion of ^{1}H NMR spectrum (700 MHz, CD_3OD) of compound **4**.

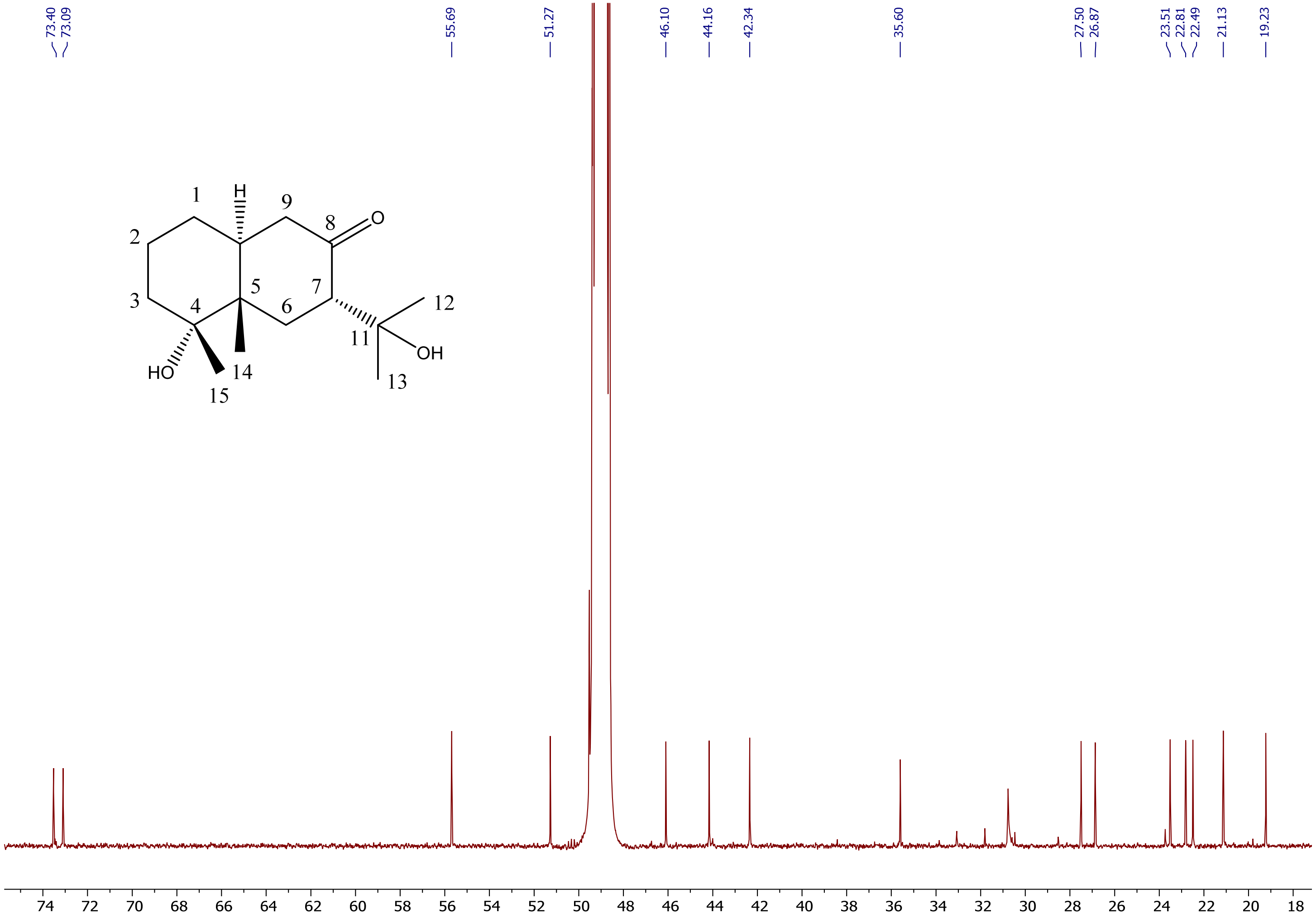

Figure S25. ^{13}C NMR spectrum (175 MHz, CD_3OD) of compound **4**.

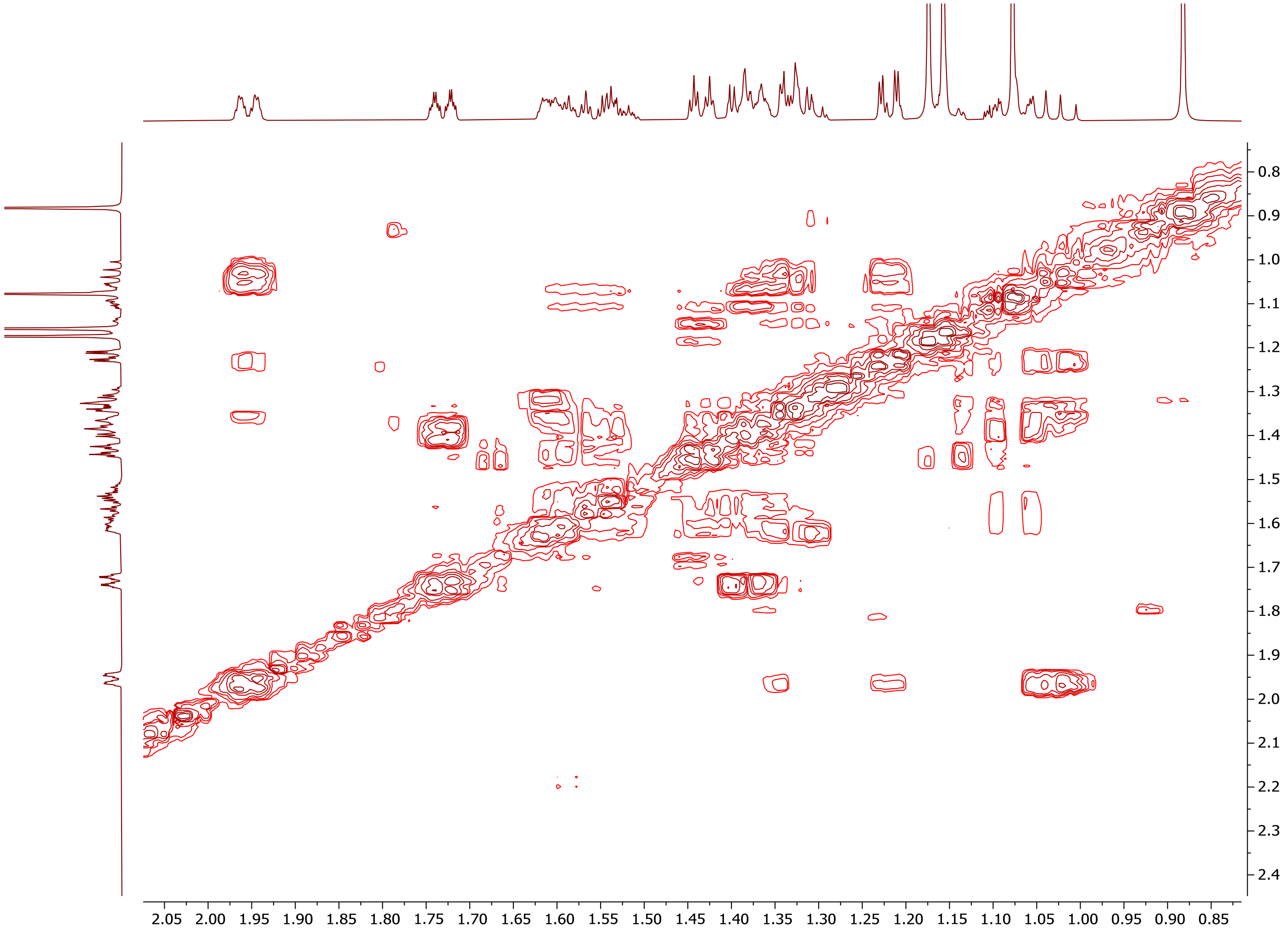

Figure S26. gCOSY spectrum of compound **4**.

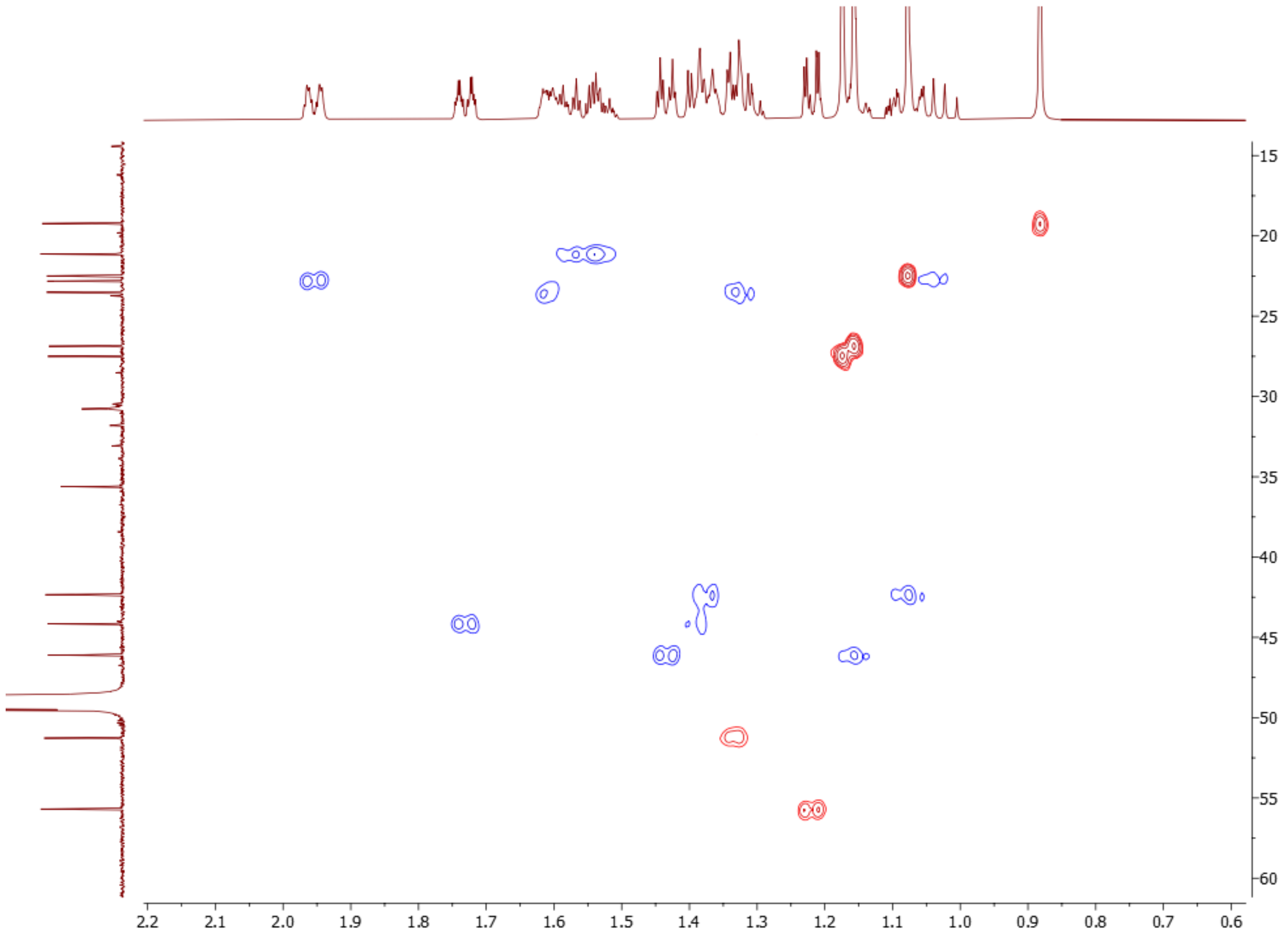

Figure S27. gHSQC spectrum of compound **4**.

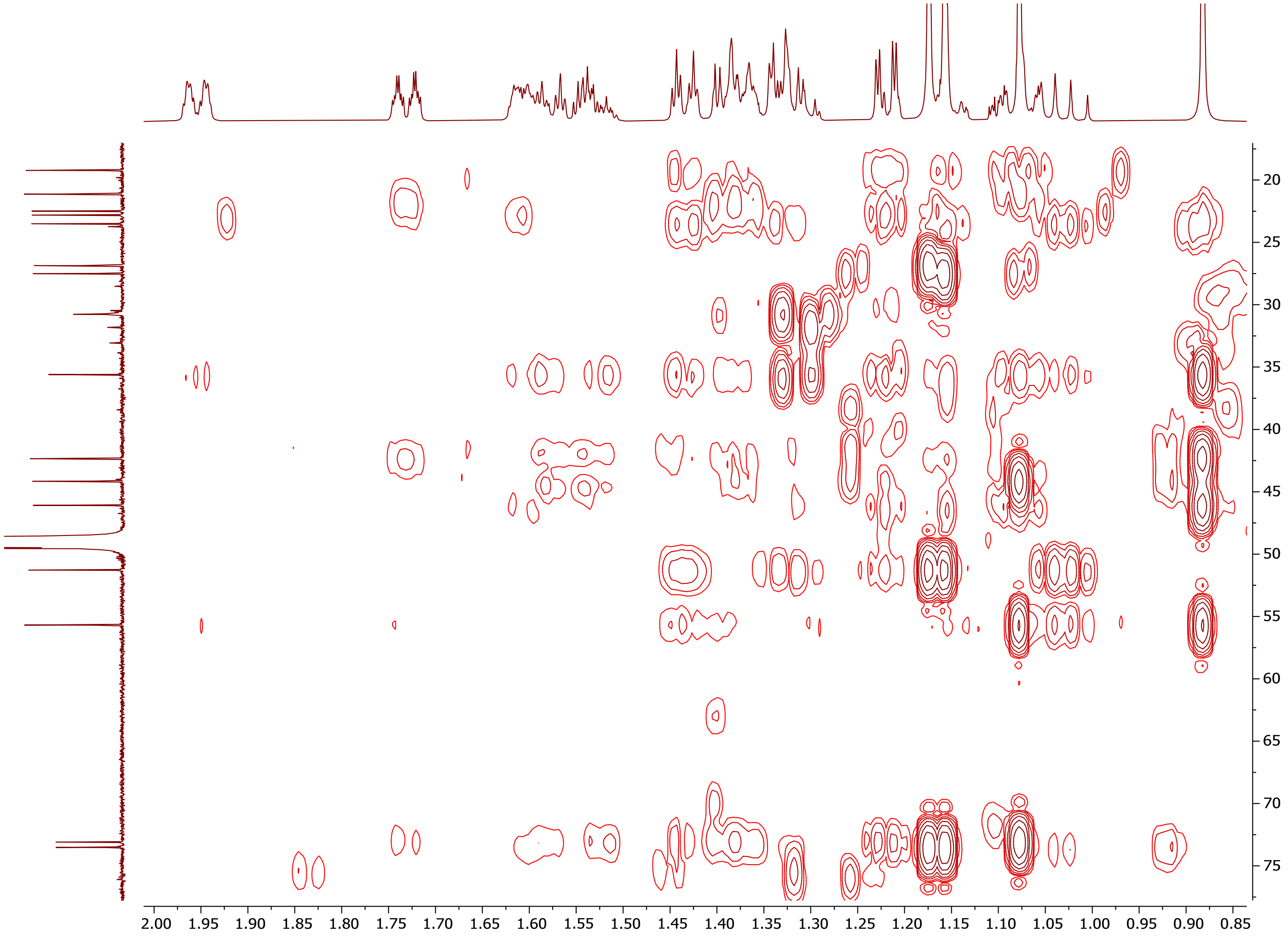

Figure S28. gHMBC spectrum of compound **4**.

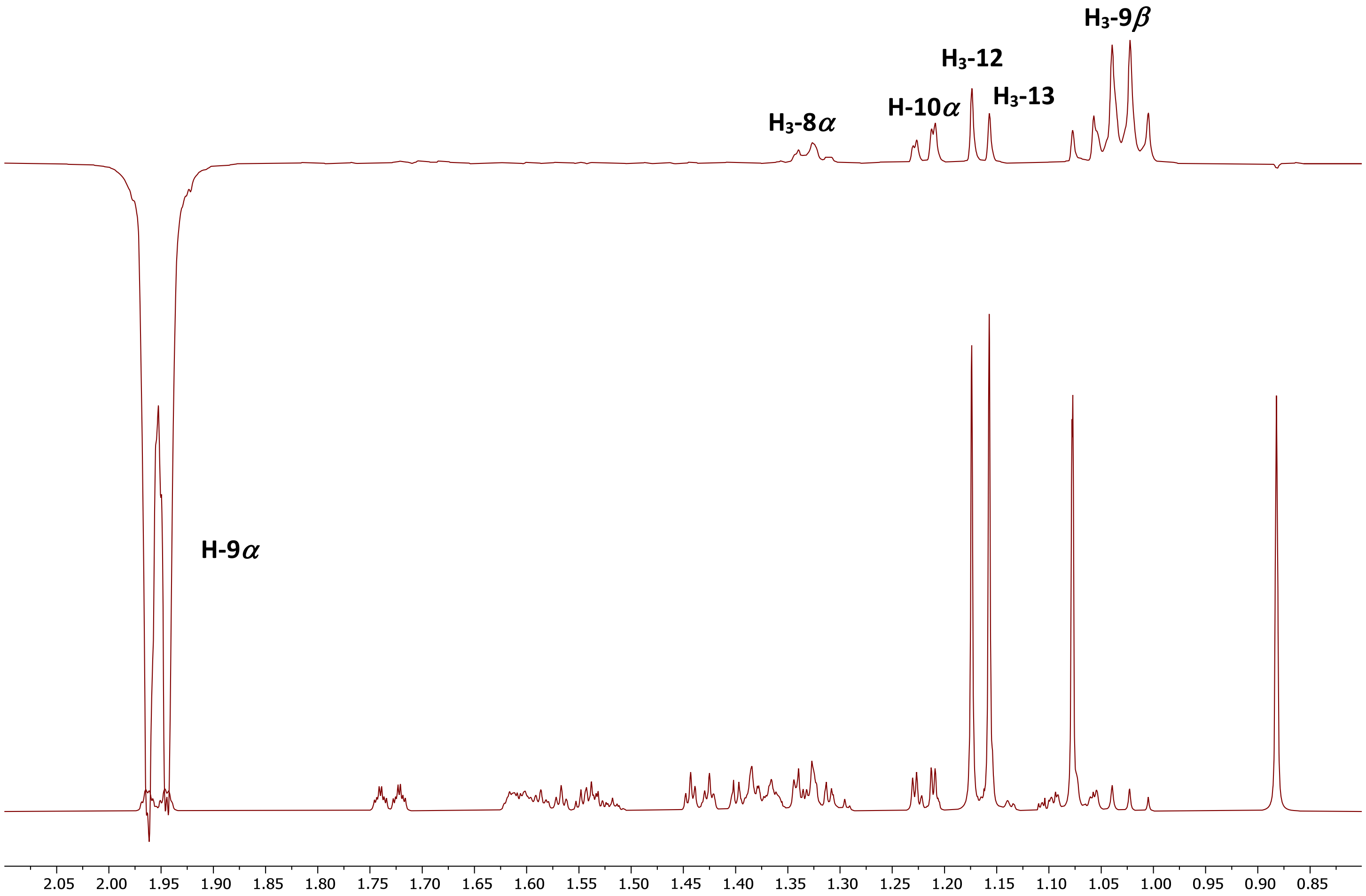

Figure S29a. 1D NOESY spectrum of compound **4**.

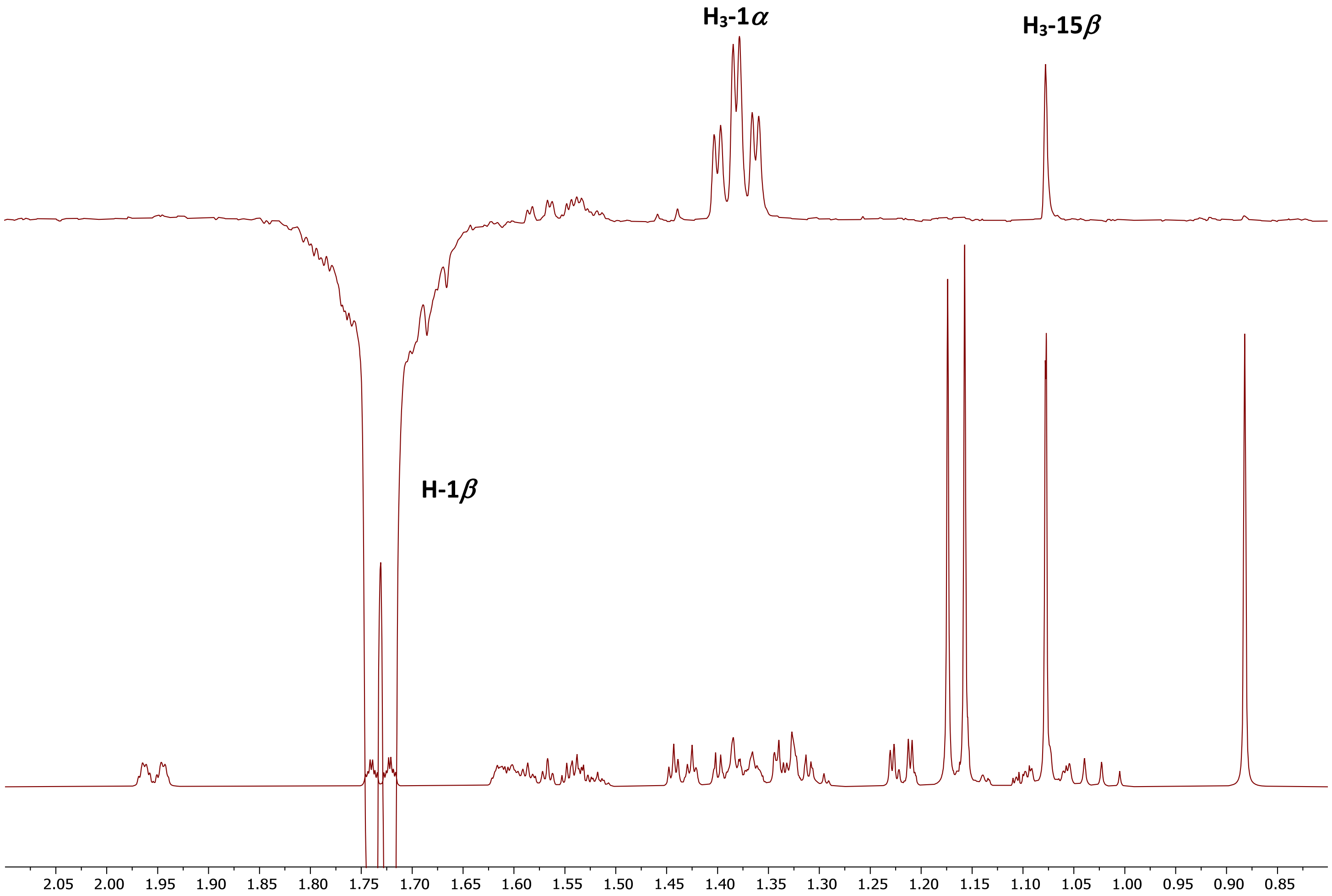

Figure S29b. 1D NOESY spectrum of compound **4**.

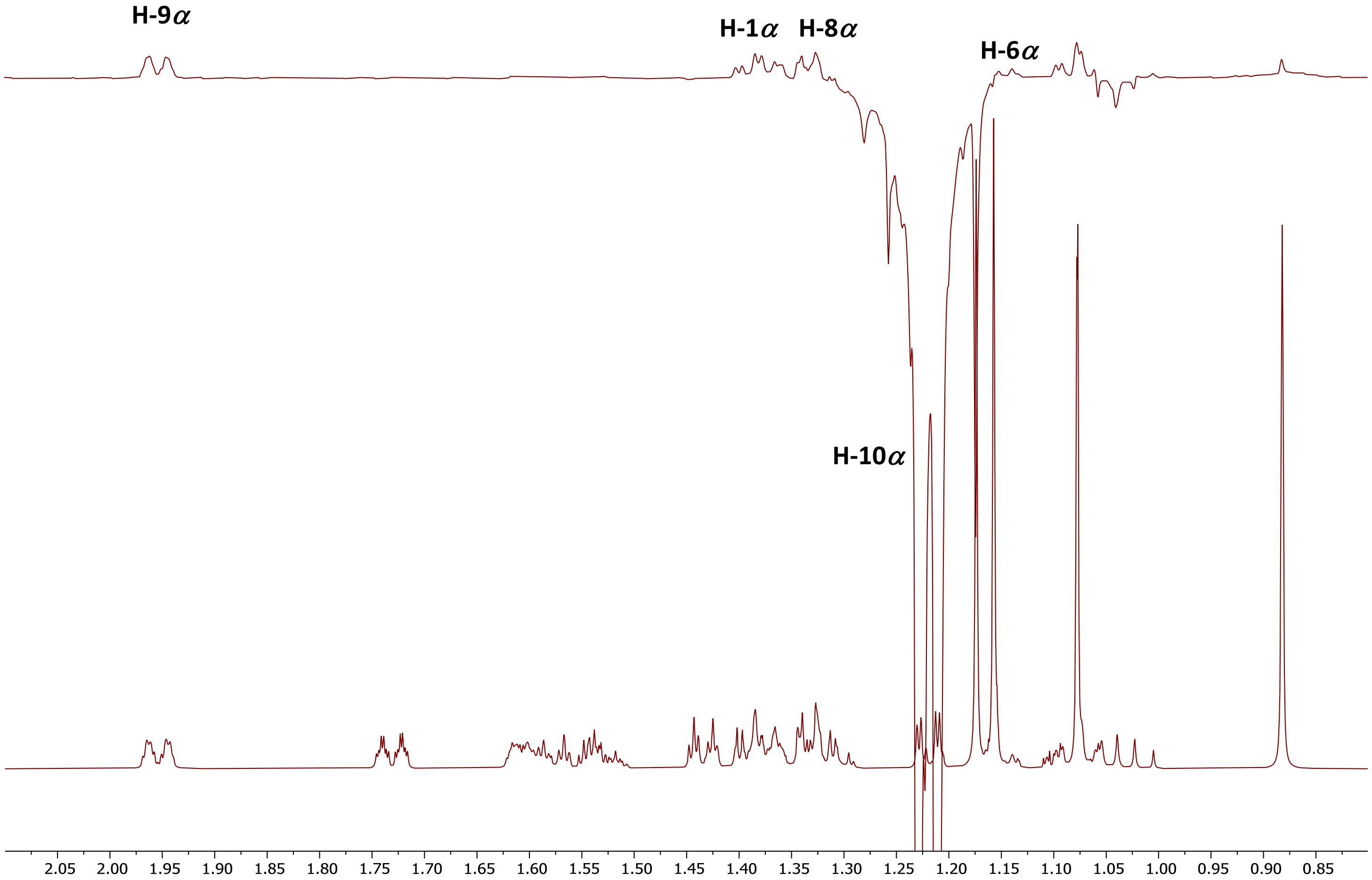

Figure S29c. 1D NOESY spectrum of compound **4**.

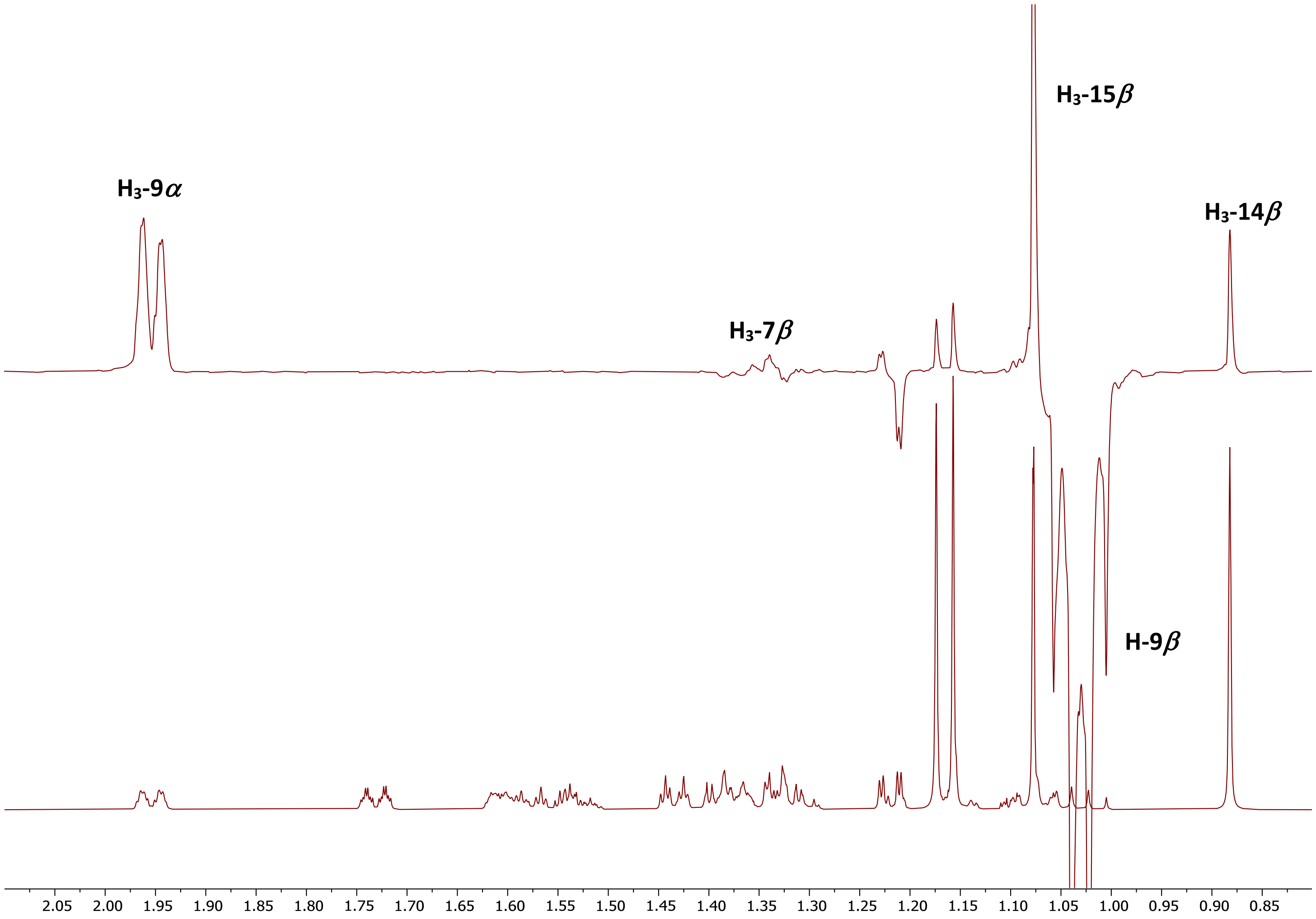

Figure S29d. 1D NOESY spectrum of compound **4**.

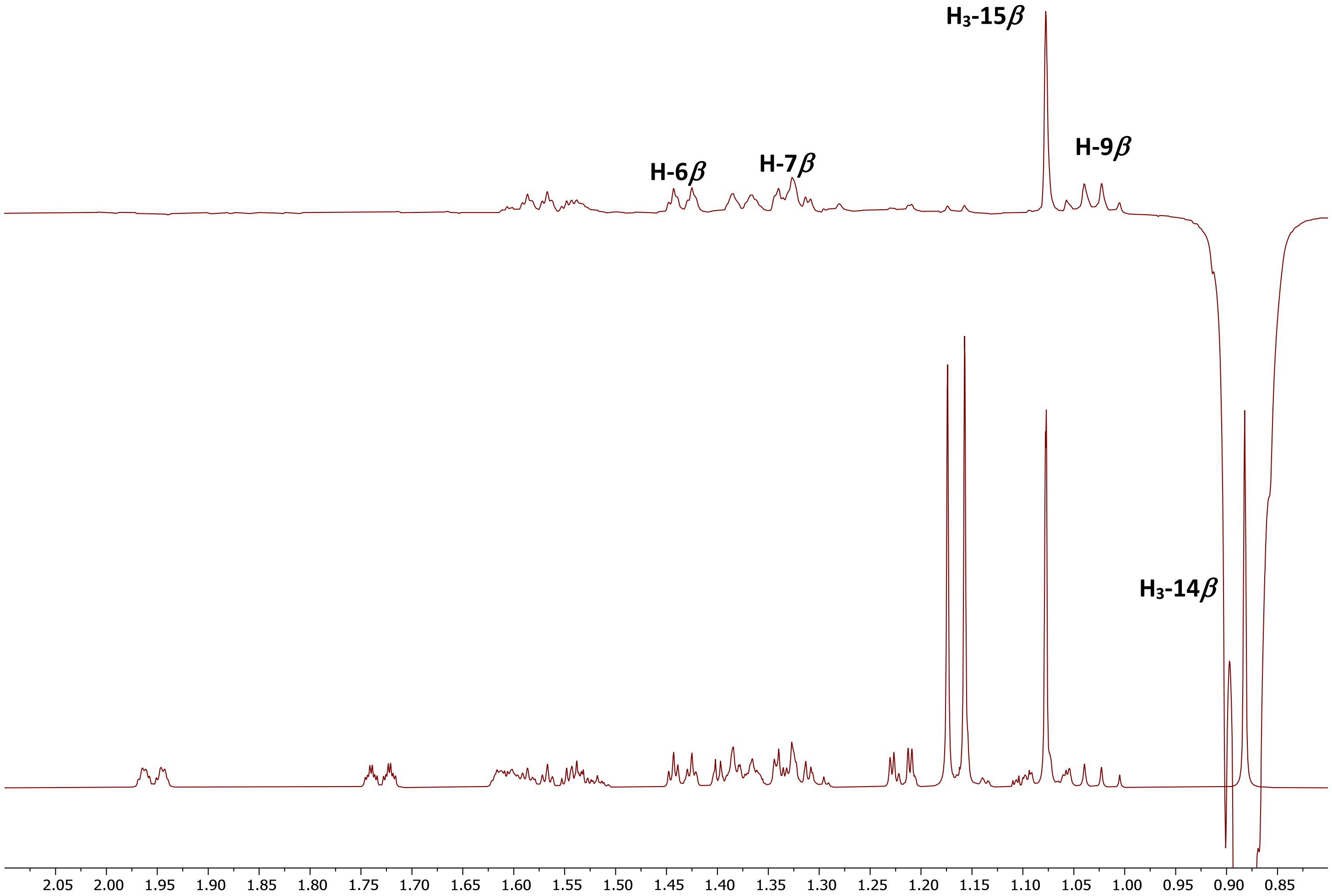

Figure S29e. 1D NOESY spectrum of compound **4**.

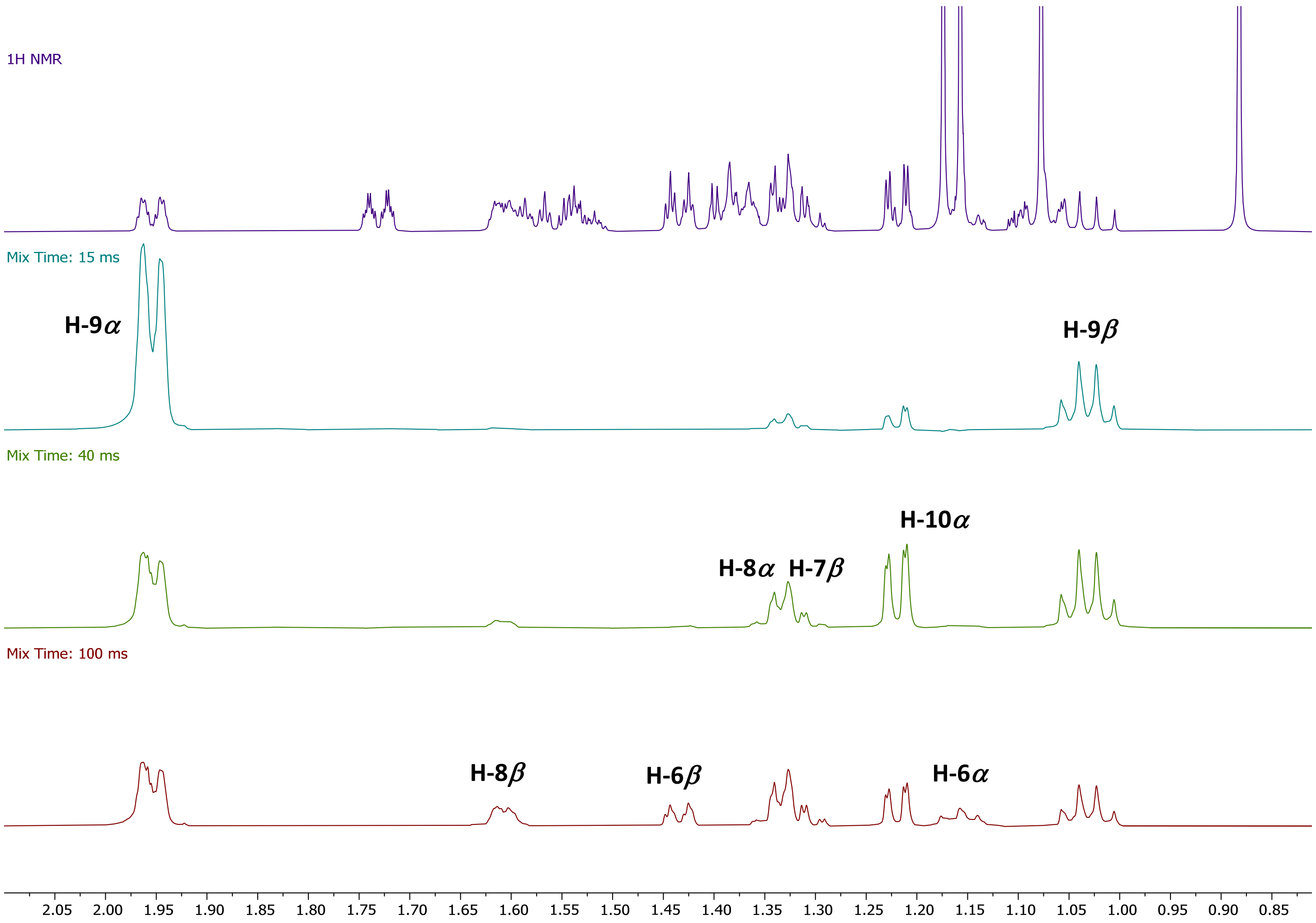

Figure S30a. 1D TOCSY spectra of compound **4**.

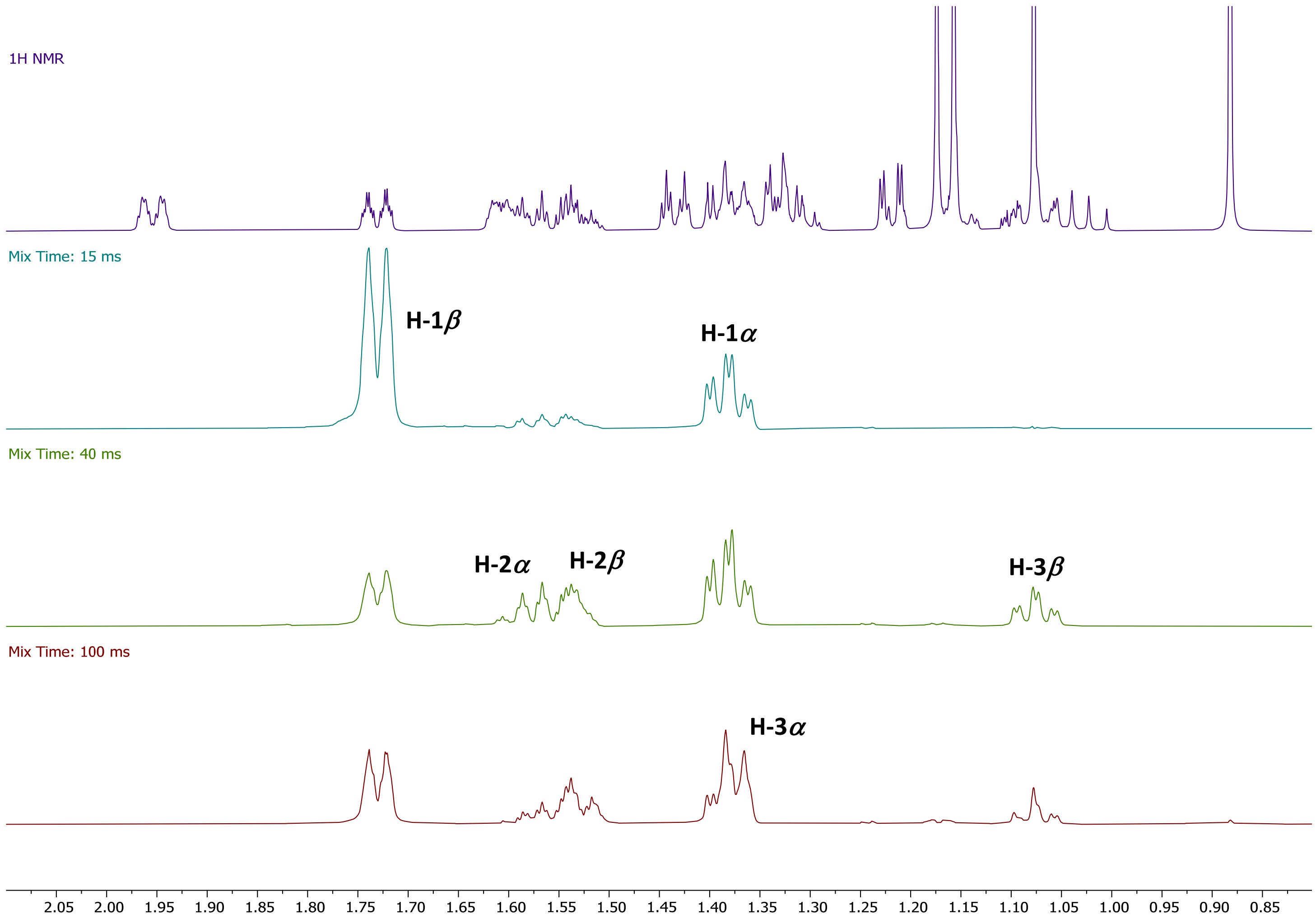

Figure S30b. 1D TOCSY spectra of compound **4**.

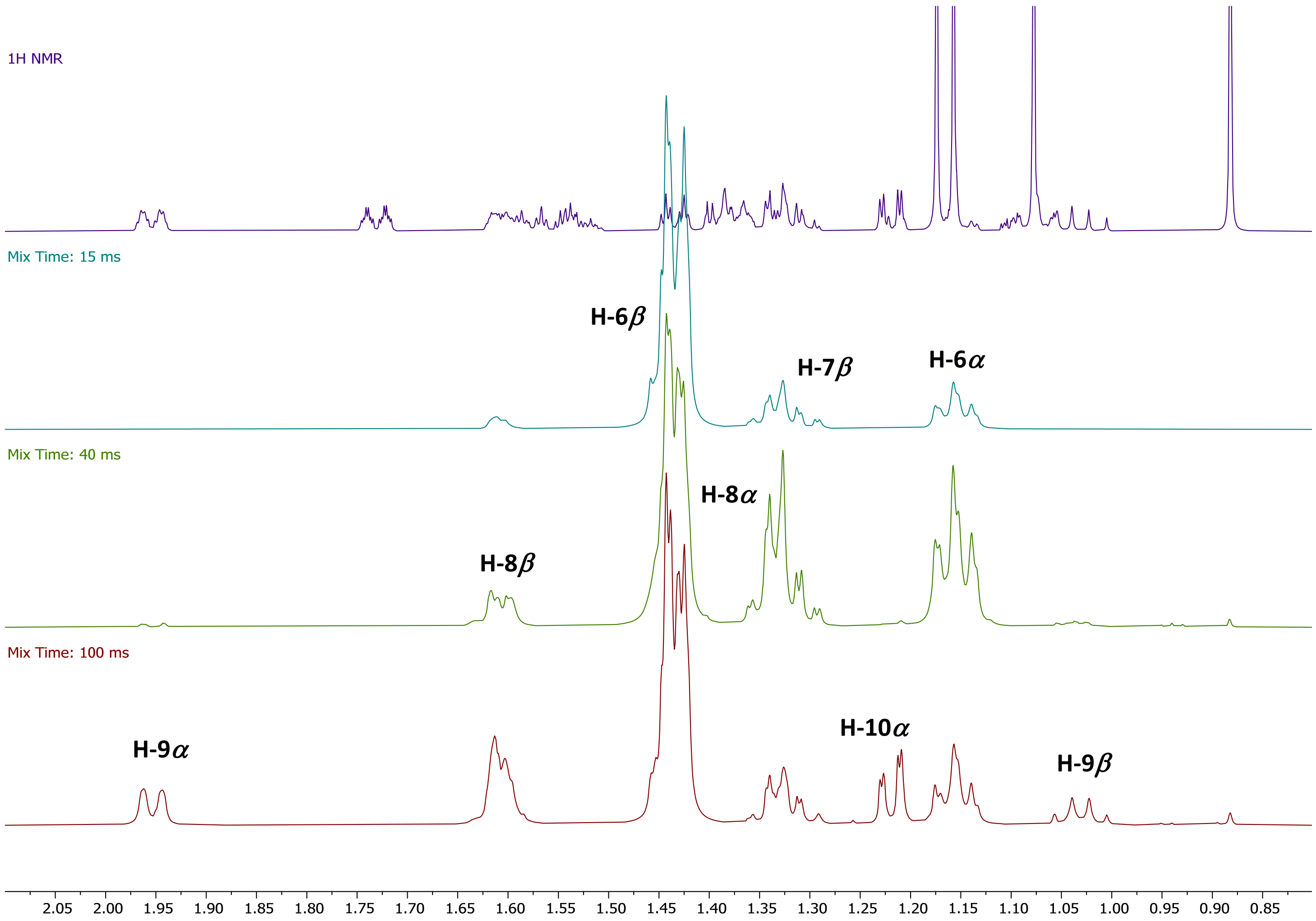

Figure S30c. 1D TOCSY spectra of compound **4**.

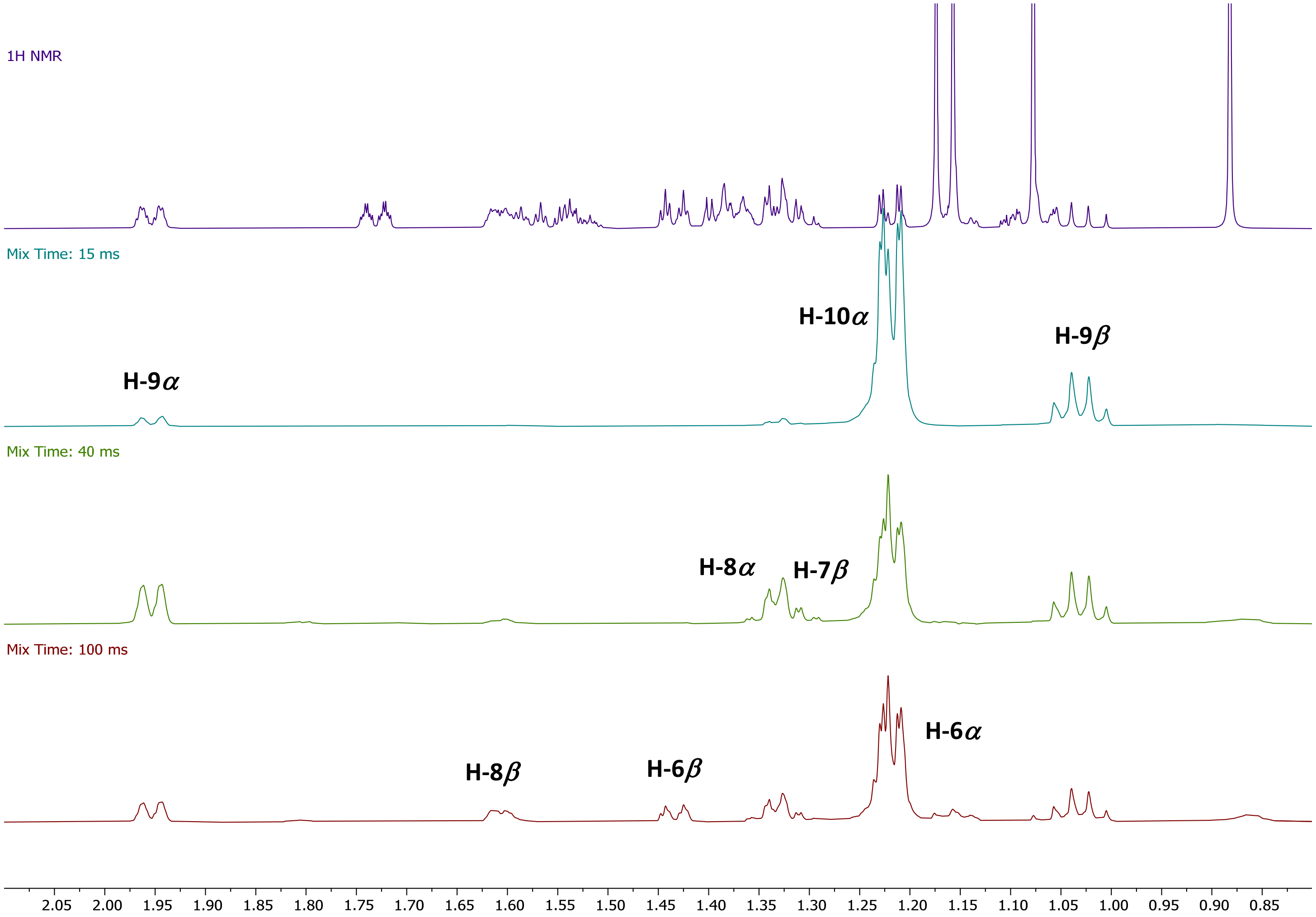

Figure S30d. 1D TOCSY spectra of compound **4**.

Single Mass Analysis

Tolerance = 3.0 mDa / DBE: min = -1.5, max = 80.0
Element prediction: Off
Number of isotope peaks used for i-FIT = 3

Monoisotopic Mass, Even Electron Ions
85 formula(e) evaluated with 2 results within limits (up to 5 closest results for each mass)
Elements Used:
C: 0-100 H: 0-100 O: 0-20 Na: 0-1
ROSA DURAN
CARLOS-16-F60 405 (4.658)

1: TOF MS ES+
2.75e+006

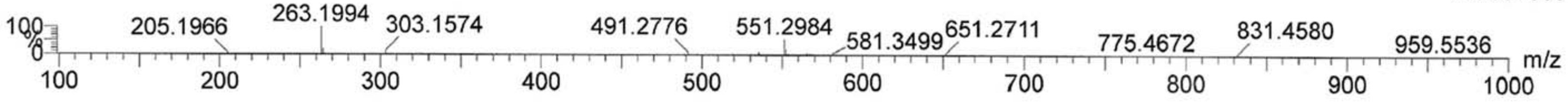

Minimum:				-1.5				
Maximum:		3.0	5.0	80.0				
Mass	Calc. Mass	mDa	PPM	DBE	i-FIT	Norm	Conf(%)	Formula
263.1994	263.1987	0.7	2.7	1.5	898.4	0.000	99.97	C15 H28 O2 Na
	263.2011	-1.7	-6.5	4.5	906.7	8.281	0.03	C17 H27 O2

Figure S31. HRESIMS spectrum of compound **4**.
